# Employment of Two-Acid Promoter System in Pictet–Spengler Reaction: A Robust Two-Step Synthesis of Aza-Heterocycles

**DOI:** 10.1021/acsomega.5c11423

**Published:** 2026-02-16

**Authors:** Dario Gentili, Gabriele Lupidi, Francesco Catalini, Alessio Petrellini, Vishnuprasad Ponnarassery Aravindakshan, Federico Vittorio Rossi, Alessandro Guzzini, Giacomo Di Giambattista, Cristina Cimarelli, Serena Gabrielli, Enrico Marcantoni

**Affiliations:** † School of Science and Technology, Chemistry Division, ChIP Research Center, 18959 University of Camerino, Via Madonna Delle Carceri, Camerino, MC 62032, Italy; ‡ 9309 University of Perugia, Department of Chemistry-Biology-Biotechnology, Via Elce di Sotto 8, Perugia, PG 06123, Italy; § School of Pharmacy, Drug Delivery Division, ChIP Research Center, 18959 University of Camerino, Via Madonna Delle Carceri, Camerino, MC 62032, Italy

## Abstract

The growing demand for increasingly sustainable chemical processes continues to stimulate organic chemistry to develop highly efficient, low-energy-consuming new reactions that require minimal effort to isolate intermediates and produce reduced waste. In this context, two-step reactions play an important role, and a good approach is the two-step synthesis, in which reagents and catalysts or promoters are introduced sequentially. Among these, the Pictet–Spengler-type cyclization (PS) is a powerful stepwise strategy for the synthesis of heterocyclic compounds. This article presents a novel and environmentally friendly two-step strategy for the Pictet–Spengler reaction, employing graphene oxide and Amberlyst 15 as acid promoters that yields a diverse product library (up to 92% yield) without the need for further purification. Both promoters can be reused over multiple reaction cycles, maintaining high efficiency. The methodology demonstrates broad functional group tolerance and provides a sustainable route to various tetrahydro-β-carbolines (THβCs), including biologically relevant compounds, and, above all, avoids negative interference between the two acidic promoters.

## Graphical abstract

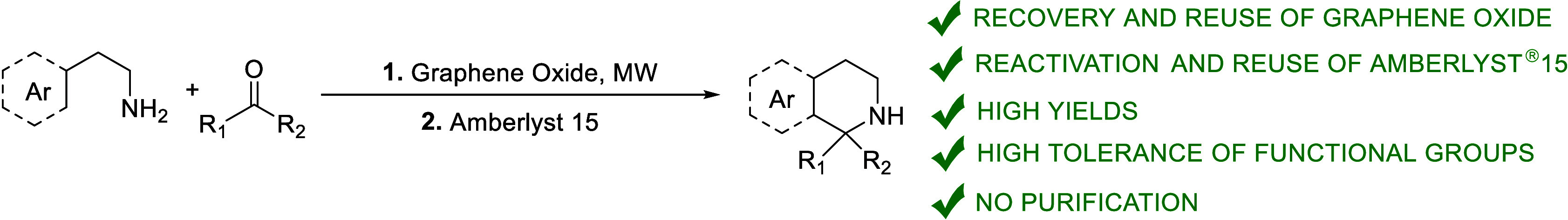

## Introduction

Many small heterocyclic molecules play a crucial role in everyday life due to their wide range of applications in human health^1^ and materials science.^2^ In particular, polysubstituted heterocyclic compounds containing one or more nitrogen atoms, natural and non-natural, are known to display potent biological activities,^3^ and they have been widely used as human therapeutics.^4^ Thus, these aza-heterocycles are significant in medicinal chemistry,^5^ and, although they occur naturally, these substances can also be synthesized^6^ in large amounts through industrial processes.^7^ Certainly, modern organic chemistry is a titan supporting in the synthesis of polysubstituted aza-heterocycles, which can occur either through C–H functionalization of an aza-heterocycle core or through cyclization of acyclic precursors.^8^ The methodology of cyclization of the appropriate acyclic precursor is assuming an ever-increasing importance, and Lewis acids proved to be relevant in facilitating this type of reaction.^9^

Small aza-heterocyclic molecules also constitute an important class of compounds frequently used as building blocks, for example, in the construction of numerous indole alkaloids having biological utility. Some years ago, our research group focused its attention on the synthesis of alkyl 9*H*-β-carboline-4-carboxylate **7** performing the cyclization of the corresponding acyclic precursor, prepared via a Lewis acid-catalyzed CeCl_3_ reaction.^10^ The carbolines (Cs) represent a family of biologically active alkaloids containing an indole moiety widespread in nature (Figure 1).^11^ For this reason, over the years, the Cs have proven to be useful drug candidates against a wide range of pathologies, and the research has mainly focused on β-carbolines (βCs) and their derivatives because they have proven to be rich in pharmacological activities, with a particular focus on anticancer properties.^12^

**Figure 1:**
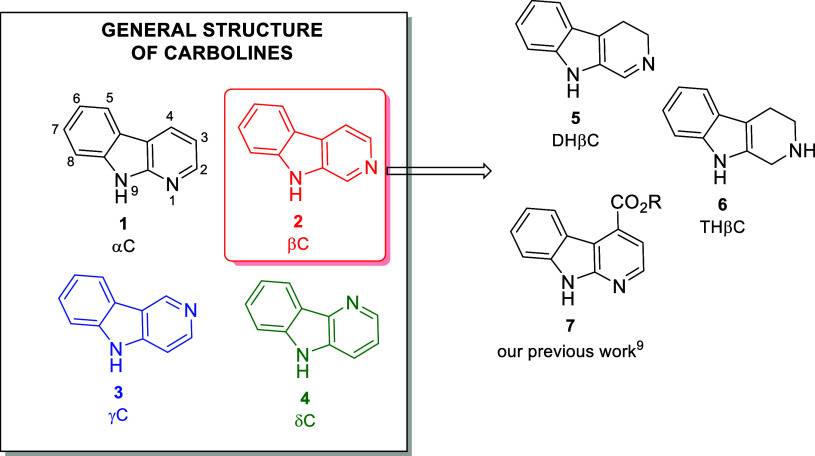
Parent frameworks for carbolines and partially saturated β-carboline analogues: αC (**1**), βC (**2**), γC (**3**), δC (**4**), DHβC (**5**), and THβC (**6**).

For this reason, the synthesis of polysubstituted βCs and their saturated derivatives is of special interest for organic chemistry, as several natural, commercial, and candidate drugs contain these moieties.^13^

From a biological and pharmacological perspective, βCs, dihydro-β-carbolines (DHβCs), and THβCs of higher complexity are of great interest. In fact, complex bioactive molecules containing βCs and THβCs, such as Abecamil and Manzamine, have been reported (Figure 2).^13^ The inhibitory activities of several βCs against the enzymes IDO1 (indoleamine 2,3-dioxygenase 1) and TDO (tryptophan 2,3-dioxygenase), both involved in the kynurenine pathway, have been evaluated.^14^ Abnormalities in this biological pathway can lead to depressive conditions. The study showed that the bioactivity of βC derivatives was strongly dependent on the functional groups introduced by the aldehyde used in the PS reaction step. For example, the presence of halogens significantly enhanced the inhibitory activity. Additionally, βCs containing a 1,2,3-thiazole moiety and imidazole exhibited strong inhibitory action.

**Figure 2:**
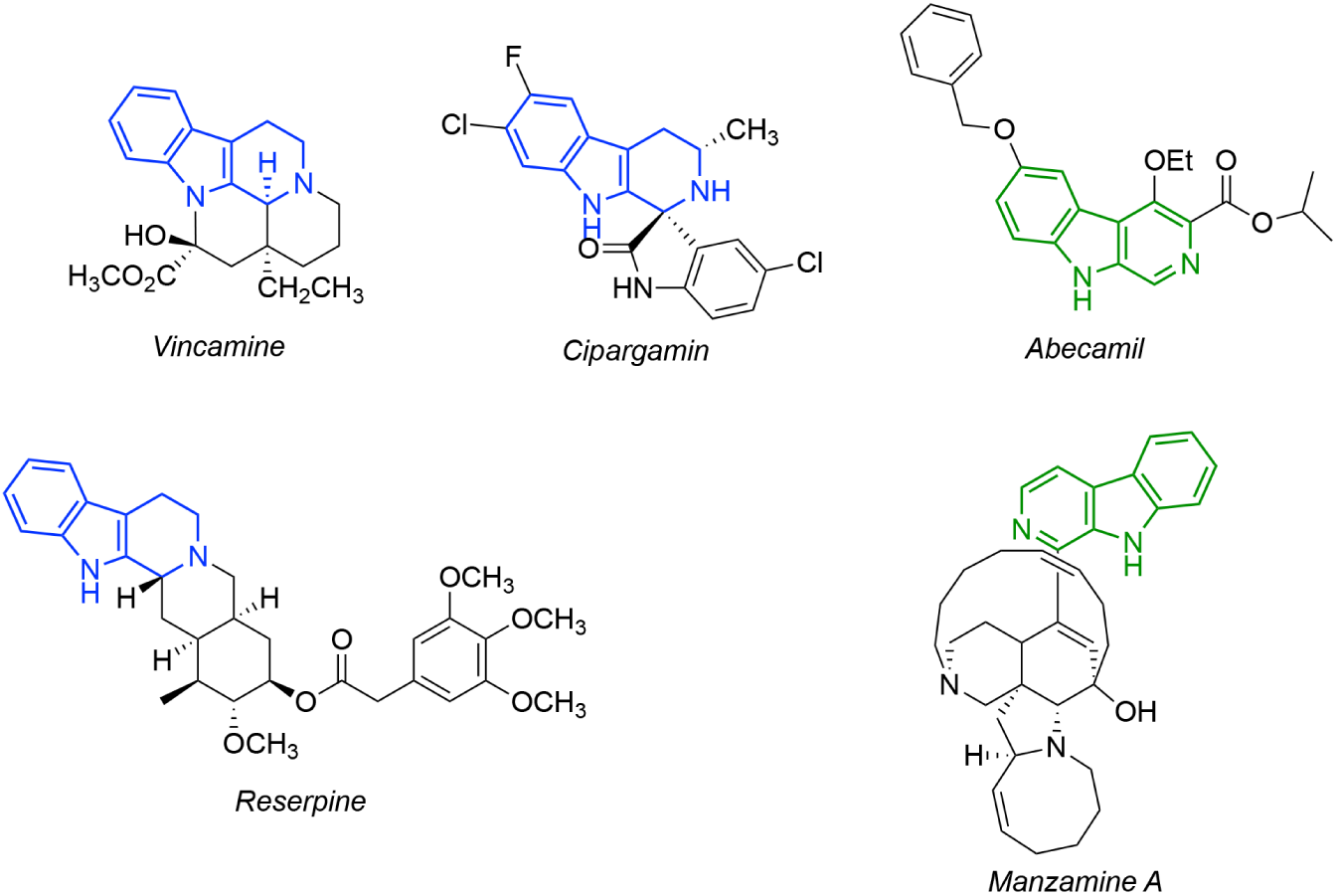
Some bioactive compounds with THβC and βC moieties.

The unique structure of THβCs, combining a rigid indole ring and a flexible piperidine moiety, allows them to act as both H-bond donors and acceptors, a highly desirable feature for drug design.^15^ In fact, THβCs core is present in many biologically active compounds widely employed as antioxidants,^16^ anticancer agents,^17^ antivirals,^18^ antileishmanial drugs^19^ and in the treatment of Alzheimer’s disease.^20^ Compared to Ribavirin (commercial antiviral agent), natural and synthetic THβCs and DHβCs demonstrated improved *in vivo* efficacy against Tobacco Mosaic Virus (TMV, Figure 3).^21^

**Figure 3:**
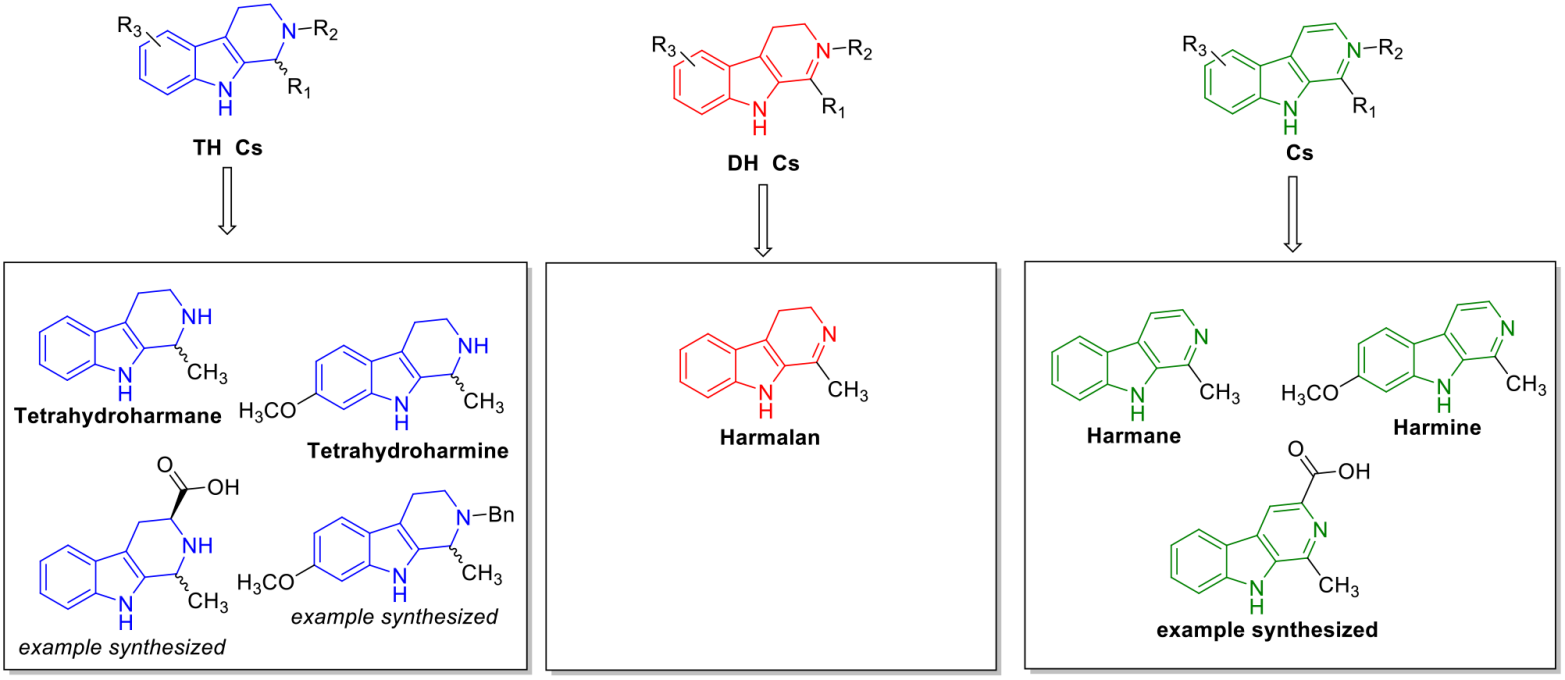
THβCs, DHβCs, and βCs studied by Song et al.

The most common pathway for the synthesis of this peculiar core is the Pictet–Spengler reaction.^22^ This acid catalyzed reaction between carbonyl compounds and β-phenylethylamines (tryptamines and phenylethylamines) provides the formation of THβCs, which, after further oxidation, leads to the βC skeleton.^23^ The mechanism of the PS reaction occurs in two steps: first, an imine is formed through the condensation of an amine and an aldehyde (or ketone), and then the imine is activated in an acidic environment, forming an iminium ion, which undergoes a 6-endo trig cyclization.^22d^ The formation of a new C–C bond produces a new heterocyclic compound from an acyclic intermediate. Finally, deprotonation restores the aromaticity of the aryl moiety.^24^

Over the years, significant interest in THβC synthesis has led to the development of new protocols. Still, these are often hindered by limited sustainability due to the need for harsh reaction conditions and strong acid catalysts. A survey of existing protocols for the PS reaction shows that this reaction is commonly performed in acidic environments using strong Brønsted acids such as HCl,^25^ acetic acid,^26^ trifluoroacetic acid,^27^ sulfuric acid,^28^ and boric acid,^29^ sometimes requiring long reaction times and/or high temperatures. A recent fascinating protocol has been reported employing the use of ion-pair tetrakis­(pentafluorophenyl)-borate (Ph_3_C^+^[B­(C_6_F_5_)_4_]^−^), which allows developing a metal-free strategy in water, thanks to the *in situ* formation of the superacid H_3_O^+^[B­(C_6_F_5_)_4_]^−^, showing a better catalytic activity than H_2_SO_4_.^30^ Despite providing products in good yields with aryl aldehydes and cyclic ketones, the reaction demands a costly catalyst, 12 h reaction time at high temperature (100 °C) and a final chromatographic purification to achieve target products. Another Pictet–Spengler protocol using trifluoroacetic acid as a 10% solution in water is reported. Several products were isolated (with yields ranging from 45% to 83%) using tryptophan methyl ester, *N*-alkylated tryptophan methyl ester, and tryptamine after prolonged incubation (24 h to 36 h) at room temperature in the presence of a stoichiometric amount of aldehyde. The choice of TFA was led by the observation that, in their investigations, commonly used Brønsted or Lewis acids such as acetic acid, *p*-toluene sulfonic acid, and Yb­(OTf)_3_ were less effective (i.e., 23% with AcOH) or utterly inactive (*p*-TsOH and Yb­(OTf)_3_).^31^

We investigated a dual-catalyst approach using graphene oxide and a well-established heterogeneous organic synthesis catalyst as Amberlyst 15, to overcome these limitations.^32^ This new methodology offers promising pathways for more sustainable and eco-friendly organic syntheses, aligned with modern green chemistry goals (Figure 4).

**Figure 4:**
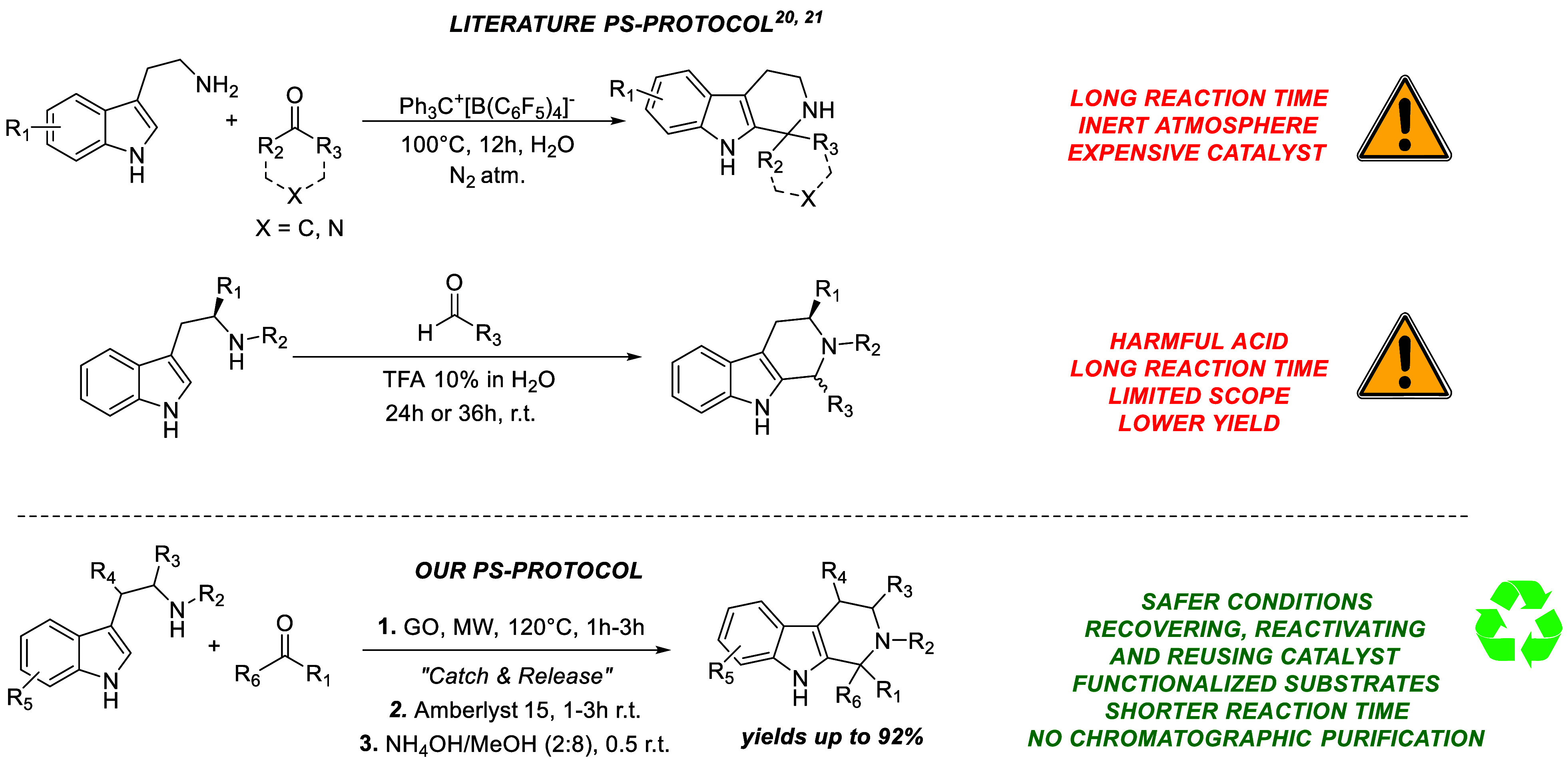
Synthetic protocols for the preparation of tetrahydro-β-carbolines.

Graphene Oxide (GO) and other carbonaceous materials have emerged in recent years as promising carbon-based catalysts for developing new green and sustainable synthetic pathways. The intrinsic Brønsted acidity of GO arises from the presence of several alcohol, ketone, epoxide, and carboxylic groups bonded to the 2D layers of graphene, which enables this material to catalyze reactions such as transamidations, the Fischer and Kabachnik–Fields reactions and many other organic transformations.^33^ Notably, in 2014, it has been demonstrated that NaOH-deactivated graphene oxide exhibited no activity in a condensation reaction between glyoxal and 4-hydro coumarins in water after 30 min, underlining the importance of acidic moieties in GO nanosheets.^34^ Furthermore, its excellent dispersibility in solvent media, high stability, recyclability, safety, and insolubility contribute to its prominence as a versatile heterogeneous catalyst.

The effectiveness of acidic ion exchange resins in catalyzing PS reactions has already been proved:^35^ It is reported that Dowex 50W-X4 is the most active resin in a protocol involving a “catch and release” step for isolating PS products. However, that study was limited to tryptamine as the sole amine, required a 10-fold excess of the aldehyde partner, and yielded a maximum of 43% in dichloroethane at 90 °C. They also noted that, under the conditions they explored, Amberlyst 15 did not promote any product formation. Amberlyst 15 is a commercially available, strongly acidic ion-exchange resin widely used in acid-catalyzed organic transformations. It is a sulfonic acid-type styrene/divinylbenzene copolymer, and its higher activity can be explained by its physical properties, such as its H^+^ capacity (4.2 mequiv/g) and high surface area (42 m^2^/g).^36^ Amberlyst 15 behaves as a strong acid and can be easily removed from the reaction mixture by filtration, then reactivated and reused multiple times.^37^ This resin has attracted attention in the scientific field for its unique and green properties, including environmental compatibility, reusability, noncorrosiveness, chemical and physical stability, and nontoxicity, alongside its high performance in several reactions.^38^ Due to these unique properties, Amberlyst 15 has found applications in many reactions.^39^ To the best of our knowledge, only one protocol has already been reported that employs Amberlyst 15 in a Pictet–Spengler reaction in the presence of a chiral thiourea derivative, yielding the PS product with only 21% yield after 48 h and good enantioselectivity.^40^

In this paper, we present a novel, clean, and environmentally friendly protocol for synthesizing functionalized tetrahydro-β-carbolines via the Pictet–Spengler reaction, employing a two-step protocol that condenses substituted tryptamine and tryptophan derivatives with various aldehydes. This procedure utilizes graphene oxide under microwave irradiation^41^ for the initial imine formation step, followed by Amberlyst 15 treatment for the cyclization and product entrapment. The final product is released through a “catch and release” step, eliminating the need for chromatographic purification.

## Result and Discussion

Our first investigation began with evaluating the potential use of CeCl_3_·7H_2_O as a catalyst in the Pictet–Spengler reaction. Our research group has previously intensively studied the catalytic activity of this Ce­(III) salt, during which imine intermediates are formed and participate in the reaction.^42^ As a first attempt, we investigated the reaction between tryptamine **8a** and benzaldehyde **9a**. When the reaction was carried out under reflux in dichloromethane (DCM) in the presence of 0.3 equiv of CeCl_3_·7H_2_O and 0.3 equiv of CuI, only imine **10a** was formed. Switching from batch to microwave conditions, product **11a** was isolated in 60% yield. When the reaction was performed in the absence of any catalyst, only the formation of the imine was detected (Scheme 1).

**Scheme 1:**
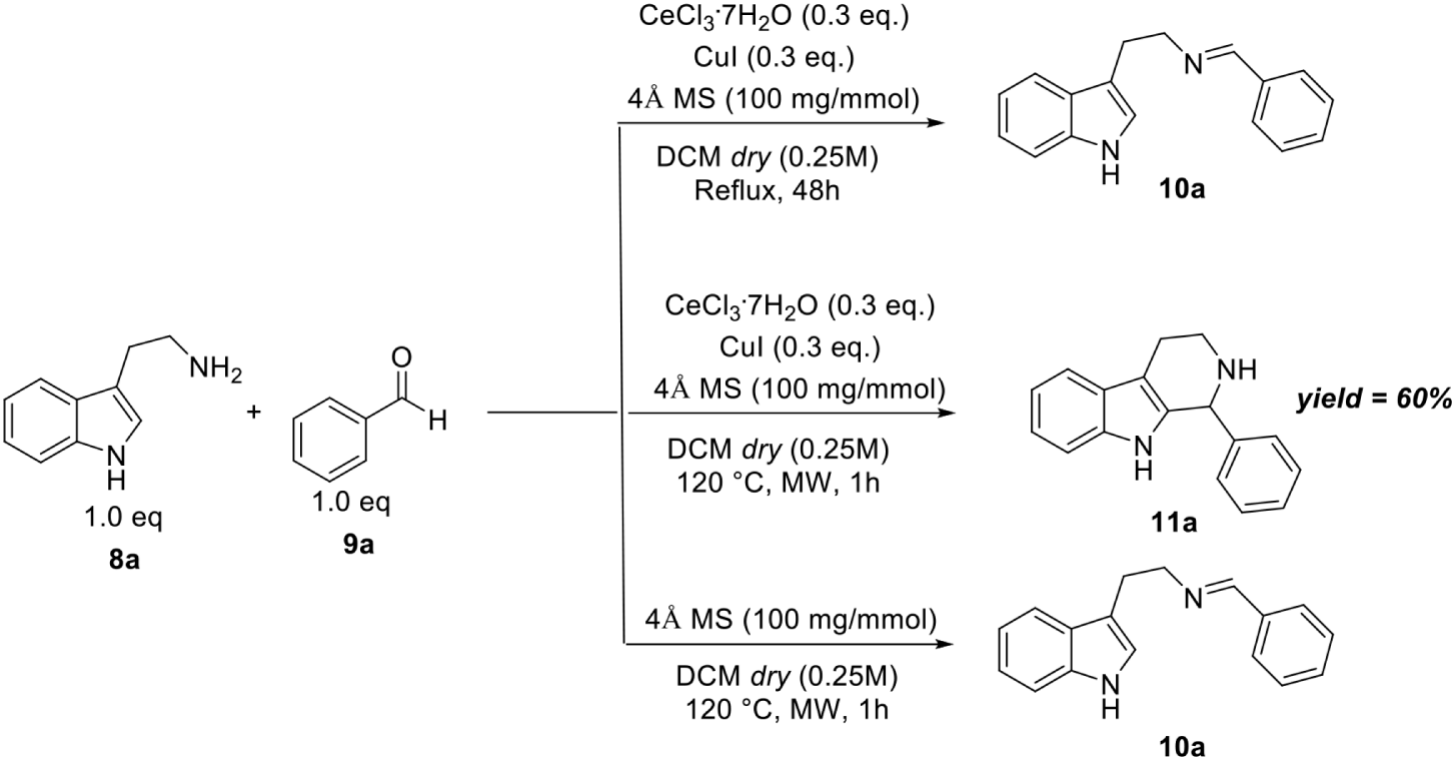
Pictet–Spengler Cyclization CeCl_3_·7H_2_O–CuI Catalyzed and Microwave Assisted

The enhancement of the trivalent cerium salt catalytic activity could explain the higher yield using the CeCl_3_·7H_2_O/CuI system, since it is now accepted that the chlorine-bridged oligomeric structure of CeCl_3_·7H_2_O^43^ is easily broken by donor species such as the iodide ion.^44^

To assess the feasibility of performing the reaction in a one-pot process, we conducted an experiment using 1.0 equiv of tryptamine, benzaldehyde, and benzyl chloride in dry DCM (0.25 M) under microwave irradiation at 120 °C; the product **11b** was isolated in 55% yield (Scheme 2).

**Scheme 2:**
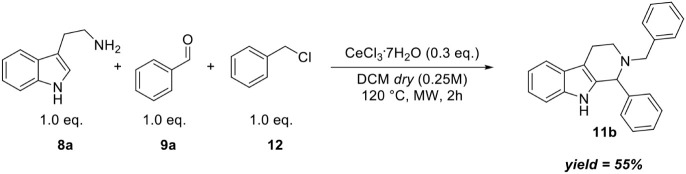
One-Pot Pictet–Spengler Reaction Ce­(III) Salt Catalyzed

The precise mechanism is not fully established, as cerium trichloride has been reported to work through pathways going beyond a simple Lewis acid mechanism. It has been reported that it can act as a promoter of the initiation of the reaction, which then proceeds via a Brønsted acid mechanism,^45^ so we drastically changed the approach, testing a new one-pot protocol to obtain the final product. In fact, we synthesized the imine **10a** under microwave irradiation using GO as a Brønsted acid. Then, we exploited the acidic moiety of Amberlyst 15 as a selective extraction tool for product **11a**, as reported in Scheme 3. The product was obtained in 53% yield.

**Scheme 3:**
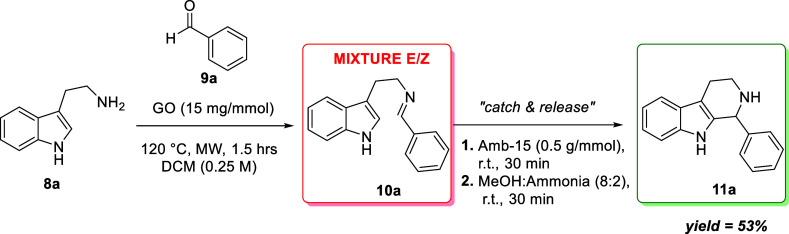
Pictet–Spengler Reaction with a Two-Acid Promoter System

Although products **10a** and **11a**, have the same molecular weight, we can distinguish the imine from the cyclized product because the first has a typical and most abundant fragment 130 *m*/*z* (C_9_H_8_N^+.^, indole moiety) while the cyclized product has a 171 *m*/*z* ion (C_11_H_11_N_2_^+.^) derived from the loss of a phenyl moiety that leads to the formation of a very stable fragment. This fragmentation is possible only in the structure of the product **11a.** ^1^H NMR studies confirmed this hypothesis, as the imine exhibits two triplets with 2H integration, one at 3.87 ppm and the other at 3.18 ppm. Formation of the product (**11a**) is also underlined by a characteristic singlet of −C*H*- at 5.09 ppm (SI, pages 4–6).

Encouraged by this finding, two batch reactions were conducted using Amberlyst 15 as the sole catalyst to avoid potential degradation under microwave irradiation. The reaction in refluxing DCM afforded product **11a** in 42% yield, with substantial recovery of starting material. When acetonitrile (ACN) was used instead, the yield increased to 48% (Scheme 4).

**Scheme 4:**
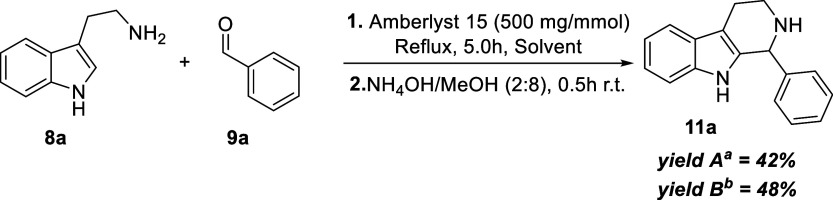
Pictet–Spengler Reaction Performed with Only Amberlyst 15

So, at this point, we wanted to join the two processes: first synthesizing the imine **10a** in the presence of GO under microwave irradiation and then perform the cyclization in the presence of Amberlyst 15; the optimization experiments are reported in Table 1.

**Table 1:**
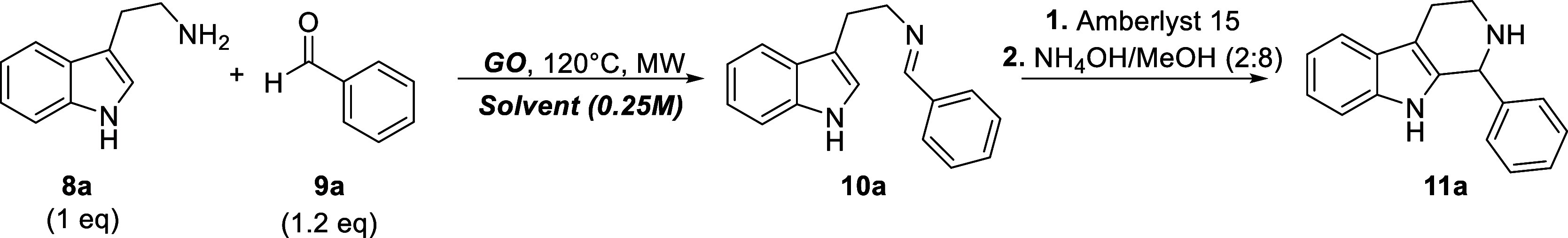
Optimization Conditions of Reaction

Entry	Time (h)	GO (mg/mmol)	Solvent	**11a** (yield %)
**1**	1.5	15	DCM	53
**2**	1.5	15	ACN	82
**3** ^a^	16	15	ACN	51
**4**	1.5	15	EtOH abs	69
**5**	1.5	15	EtOAc	73
**6**	1.0	15	ACN	87 (86)^j^
**7** ^b^	1.0	15	ACN	70
**8** ^c^	1.5	15	ACN	75
**9**	1.0	-	ACN	48
**10**	1.0	7.5	ACN	72
**11**	1.0	30	ACN	83
**12** ^d^	1.0	15	ACN	58
**13** ^e^	1.0	15	ACN	80
**14** ^f^	1.0	15	ACN	58
**15** ^g^	1.0	15	ACN	40
**16** ^h^	1.0	15	ACN	77
**17** ^i^	1.0	-	ACN	-
**18** ^i^	1.0	15	ACN	-

a Reaction performed in batch at reflux temperature.

b Reaction performed with 1.0 equiv of **2a**.

c Reaction performed in the presence of 4 Å M.S.

d The mixture ACN/Amberlyst 15 was refluxed for 1 h and then the resin was washed with the solution MeOH/NH_4_OH_aq_.

e Reaction performed at 90 °C.

f Reaction performed at 150 °C.

g Loading of Amberlyst 15 was 200 mg/mmol.

h Loading of Amberlyst 15 was 1000 mg/mmol.

i Reaction in absence of Amberlyst 15.

j Reaction performed on 5 mmol scale.

Changing the solvent to ACN and performing the reaction (Table 1, entry 2) yielded **11a** in good yield. In order to confirm the power of microwave irradiation, we performed the first step also in batch, under reflux of ACN. After 16 h, GC and TLC monitoring indicated the complete consumption of the amine, allowing us to proceed to the second step with Amberlyst 15. The resin was washed with a solution of MeOH/NH_4_OH_aq_ (8:2), yielding product **11a** and secondary products, which were not isolated (Table 1, entry 3). Since experiments with other solvents did not yield better results (Table 1, entries 4–5), we decided to maintain ACN as the reaction solvent. Reducing the reaction time to 1 h had no significant impact on the yield, affording the product in 87% yield (Table 1, entry 6). However, a slight erosion in yield was observed when the aldehyde equivalents were reduced (Table 1, entry 7). A similar trend was observed when activated 4Å molecular sieves were added to trap the water formed during imine formation in the first step (Table 1, entry 8). Variations in reaction temperature and GO and Amberlyst 15 amounts were found to negatively affect the reaction outcome (Table 1, entries 9–16). Notably, the reaction conducted without GO addition still yielded 48% product (Table 1, entry 9). In contrast, in the absence of Amberlyst 15 (Table 1, entry 18), the reaction halts at the first step involving imine formation, without achieving intramolecular cyclization.

Next, we focused our attention on the final step of the catch-and-release process. Various purification methods were tested by washing the ionic resin with different basic solutions, as reported in Table 2, using the GO-oxide protocol and a loading of 500 mg/mmol of Amberlyst 15.

**Table 2:**
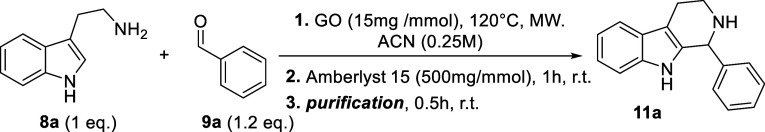
Optimization of “Catch and Release” Purification Step

ENTRY	Purification	Yield **11a** (%)
**1**	8:2 MeOH/NH_4_OH (10 mL/mmol)	87
**2**	8:2 MeOH/Et_3_N (10 mL/mmol)	70
**3**	2.0 M of NH_3_ in MeOH	75
**4**	8:2 MeOH/Et_3_N (10 mL/mmol) + filtration on Florisil	16

Using methanol/ammonia solution (Table 2, entry 1) proved to be the most efficient purification method, yielding 87% of product **11a** (SI, pages 4–6), although it required a solvent/solvent extraction (DCM/sat. NaHCO_3_(aq). In contrast, the use of trimethylamine in methanol (Table 2, entry 2) led to a tedious workup due to difficulty in evaporating the amine itself. Also, the use of a high molarity NH_3_ solution in methanol appeared promising; however, it was still less effective than the MeOH/NH_4_OH system (Table 2, entry 3). Filtration through Florisil alone resulted in a low yield, likely due to its intrinsic acidity, which retains both the product and triethylamine, even after extensive washing with multiple volumes of DCM and MeOH. (Table 2, entry 4). With all steps of this new optimized protocol in hand, we proceeded to explore various carbonyl compounds and tryptamine derivatives.

Scheme 5 summarizes the results obtained using the general protocol, which was effective for both alkyl and aryl aldehydes. Products were isolated in high yields when electron-withdrawing groups were present on the phenyl ring, such as in **11c** (SI, pages 10–12) and **11e** (SI, pages 16–18). However, a methoxy group (**11d,** SI, pages 13–15) led to a 69% yield, likely due to deactivation of the carbonyl moiety. Notably, compounds **11e** and **11g** (SI, pages 16–18, 22–24, respectively) were isolated in higher yields than those reported in literature. It is known^46^ that these compounds were synthesized using a significant excess of TFA in toluene after 2 days of reaction and they exhibit good antitumor activity against P-388, KB-16, A549, and HT-29 cell lines.^47^ The product **11m** (SI, pages 41–43) derived from butanal, was also isolated. Still, chromatographic purification was necessary due to the formation of aldehyde autocondensation products, along with their corresponding imines and final PS products. Interestingly, product **11i** (SI, pages 28–30), resulting from the reaction between tryptamine and acetophenone, was successfully isolated. This result is notable since ketones are known to be less reactive in the Pictet–Spengler reaction (PS)^46^ typically requiring very harsh conditions^38^ and extended reaction times.^48^ Compound **11j** (SI, pages 38–40) was obtained after refluxing the corresponding imine for 5 h in the presence of Amberlyst 15 because the reaction conducted at room temperature afforded very low conversion of the imine to the product. Particularly intriguing is the synthesis of THβC **11k** (SI, pages 35–37), an important precursor to perloryline, a molecule bearing a β-carboline skeleton with proven activity against stomach tumor cells that appears also to act as an activator of TRPV1 (transient receptor potential vanilloid 1), a protein involved in regulating human pain perception.^49^ In the literature, **11k** has been synthesized using trifluoroacetic acid (a harmful Brønsted acid) after 2 h of reaction at room temperature, achieving a similar yield.^50^

**Scheme 5:**
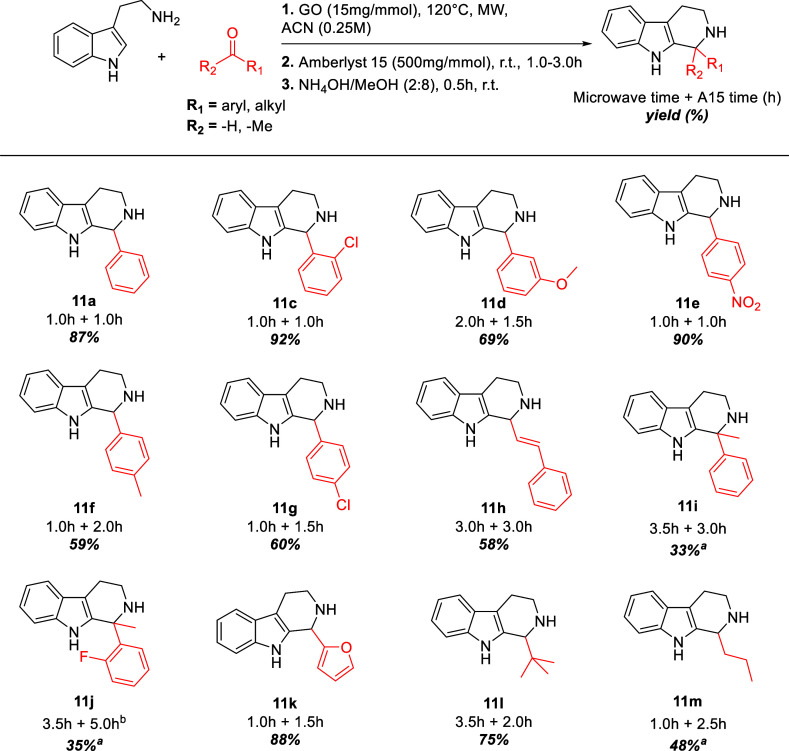
General Protocol for Carbonyl Compounds Screening

NOTE under Scheme 5: ^a^Product required chromatographic purification. ^b^ Product was isolated after the reaction mixture was refluxed with Amberlyst 15 for 5h. Next, we investigated various tryptamine derivatives, as shown in Scheme 6. Products were successfully isolated with good yields (42–75%) for all tryptamine and tryptophan derivatives, including secondary tryptamines such as *N*-benzyl tryptamine (**11b**, SI, pages 7–9). Unfortunately, reactions with benzaldehyde and butanal (**11y** and **11z**) resulted only in the formation of the corresponding imines, with no ring closure observed. This outcome is likely due to the reaction conditions that are not suitable for the indole system derivatization. The protocol also proved efficient for the methyl ester of tryptophan (**11ua**/**11ub**, SI, pages 65–67), yielding the product as a diastereomeric mixture (*syn*: 55%, *anti*: 45%), determined by ^1^H NMR analysis and cross-referenced with data reported in the literature,^51^ with an overall yield of 55%.

**Scheme 6:**
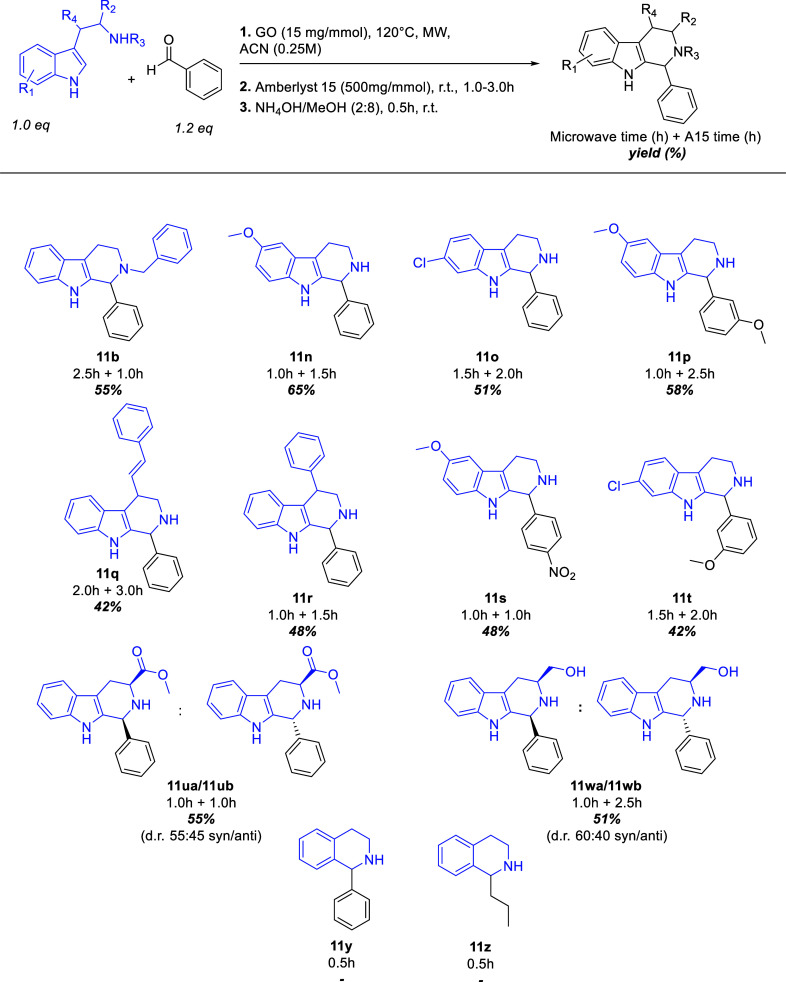
General Protocol for Different Tryptamines Screening

Additionally, the two diastereomers of l-tryptophanol (**11wa**/**11wb**, SI, pages 68–79) were isolated with a combined yield of 51%. Notably, the major diastereomer isolated was the *syn* form, with its structure confirmed by ^13^C NMR and NOESY analyses^52^ (SI, pages 4–79).

It is well documented in the literature that both GO and Amberlyst 15 can be recovered and reactivated.^37,51,53^ The recovery and reusability of graphene oxide were evaluated by monitoring the GC conversion of the imine, while Amberlyst 15 was assessed based on the final product yield (Table 3). Recovering GO was challenging because filtration through paper or Gooch filters led to significant catalyst loss. However, the catalyst was collected by washing the microwave reaction vial with fresh ACN, then centrifuged and washed with fresh reaction solvent. The recovered GO was then dried in an oven at 60 °C for 18 h (SI, page 3). In contrast, Amberlyst 15 was easily recovered by filtration and reactivated by stirring the resin in 2.0 N HCl for 30 min (SI, page 3), then a sequential washing was made with deionized water, ethanol, and acetone, and finally dried in an oven at 50 °C for 16 h.

**Table 3:**
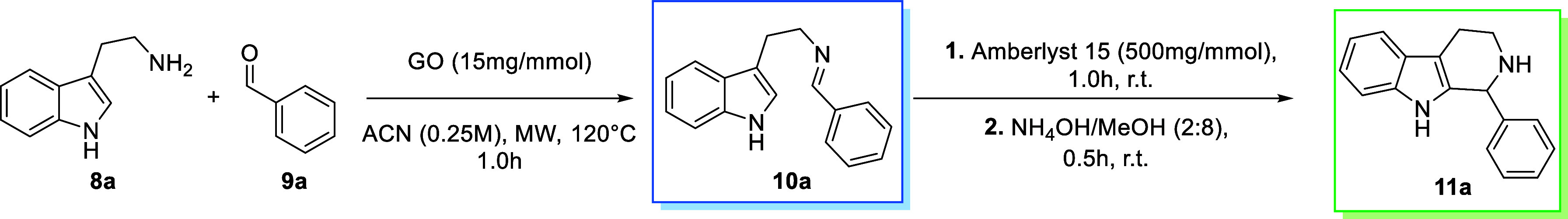
GC-Conversion and Yield of Reuse of Catalysts

Run	**10a** GC-conversion (%)	**11a** Yield (%)
**1**	98%	87%
**2**	98%	84%
**3**	97%	82%
**4**	93%	80%
**5**	90%	75%

We observed that the catalytic activity of GO remained consistent for five runs (>90% conversion). Similarly, Amberlyst 15 retained its catalytic efficiency for four runs but showed a slight erosion with the product yield decreasing to 75% after the fifth reaction (Figure 5).

**Figure 5:**
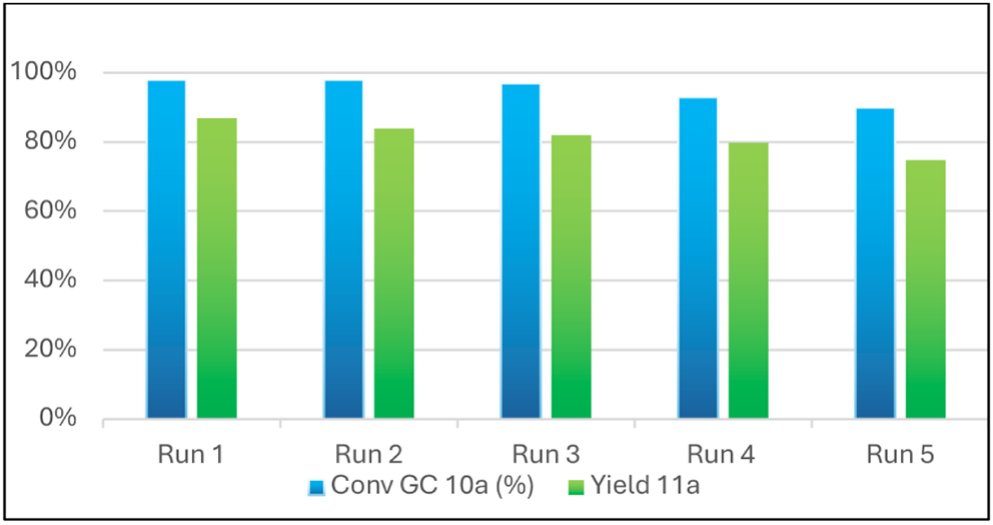
Recovery of graphene oxide and Amberlyst 15.

## Conclusion

We have developed a novel methodology for the Pictet–Spengler reaction that combines in a multistep process the utility of Graphene Oxide and Amberlyst 15, two inexpensive, nontoxic, easily handleable, recoverable, and recyclable reagents. This protocol relies on the dual role of the ionic resin, which functions both as a Brønsted acid catalyst and as a purification agent. The resin extracts the product from the reaction mixture, and the subsequent washing with a basic solution enables the isolation of a diverse library of THβC compounds. The protocol is advantageous due to the ease of handling of the reagents, the simple workup procedure, and the environmentally benign conditions that allow effective recyclability. Furthermore, it has demonstrated broad functional group tolerance and, in some cases, offers an alternative pathway for synthesizing biologically important aza-heterocyclic compounds.

The recovery investigations confirmed that GO and Amberlyst 15 can be reused for at least four runs without significant loss of activity as acid promoter system. Further studies are underway in our laboratories to improve the sustainability of organic reactions, with particular concern to the use of substances, promoters, or enhancers, added to a reaction to improve the efficiency or selectivity of a catalyst. We are currently expanding our methodology to cover other synthetically useful acid catalyzed organic chemistry processes in the synthesis of small molecules

## Supplementary Material



## Data Availability

All data generated during this study are included in the article and its Supporting Information.

## Notes

General information, reaction conditions, materials, full characterization by FT-IR, MS, and melting point data, and ^1^H, ^13^C, ^19^F and NOESY NMR spectra for compounds **11a–11wb** (PDF)
