# Supplementary material for: Employment of Two-Acid Promoter System in Pictet–Spengler Reaction: A Robust Two-Step Synthesis of Aza-Heterocycles
Source: ACS Omega. 2026 Feb 16;11(8):13891–901. doi: 10.1021/acsomega.5c11423 (PMC12961566; doi:10.1021/acsomega.5c11423)
Supplement: Supplementary file 1 [file ao5c11423_si_001.pdf]

Supporting Information

Employment of Two Acid Promoter System in Pictet-Spengler Reaction: a Robust Two-Step Synthesis of Aza-Heterocycles

Dario Gentili, †a Gabriele Lupidi, †a Francesco Catalini,[b] Alessio Petrellini,[b] Vishnuprasad Ponnarassery Aravindakshan,[a] Federico Vittorio Rossi,[a] Alessandro Guzzini,[a] Giacomo Di Giambattista[c] Cristina Cimarelli,[a] Serena Gabrielli, *a Enrico Marcantoni.[a]

[a]*School of Science and Technology, Chemistry Division, University of Camerino, ChIP Research Center, Via Madonna delle Carceri, 62032 Camerino (MC), Italy*

[b]*University of Perugia, Department of Chemistry-Biology-Biotechnology, Via Elce di Sotto 8, 06123, Perugia (PG), Italy*

[c]*School of Pharmacy, Drug Delivery Division, University of Camerino, ChIP Research Center, Via Madonna delle Carceri, 62032 Camerino (MC), Italy*

email: serena.gabrielli@unicam.it

Table of Contents

Materials **3**

General methods for the synthesis of THβCs (11a – 11wb) **3**

General method for recovering GO **3**

General method for recovering A15 **3**

Characterization of THβCs (11a) **4-6**

Characterization of THβCs (11b) **7-9**

Characterization of THβCs (11c) **10 – 12**

Characterization of THβCs (11d) **13 – 15**

Characterization of THβCs (11e) **16 – 18**

Characterization of THβCs (11f) **19 – 21**

Characterization of THβCs (11g) **22 – 24**

Characterization of THβCs (11h) **25 – 27**

Characterization of THβCs (11i) **28 – 30**

**Characterization of THβCs (11j)**	**31 – 34**
**Characterization of THβCs (11k)**	**35 – 37**
**Characterization of THβCs (11l)**	**38 – 40**
**Characterization of THβCs (11m)**	**41 – 43**
**Characterization of THβCs (11n)**	**44 – 46**
**Characterization of THβCs (11o)**	**47 – 49**
**Characterization of THβCs (11p)**	**50 – 52**
**Characterization of THβCs (11q)**	**53 – 55**
**Characterization of THβCs (11r)**	**56 – 58**
**Characterization of THβCs (11s)**	**59 – 61**
**Characterization of THβCs (11t)**	**62 – 64**
**Characterization of THβCs (11ua/11ub)**	**65 – 67**
**Characterization of THβCs (11wa)**	**68 – 74**
**Characterization of THβCs (11wb)**	**75 – 79**
**References**	**80**

Materials: All reagents and solvents were purchased from commercial suppliers and used without further purification, unless mentioned otherwise. For thin-layer chromatography (TLC) analysis, Merck pre-coated TLC plates (silica gel 60 GF254 0.25mm) were used, and products were observed under UV light or stained in an iodine chamber or phosphomolybdic acid solution. ^{1}H- and ^{13}C-NMR spectra were recorded on a Varian Mercury 400 (400 MHz or 100 MHz, respectively). Chemical shifts are quoted in ppm and are referenced to residual protons in the deuterated solvent as the internal standard, such as $CDCl_3$ (7.26 ppm for ^{1}H and 77.16 ppm for ^{13}C), dimethyl sulfoxide-d6 (DMSO-d_6, 2.50 ppm for ^{1}H and 39.5 ppm for ^{13}C) or CD_3OD (3.34 ppm for ^{1}H and 49.4 ppm for ^{13}C). Coupling constants J are reported in hertz (Hz). Splitting patterns are designated as follows: s, singlet; d, doublet; t, triplet; q, quartet; m, multiplet. IR spectra were recorded with a Perkin-Elmer FT-IR spectrometer, Spectrum Two UATR, and only the characteristic peaks are quoted. Mass spectra were recorded on a Agilent Technologies 6850 gas chromatograph with a mass selective detector Agilent 5973, utilizing electron ionization (EI) at an ionizing energy of 70 eV. A fused silica column (30 m ×0.25 mm HP-5; cross- linked 5 %, PhMe siloxane, 0.10 μm film thickness) was used with a helium carrier flow of 30 ml/min. The temperature of the column was kept at 3 min delay. The run started at 65 ◦C and ended at 300 ◦C with a slope of 15 ◦C min^{-1}. ESI/APCI low-resolution mass spectra were recorded with an Agilent 1100 MSD ion-trap mass spectrometer equipped with a standard ESI/APCI source. Nitrogen served both as the nebulizer gas and the dry gas. Microwave irradiations were performed by means of a Biotage Initiator, and microanalyses were performed with an EA1108 CHNS-D Fisons Instrument.

General method for the synthesis of THβCs:

Starting material (1.0 mmol, 1.0 eq.) is dissolved in ACN (0.25 M, 4 mL) in a microwave vial, then aldehyde (1.2 mmol, 1.2 eq.) and GO (15 mg/mmol) are added and the mixture is heated at 120°C in a microwave reactor until the starting material is consumed. TLC monitors the reaction (eluent: $CHCl_3$:CH_3OH = 95:5) and GC. Once that tryptamine is consumed, the mixture is filtered through a pad of Celite and washed with fresh ACN. The solvent is evaporated by rotavapor, and the crude so obtained is dissolved, forming a solution of 0.25 M (with respect to imine intermediate) in ACN. The Amberlyst15 (500 mg/mmol with respect to tryptamine) is added and stirred until no more spots, except the aldehyde, are observed in TLC (eluent: $CHCl_3$:CH_3OH = 95:5). Then the mixture is filtered by Gooch and washed with DCM. The Amberlyst15 is separated and washed in a round-bottom flask with 10 mL of a CH_3OH:NH_4OH (8:2) mixture, then stirred for 30 min at room temperature. This mixture is filtered again, and the Amberlyst 15 is washed with fresh DCM using Gooch. Then, the filtrated is portioned between DCM (15 mL) and sat. $NaHCO_3$ (15 mL), the two phases are separated, and the aqueous layer is extracted with fresh DCM (2 x 15 mL). The organic phase is dried over anhydrous Na_2SO_4, filtered, and the solvent is evaporated under reduced pressure. The crude is purified by flash chromatography (gradient elution from 100 $CHCl_3$ to $CHCl_3$:CH_3OH = 95:5) when necessary.

General method for recovering GO: Once the microwave step is complete, the solution is transferred into a Falcon tube, and the vial is washed with 2 × 3 mL of fresh ACN, collecting the GO. The mixture is then centrifuged for 10 minutes at 6000 rpm. The GO is recovered and dried at 60°C for 18 hours

General method for recovering A15: Once Amberlyst15 is filtered through Gooch, it is recovered and stirred for 30 minutes in a 2.0 M HCl solution. Then, the resin is filtered by Gooch and washed with deionized H_2O, EtOH and Acetone and dried in an oven at 50°C for 16h.

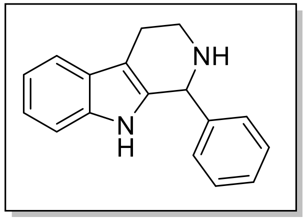

1-phenyl-2,3,4,9-tetrahydro-1H-pyrido[3,4-b]indole 11a. Yield = 87% (0.87 mmol, 215 mg, yellow solid). **M.p. =** 159 – 161°C. **Anal. Calcd.** for $C_{17}H_{16}N_2$ (248.13) C, 82.22; H, 6.49; N, 11.28. **Found**: C, 82.20; H, 6.47; N, 11.25.

GC-MS (EI, 70 eV) = 248 (M^+), 218 (100), 204, 171, 144, 109, 77.

^{1}H-NMR δ (400 MHz, $CDCl_3$, ppm) = 8.10 (s, 1H), 7.60 – 7.56 (m, 1H), 7.36 – 7.33 (m, 3H), 7.27 (ddd, *J* = 5.7, 2.8, 1.5 Hz, 2H), 7.16 – 7.13 (m, 3H), 5.09 (t, *J* = 1.8 Hz, 1H), 3.32 (ddd, *J* = 12.5, 5.2, 4.0 Hz, 1H), 3.15 – 3.06 (m, 1H), 2.93 – 2.79 (m, 2H), 1.84 (s, 1H).

.

^{13}C-NMR δ (101 MHz, $CDCl_3$, ppm) = 141.89, 135.94, 134.52, 128.86, 128.63, 128.24, 127.39, 121.72, 119.37, 118.26, 110.94, 110.19, 58.11, 42.81, 22.58. Spectroscopic data are consistent with those reported in literature.[1]

FT-IR (cm^{-1}) = 3402, 3056, 2917, 2844, 1453, 1298, 1140, 906, 730, 700.

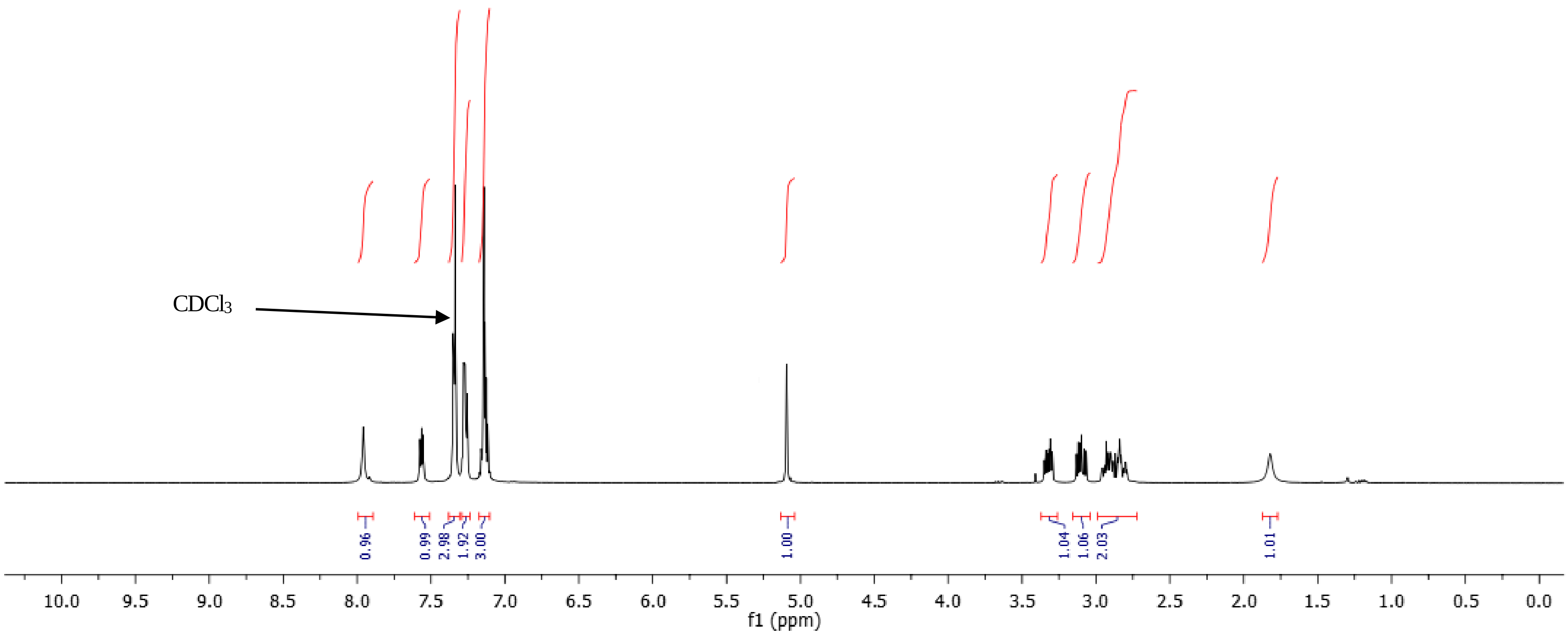

Figure S1. ^{1}H-NMR of compound **11a**

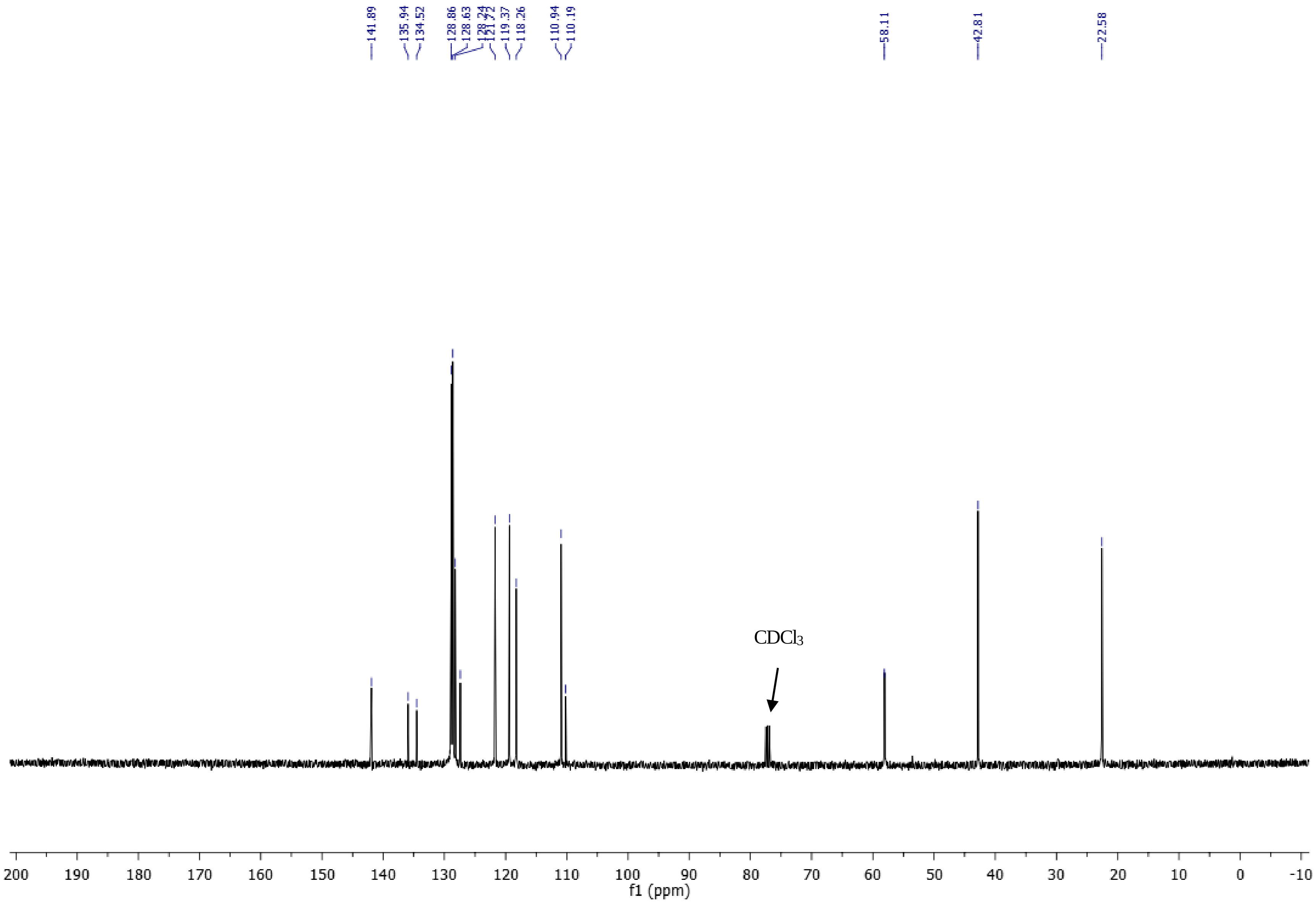

Figure S2. ^{13}C-NMR of compound **11a**

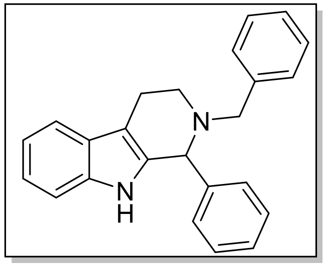

2-benzyl-1-phenyl-2,3,4,9-tetrahydro-1H-pyrido[3,4-b]indole 11b. Yield = 55% (0.55 mmol, 186 mg, yellow solid). **M.p. =** 169-171°C. **Anal. Calcd.** For $C_{24}H_{22}N_2$ (338.45) C, 85.17; H, 6.55; N, 8.28. **Found**: C, 85.19; H, 6.57; N, 8.26.

GC-MS (EI, 70 eV) = 338 ($M^{+\cdot}$), 261, 219 (100), 189, 169, 155, 91.

^{1}H-NMR δ (400 MHz, $CDCl_3$, ppm) = 8.23 – 8.16 (m, 2H), 7.56 (dd, J = 11.7, 5.8 Hz, 3H), 7.48 (dd, J = 5.2, 3.1 Hz, 2H), 7.40 (ddt, J = 8.6, 5.6, 2.2 Hz, 3H), 7.32 (s, 1H, -NH indole), 7.22 – 7.19 (m, 1H), 7.18 – 7.11 (m, 2H), 4.70 (s, 1H), 3.97 (d, J = 14.5 Hz, 1H), 3.51 (d, J = 14.5 Hz, 1H), 3.17 (ddd, J = 11.5, 5.0, 3.5 Hz, 1H), 3.02 – 2.94 (m, 1H), 2.85 (dd, J = 12.4, 2.9 Hz, 1H), 2.76 – 2.68 (m, 1H).

^{13}C-NMR δ (101 MHz, $CDCl_3$, ppm) = 147.84, 147.19, 140.97, 136.45, 134.54, 129.14, 129.09, 129.01, 128.50, 127.16, 123.62, 121.80, 119.59, 118.41, 110.97, 108.88, 65.06, 57.82, 48.92, 21.41. Spectroscopic data are consistent with those reported in literature.[2]

FT-IR (cm^{-1}) = 3052, 2927, 2850, 1450, 1298, 736, 696.

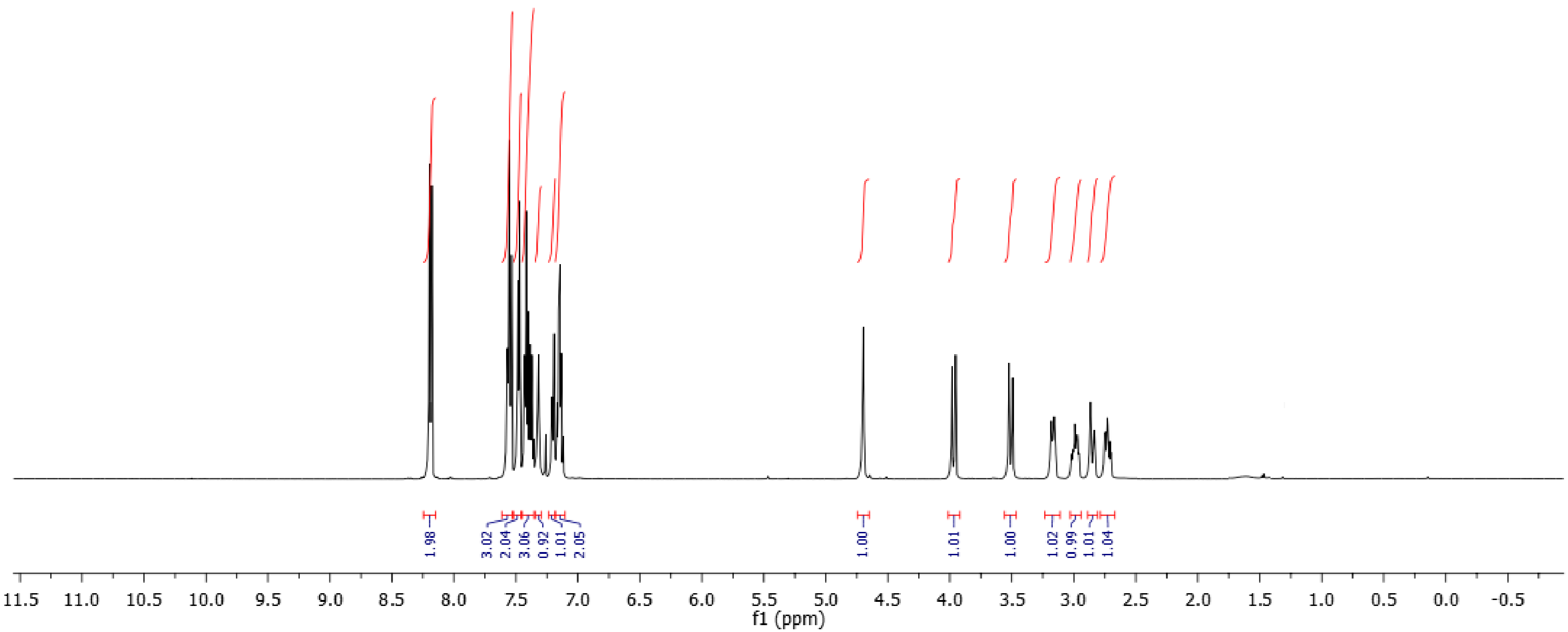

Figure S3. ^{1}H-NMR of compound **11b**

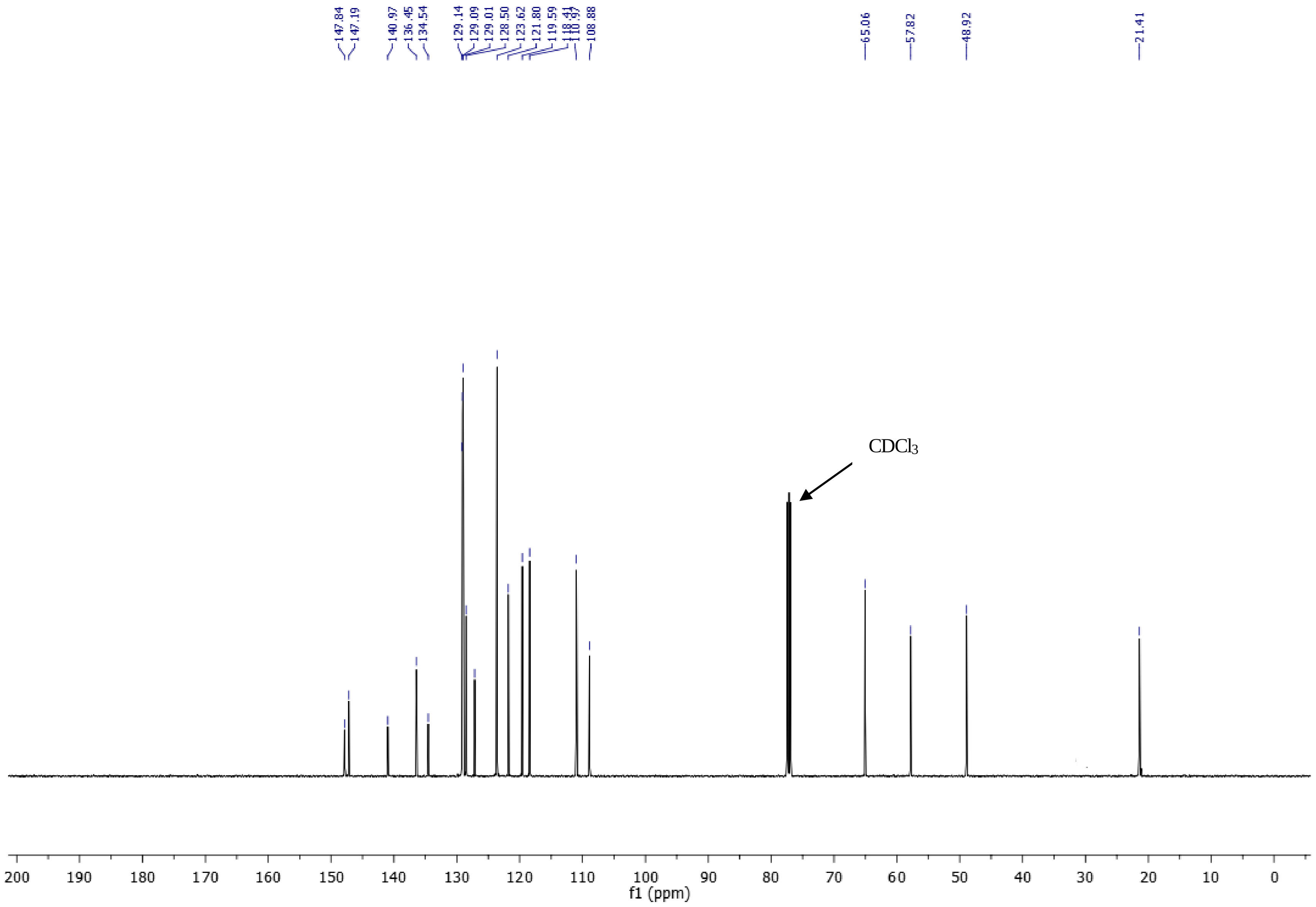

Figure S4. ^{13}C-NMR of compound **11b**

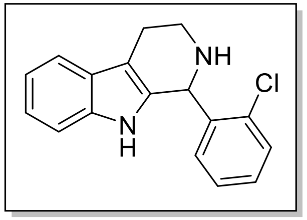

1-(2-chlorophenyl)-2,3,4,9-tetrahydro-1H-pyrido[3,4-b]indole 11c. Yield = 92% (0.92 mmol, 259 mg, pale yellow solid). **M.p. =** 198-201°C. **Anal. Calcd.** for $C_{17}H_{15}ClN_2$ (282.77) C, 72.21; H, 5.35; N, 9.91. **Found**: C, 72.24; H, 5.36; N, 9.93.

GC-MS (EI, 70 eV) = 282 ($M^{+\cdot}$), 253, 218 (100), 191, 171, 144, 108, 75, 51.

^{1}H-NMR δ (500 MHz, $CDCl_3$, ppm) = 7.85 (s, 1H), 7.56 (dd, *J* = 5.9, 2.5 Hz, 1H), 7.46 (dd, *J* = 8.0, 1.1 Hz, 1H), 7.28 – 7.24 (m, 1H), 7.18 – 7.11 (m, 4H), 7.07 (dd, *J* = 7.7, 1.6 Hz, 1H), 5.67 (s, 1H), 3.25 – 3.17 (m, 1H), 3.15 – 3.08 (m, 1H), 2.94 – 2.81 (m, 2H), 2.18 (s, 1H).

^{13}C-NMR δ (126 MHz, $CDCl_3$, ppm) = 139.27, 136.06, 133.98, 133.12, 130.36, 129.98, 129.31, 127.33, 127.13, 121.93, 119.52, 118.34, 111.01, 110.98, 53.75, 41.67, 22.49. Spectroscopic data are consistent with those reported in literature.[1]

FT-IR (cm^{-1}) = 3398, 2922, 2841, 1468, 1446, 1051, 1037, 741.

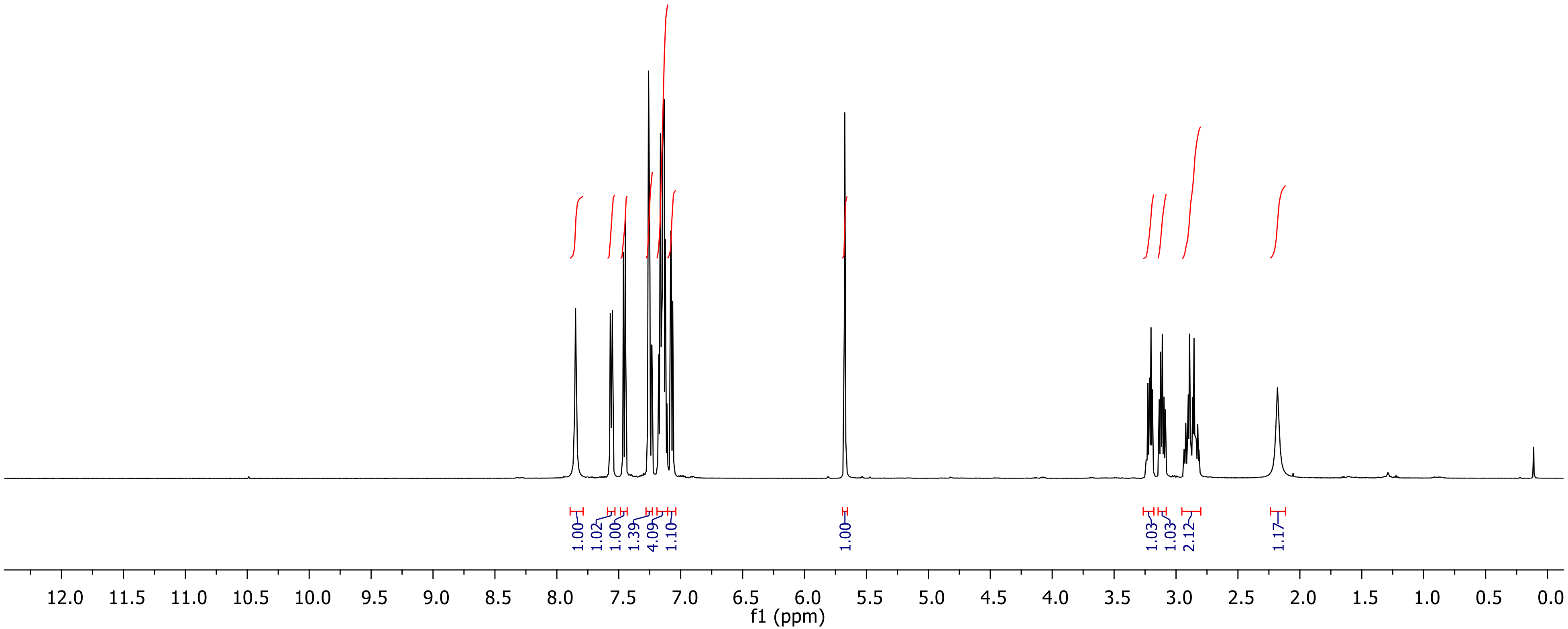

Figure S5. ^{1}H-NMR of compound **11c**

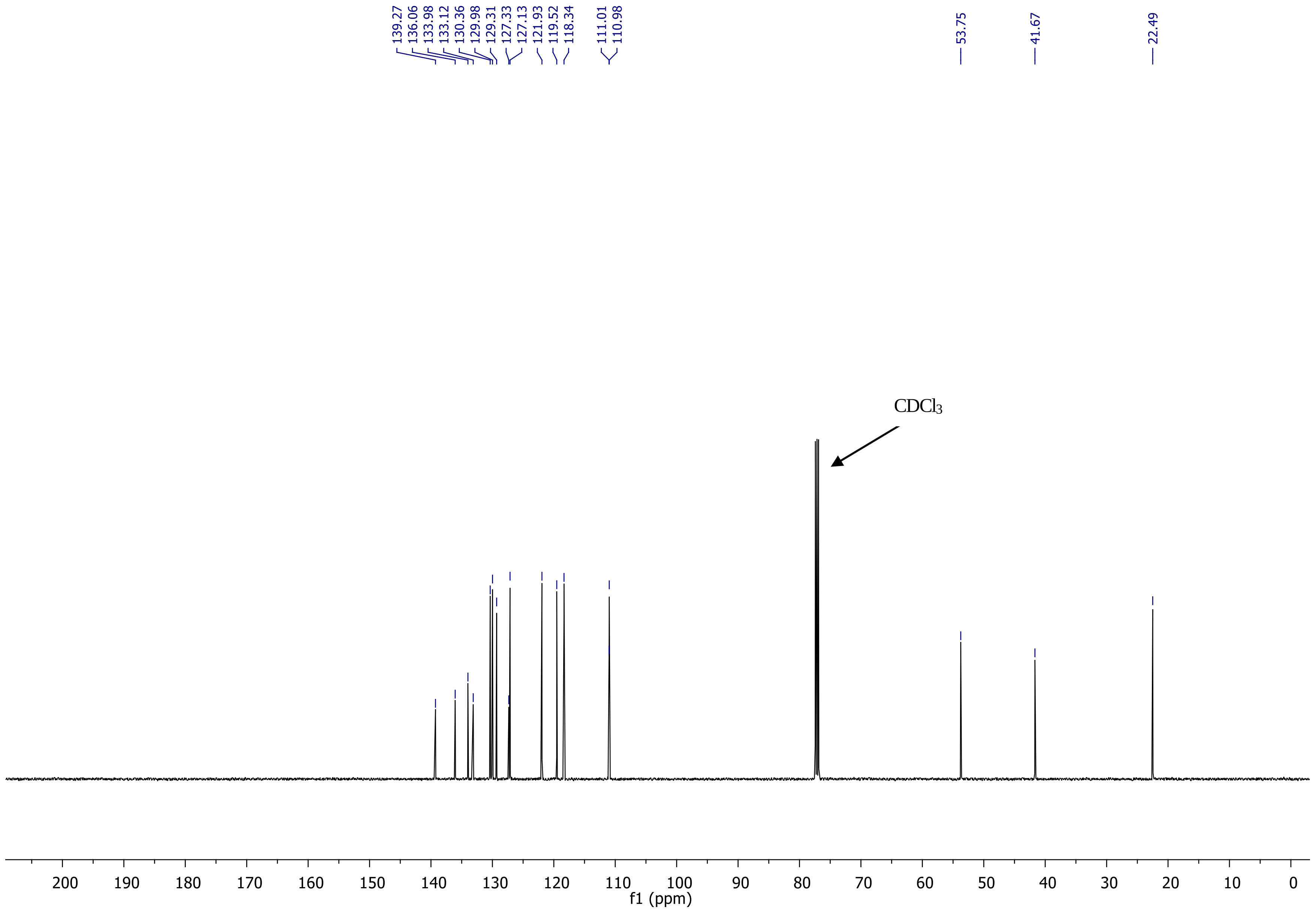

Figure S6. ^{13}C-NMR of compound **11c**

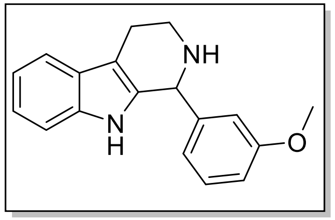

1-(3-methoxyphenyl)-2,3,4,9-tetrahydro-1H-pyrido[3,4-b]indole 11d. Yield = 69% (0.69 mmol, 191 mg, pale yellow solid). **M.p. =** 217–220°C. **Anal. Calcd.** for $C_{18}H_{18}N_2O$ (278.36) C, 77.67; H, 6.52; N, 10.06. **Found**: C, 77.69; H, 6.55; N, 10.05.

GC-MS (EI, 70 eV) = 278 ($M^{+\cdot}$), 249, 218, 191, 171, 144, 115, 96, 77.

^{1}**H-NMR** δ (400 MHz, DMSO-d_6, ppm) = 10.35 (s, 1H), 7.34 (d, *J* = 7.6 Hz, 1H), 7.17 (t, *J* = 7.9 Hz, 2H), 6.96 – 6.85 (m, 2H), 6.80 (dd, *J* = 8.9, 4.8 Hz, 3H), 5.02 (s, 1H), 3.65 (s, *J* = 8.9 Hz, 3H), 3.06 – 2.99 (m, 1H), 2.87 (ddd, *J* = 12.1, 6.8, 5.0 Hz, 1H), 2.72 – 2.56 (m, 2H).

^{13}**C-NMR** δ (101 MHz, DMSO-d_6, ppm) = 159.23, 144.39, 135.97, 135.05, 129.19, 126.83, 120.73, 120.60, 118.25, 117.60, 114.30, 112.62, 111.12, 108.13, 56.63, 55.03, 41.34, 22.07. Spectroscopic data are consistent with those reported in literature.[1]

FT-IR (cm^{-1}) = 3398, 2837, 1603, 1859, 1491, 1450, 1261, 1041, 741.

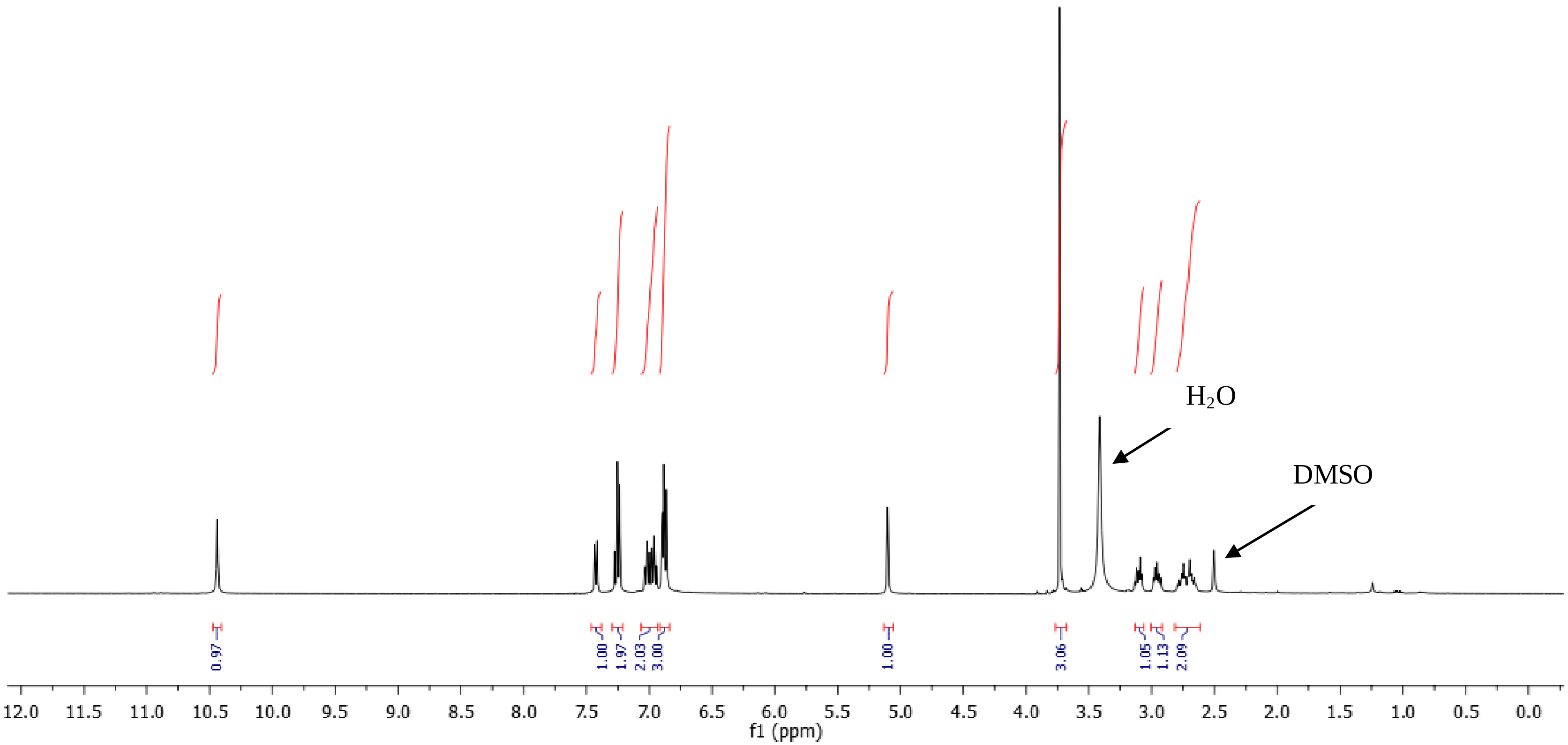

Figure S7. ^{1}H-NMR of compound **11d**

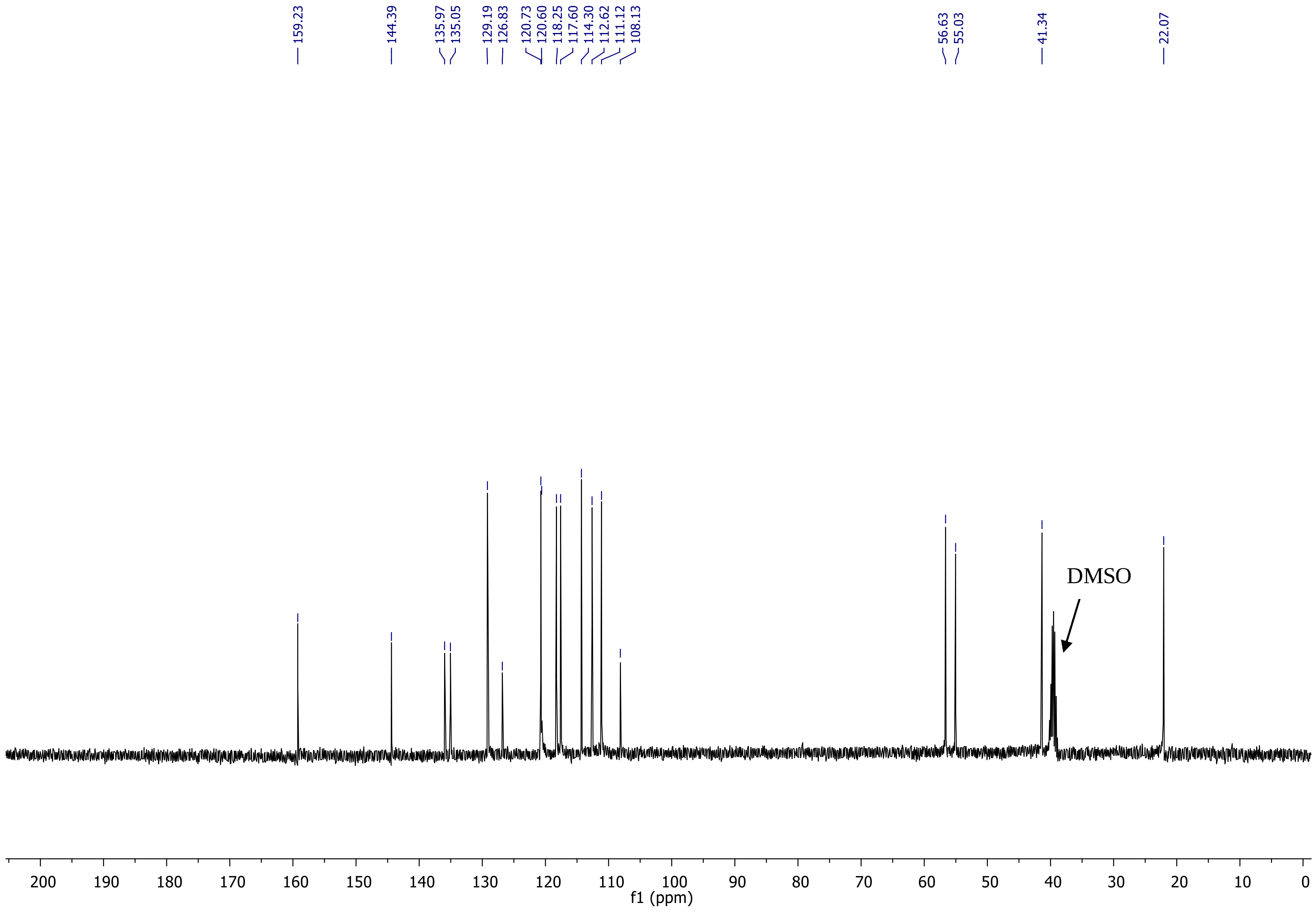

Figure S8. ^{13}C-NMR of compound **11d**

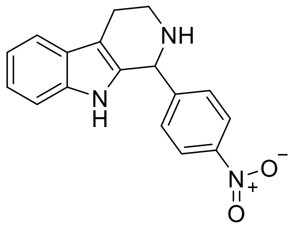

1-(4-nitrophenyl)-2,3,4,9-tetrahydro-1H-pyrido[3,4-b]indole 11e. Yield = 90% (0.90 mmol, 265 mg, yellow solid). **M.p. =** 196-199°C. **Anal. Calcd.** for $C_{17}H_{17}N_3O_2$ (295.34) C, 69.14; H, 5.80; N, 14.23. **Found**: C, 69.16; H, 5.78; N 14.22.

GC-MS (EI, 70 eV) = 293.1($M^{+\cdot}$), 130 (100), 103, 77.

^{1}H-NMR δ (500 MHz, DMSO-d_6, ppm) **=** 10.52 (s, 1H), 8.42 (m, 2H), 7.57 (d, *J* = 8.6 Hz, 2H), 7.44 (d, *J* = 7.7 Hz, 1H), 7.25 (d, *J* = 8.0 Hz, 1H), 7.03 (t, *J* = 7.5 Hz, 1H), 6.97 (t, *J* = 7.4 Hz, 1H), 5.24 (s, 1H), 3.06 – 2.94 (m, 2H), 2.72 (dtd, *J* = 20.0, 14.9, 5.2 Hz, 2H).

^{13}C-NMR δ (126 MHz, DMSO-d_6, ppm) = 151.05, 146.72, 136.05, 134.17, 129.71, 126.79, 123.26, 120.82, 118.37, 117.71, 111.10, 108.66, 55.77, 40.98, 22.08. Spectroscopic data are consistent with those reported in literature.[1]

FT-IR (cm^{-1}) = 3407, 3232, 2850, 1598, 1517, 1450, 1347, 1298, 1091, 1010, 857, 736.

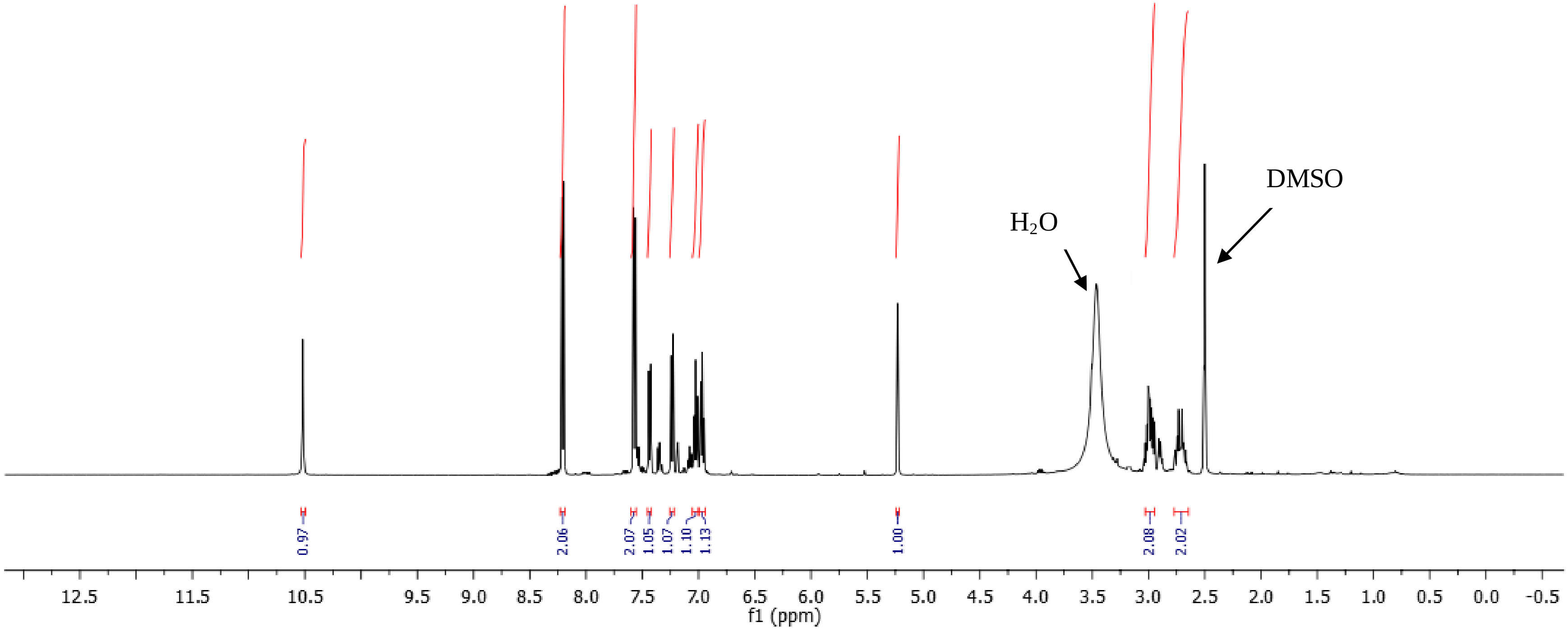

Figure S9. ^{1}H-NMR of compound **11e**

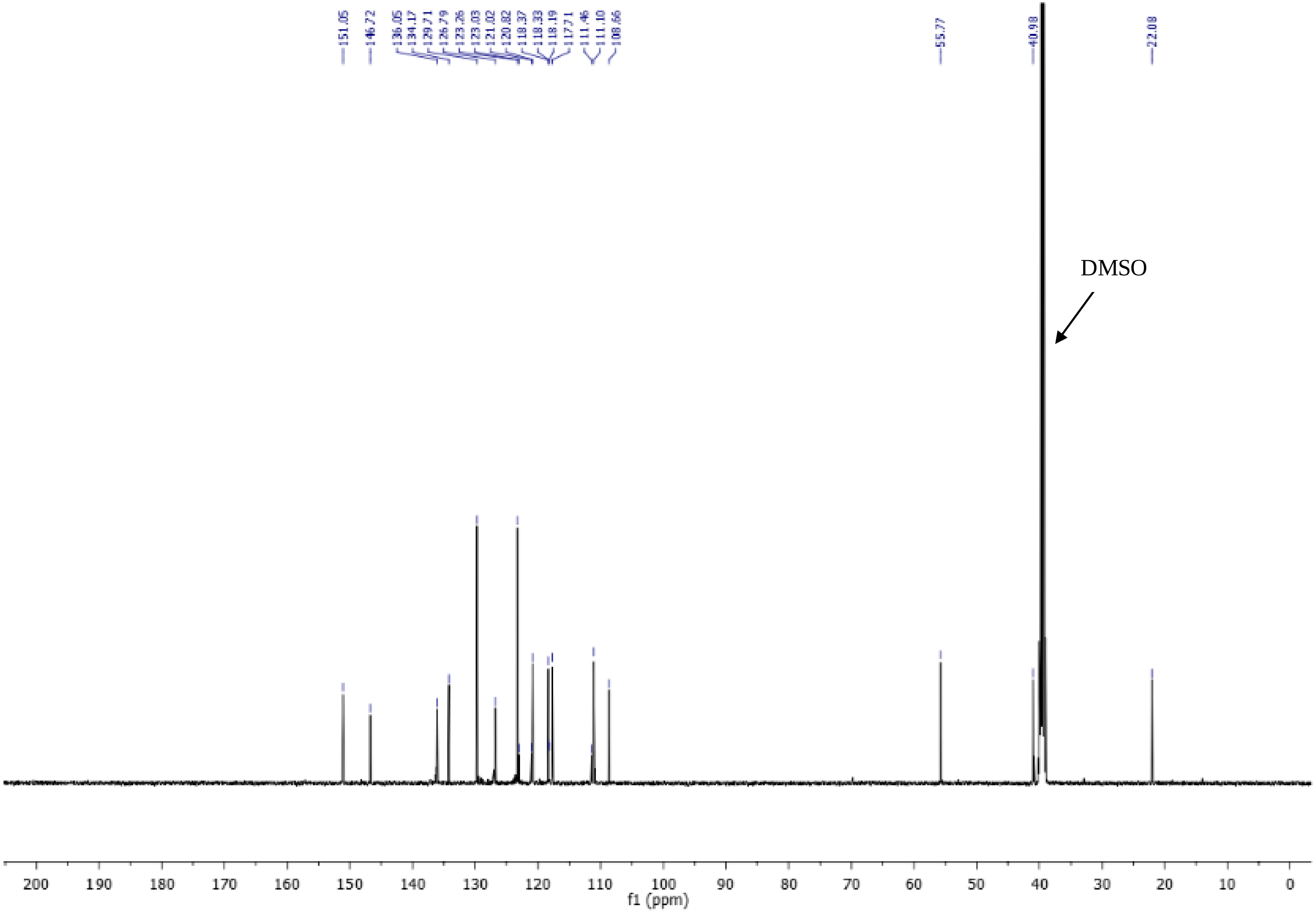

Figure S10. ^{13}C-NMR of compound **11e**.

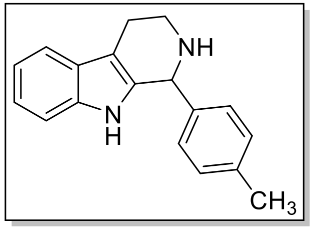

1-(4-methylphenyl)-2,3,4,9-tetrahydro-1H-pyrido[3,4-b]indole 11f. Yield = 59% (0.59 mmol, 155 mg, pale yellow solid). **M.p. =** 156-158°C. **Anal. Calcd.** for $C_{18}H_{18}N_2$ (262.36) C, 82.41; H, 6.92; N, 10.68. **Found**: C, 82.38; H, 6.90; N, 10.69.

GC-MS (EI, 70 eV) = 262 ($M^{+\cdot}$, 100), 232, 218, 171, 144, 127, 109.

^{1}H-NMR δ (400 MHz, $CDCl_3$, ppm) = 7.88 (s, 1H), 7.59 – 7.52 (m, 1H), 7.14 (m, 7H), 5.10 (s, 1H), 3.33 (m, 1H), 3.15 – 3.07 (m, 1H), 2.98 – 2.91 (m, 1H), 2.89 - 2.78 (m,1H), 2.37 (s, 3H), 2.29 (s, 1H).

^{13}C-NMR δ (101 MHz, $CDCl_3$, ppm) = 138.73, 138.04, 135.96, 134.66, 129.53, 128.55, 127.44, 121.72, 119.39, 118.27, 110.94, 110.09, 57.78, 42.79, 22.53, 21.26. Spectroscopic data are consistent with those reported in literature.[1]

FT-IR (cm^{-1}) = 3398, 3954, 2921, 1512, 1444, 1092, 1011, 801, 734, 493.

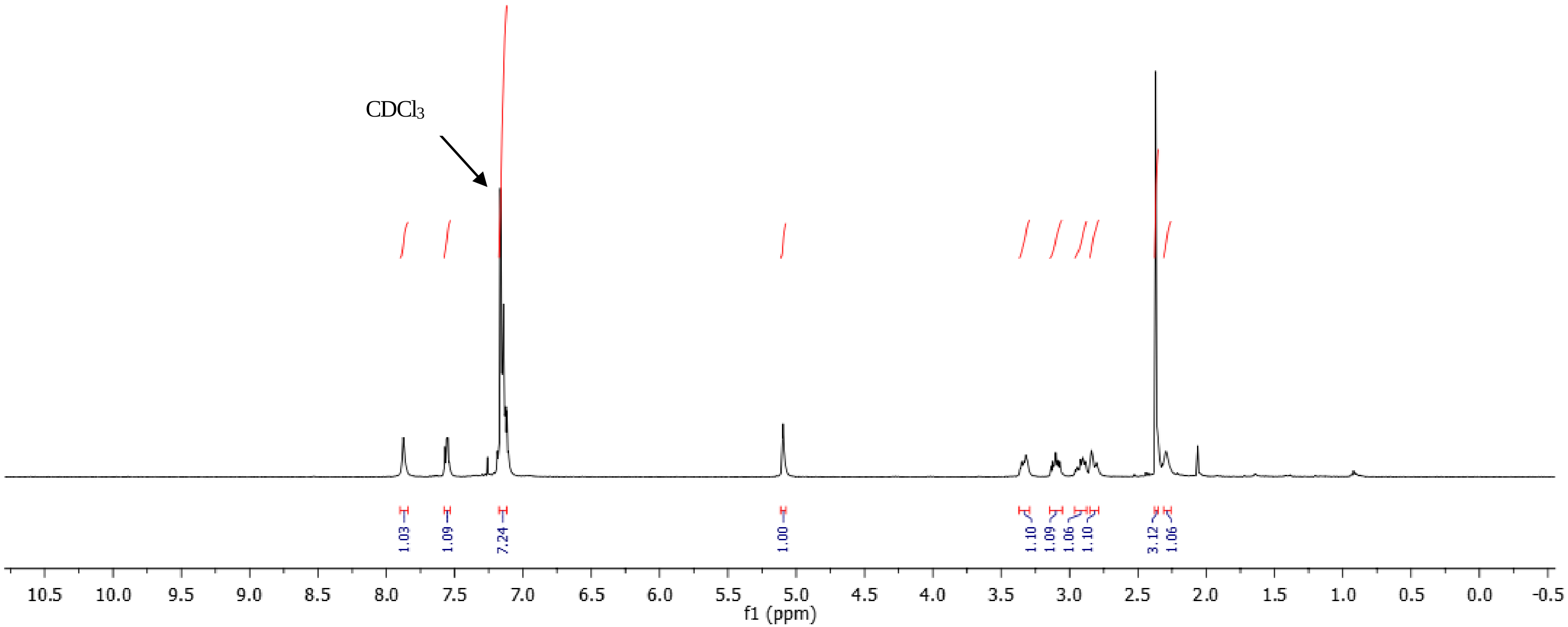

Figure S11. ^{1}H-NMR of compound **11f**

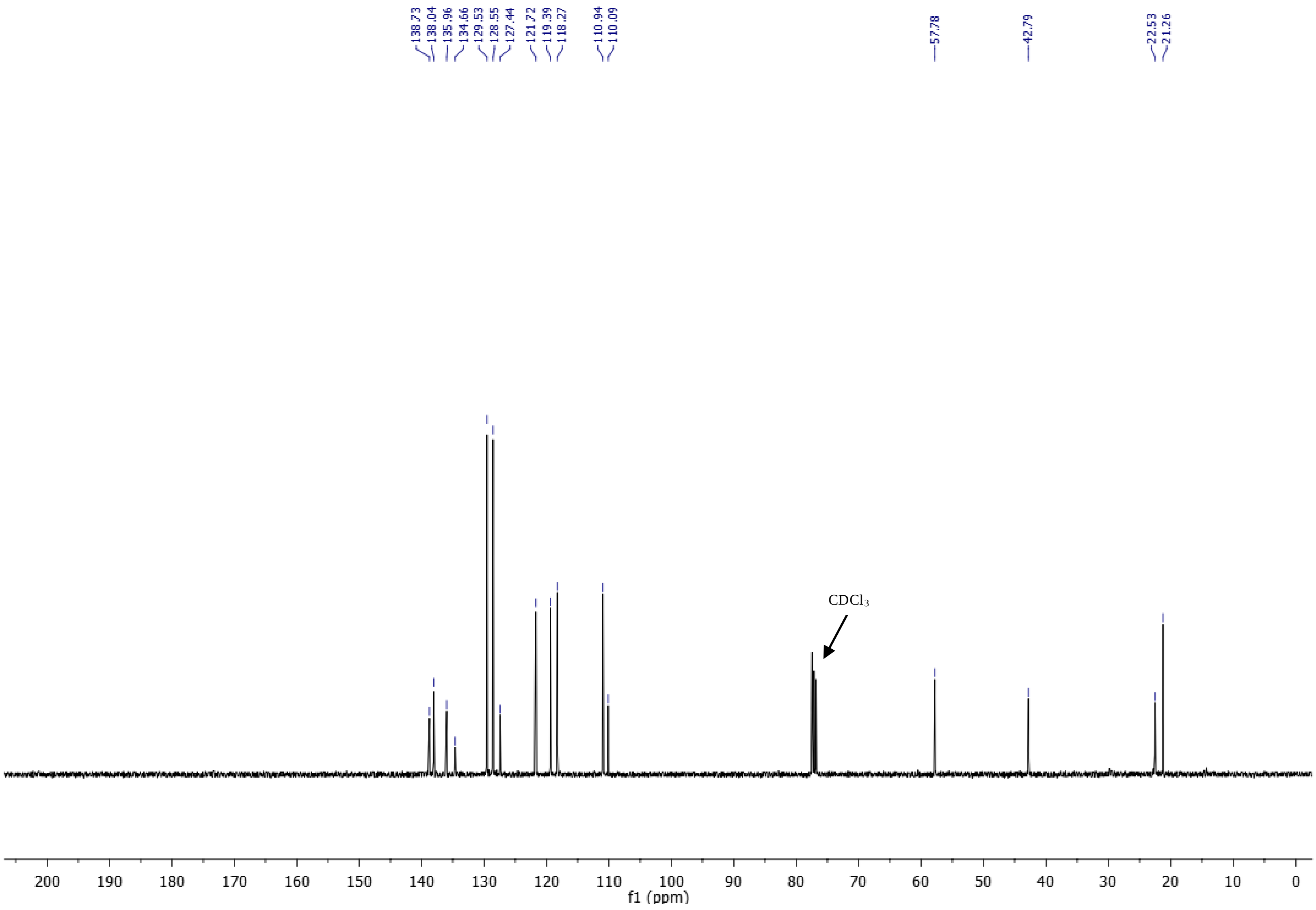

Figure S12. ^{13}C-NMR of compound **11f**

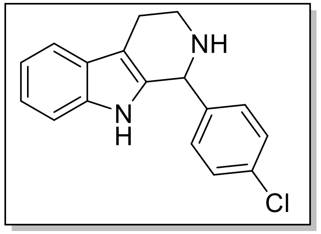

1-(4-chlorophenyl)-2,3,4,9-tetrahydro-1H-pyrido[3,4-b]indole 11g. Yield = 60% (0.60 mmol, 169 mg, white solid). **M.p. =** 201 – 203°C. **Anal. Calcd.** For $C_{17}H_{15}ClN_2$ (282.77) C, 72.21; H, 5.35; N, 9.91. **Found**: C, 72.24; H, 5.37; N 9.87.

GC-MS (EI, 70 eV) = 282 ($M^{+\cdot}$), 253, 218 (100), 189, 171, 144, 108, 75, 51**.**

^{1}H-NMR δ (400 MHz, $CDCl_3$, ppm) **=** 7.65 (s, 1H), 7.58 (d, *J* = 7.0 Hz, 1H), 7.38 – 7.33 (m, 2H), 7.29 – 7.22 (m, 3H), 7.20 – 7.13 (m, 2H), 5.15 (s, 1H), 3.40 – 3.33 (m, 1H), 3.20 – 3.11 (m, 1H), 3.00 – 2.91 (m, 1H), 2.85 (ddd, *J* = 11.1, 4.0, 2.0 Hz, 1H), 2.04 (s, 1H).

^{13}C-NMR δ (101 MHz, $CDCl_3$, ppm) = 140.48, 136.06, 134.14, 133.96, 130.00, 129.09, 127.46, 122.06, 119.66, 118.44, 110.99, 110.57, 57.49, 42.77, 22.58. Spectroscopic data are consistent with those reported in literature.[1]

FT-IR (cm^{-1}) = 3394, 3053, 2927, 2850, 1486, 1450, 1086, 1015, 821, 741.

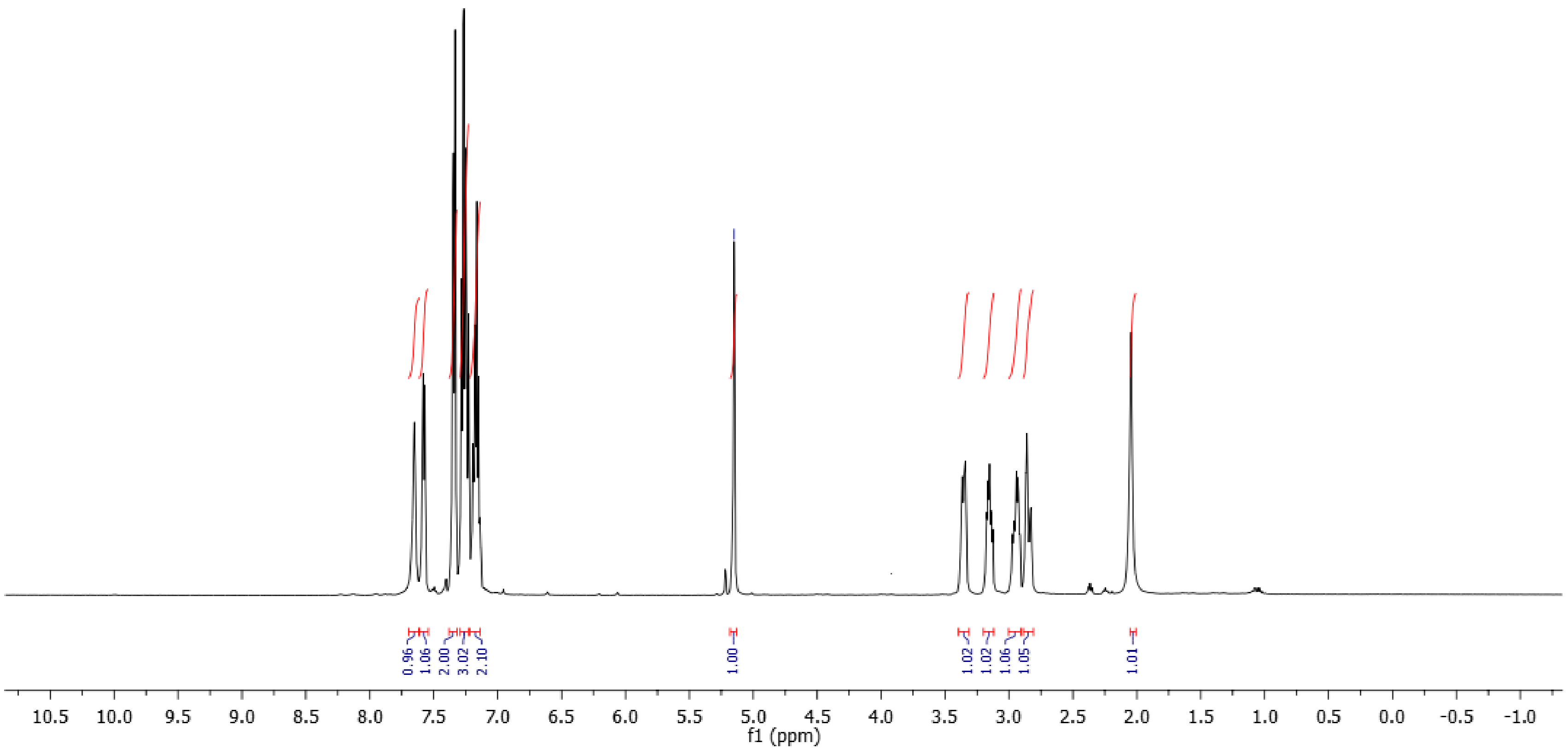

Figure S13. ^{1}H-NMR of compound **11g**

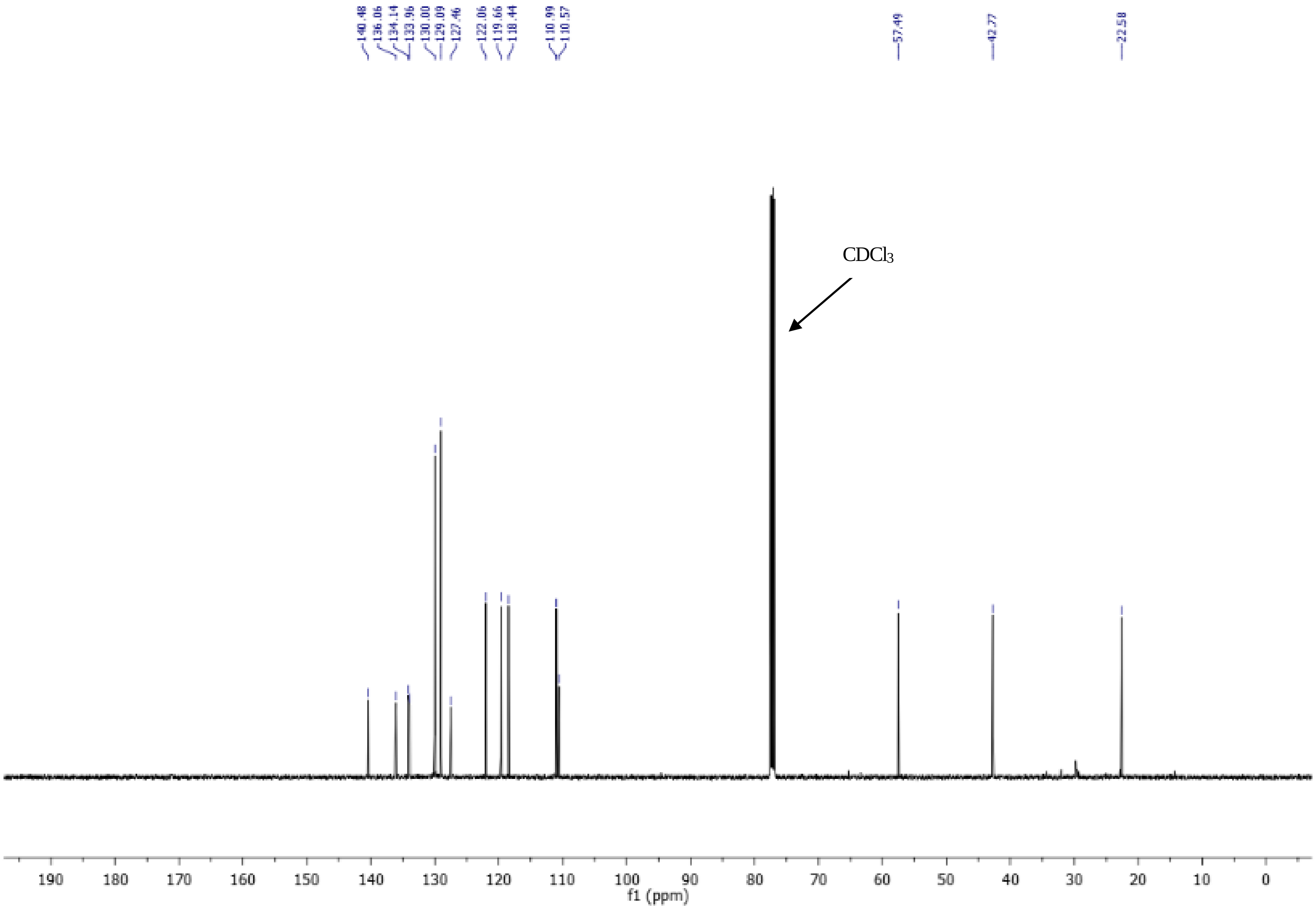

Figure S14. ^{13}C-NMR of compound **11g**

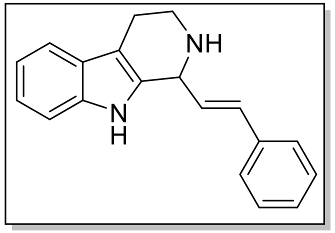

1-[(E)-phenylvinyl]-2,3,4,9-tetrahydro-1H-pyrido[3,4-b]indole 11h. Yield = 58% (0.58 mmol, 159 mg, orange solid). **M.p. =** 218-221°C. **Anal. Calcd.** For $C_{19}H_{18}N_2$ (274.37) C, 83.18; H, 6.61; N, 10.21. **Found**: C, 83.14; H, 6.58; N, 10.20.

GC-MS (EI, 70 eV) = 274 ($M^{+\cdot}$), 244, 197, 171, 154, 128, 91, 65.

^{1}H-NMR δ (400 MHz, $CDCl_3$, ppm) = 7.92 (s, 1H), 7.53 (d, *J* = 7.5 Hz, 1H), 7.43 – 7.38 (m, 2H), 7.35 – 7.25 (m, 4H), 7.18 – 7.09 (m, 2H), 6.68 (d, *J* = 15.8 Hz, 1H), 6.35 (dd, *J* = 15.8, 8.1 Hz, 1H), 4.76 (d, *J* = 8.0 Hz, 1H), 3.38 (dt, *J* = 12.3, 4.9 Hz, 1H), 3.13 (ddd, *J* = 12.7, 8.1, 5.0 Hz, 1H), 2.87 – 2.76 (m, 2H), 2.25 (s, 1H).

^{13}C-NMR δ (101 MHz, $CDCl_3$, ppm) =136.35, 135.89, 133.81, 133.16, 129.51, 128.82, 128.17, 127.69, 126.70, 121.83, 119.50, 118.37, 110.96, 109.29, 56.06, 42.42, 22.48. Spectroscopic data are consistent with those reported in literature.[2]

FT-IR (cm^{-1}) = 3398, 3295, 3052, 2922, 1719, 1612, 1446, 1302, 974, 741, 691.

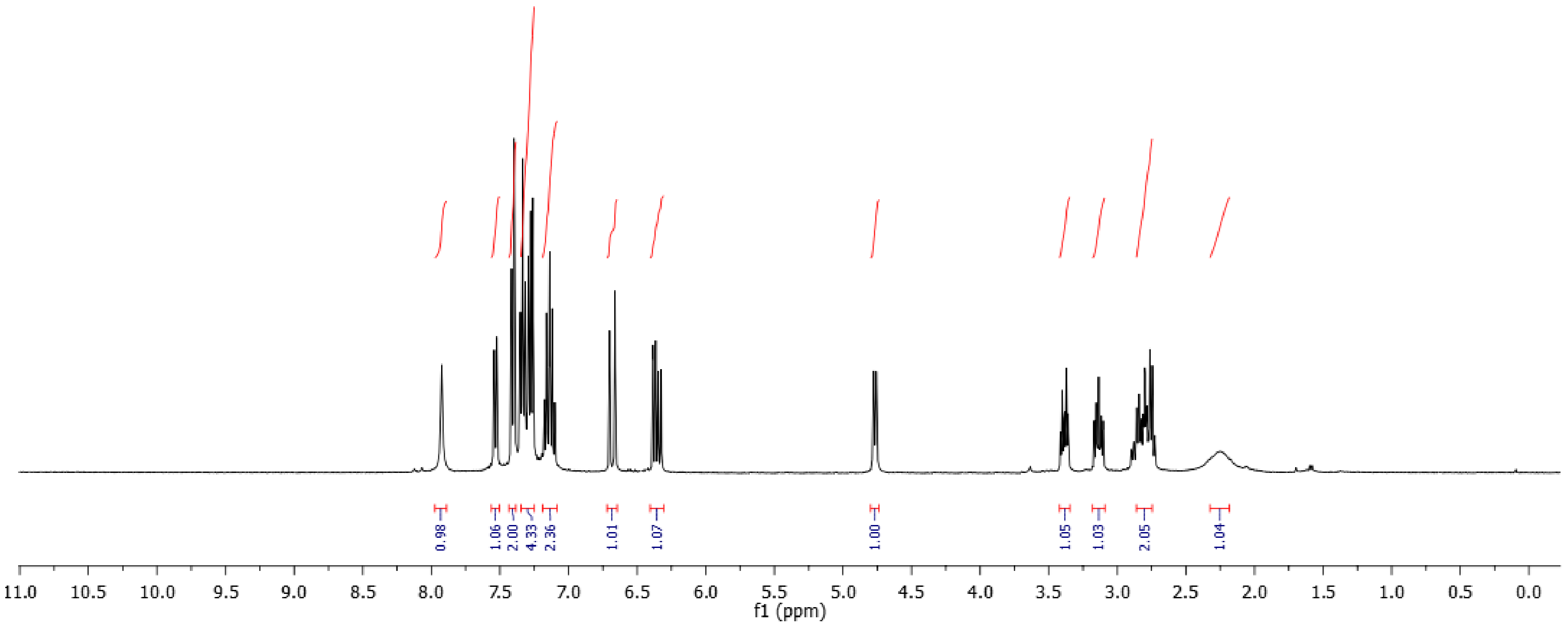

Figure S15. ¹H-NMR of compound **11h**

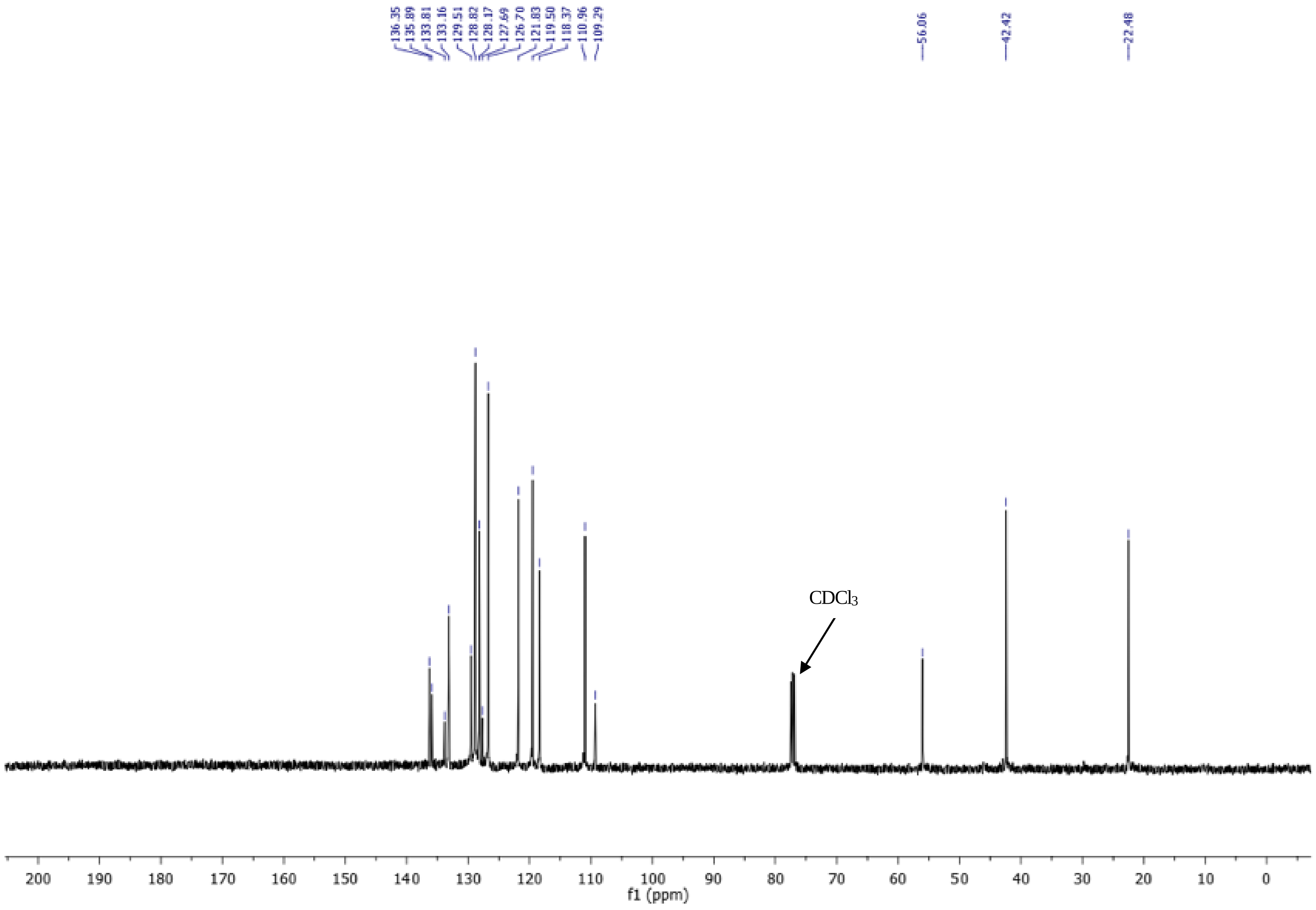

Figure S16. ^{13}C-NMR of compound **11h**

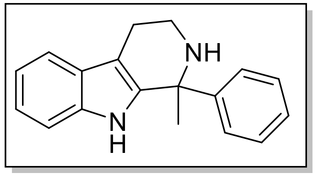

1-methyl-1-phenyl-2,3,4,9-tetrahydro-1H-pyrido[3,4-b]indole 11i. Yield = 33% (0.33 mmol, 86 mg, light yellow solid). **M.p. =** 155-159°C. **Anal. Calcd.** For $C_{18}H_{18}N_2$ (262.36) C, 82.41; H, 6.92; N, 10.68. **Found**: C, 82.38; H, 6.90; N 10.69.

GC-MS (EI, 70 eV) = 262 ($M^{+\cdot}$), 247 (100), 232, 217, 185, 144, 115, 77.

^{1}H-NMR δ (400 MHz, $CDCl_3$, ppm) = 7.79 (s, 1H), 7.56 (d, *J* = 7.6 Hz, 1H), 7.34 – 7.27 (m, 5H), 7.25 – 7.22 (m, 1H), 7.21 – 7.17 (m, 1H), 7.16 – 7.11 (m, 1H), 3.18 – 3.11 (m, 1H), 2.92 – 2.87 (m, 2H, including -NH), 2.82 (dd, *J* = 8.3, 5.3 Hz, 1H), 2.78 – 2.72 (m, 1H), 1.85 (s, 3H).

^{13}C-NMR δ (101 MHz, $CDCl_3$, ppm) = 146.34, 138.31, 135.84, 128.38, 127.49, 127.35, 126.95, 121.96, 119.60, 118.55, 110.98, 109.84, 56.98, 46.03, 39.85, 22.89. Spectroscopic data are consistent with those reported in literature.[3]

FT-IR (cm^{-1}) = 3398, 3208, 3052, 2927, 1450, 1298, 911, 736, 696.

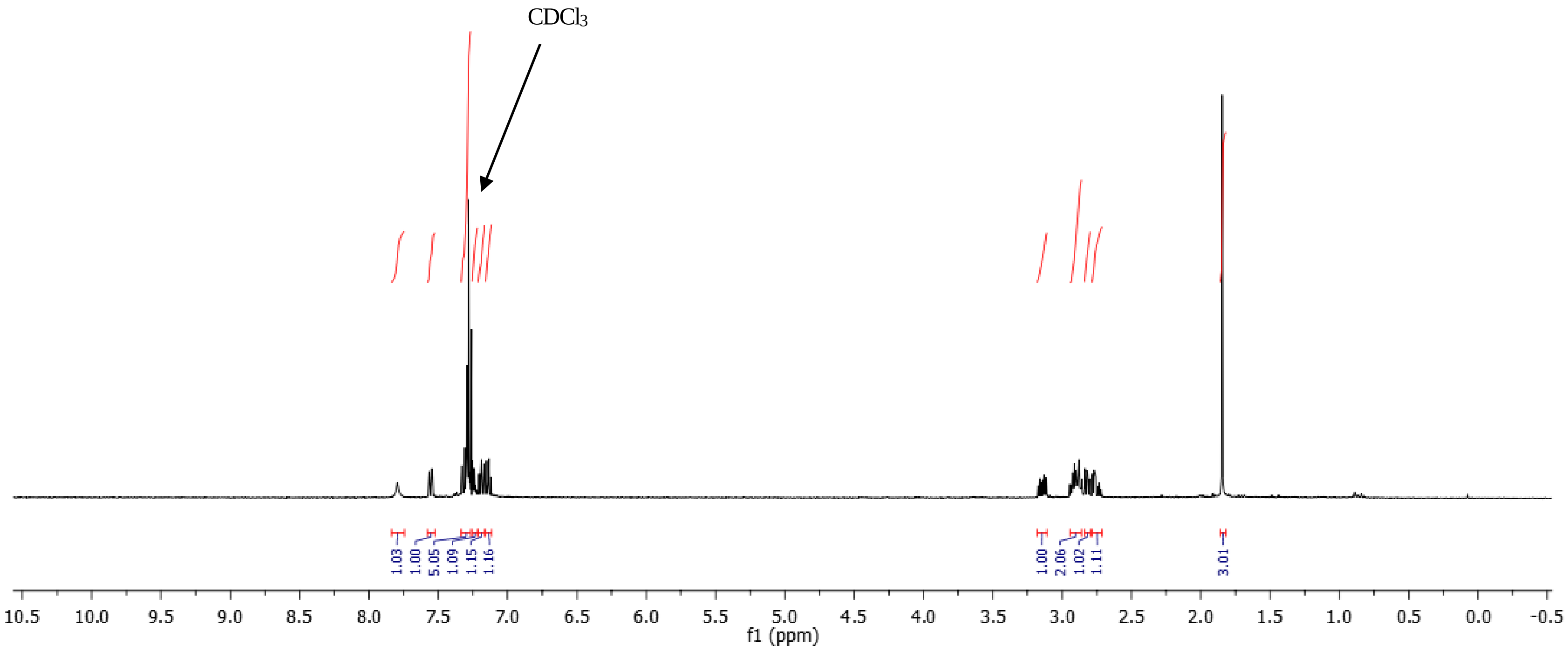

Figure S17. ^{1}H-NMR of compound **11i**

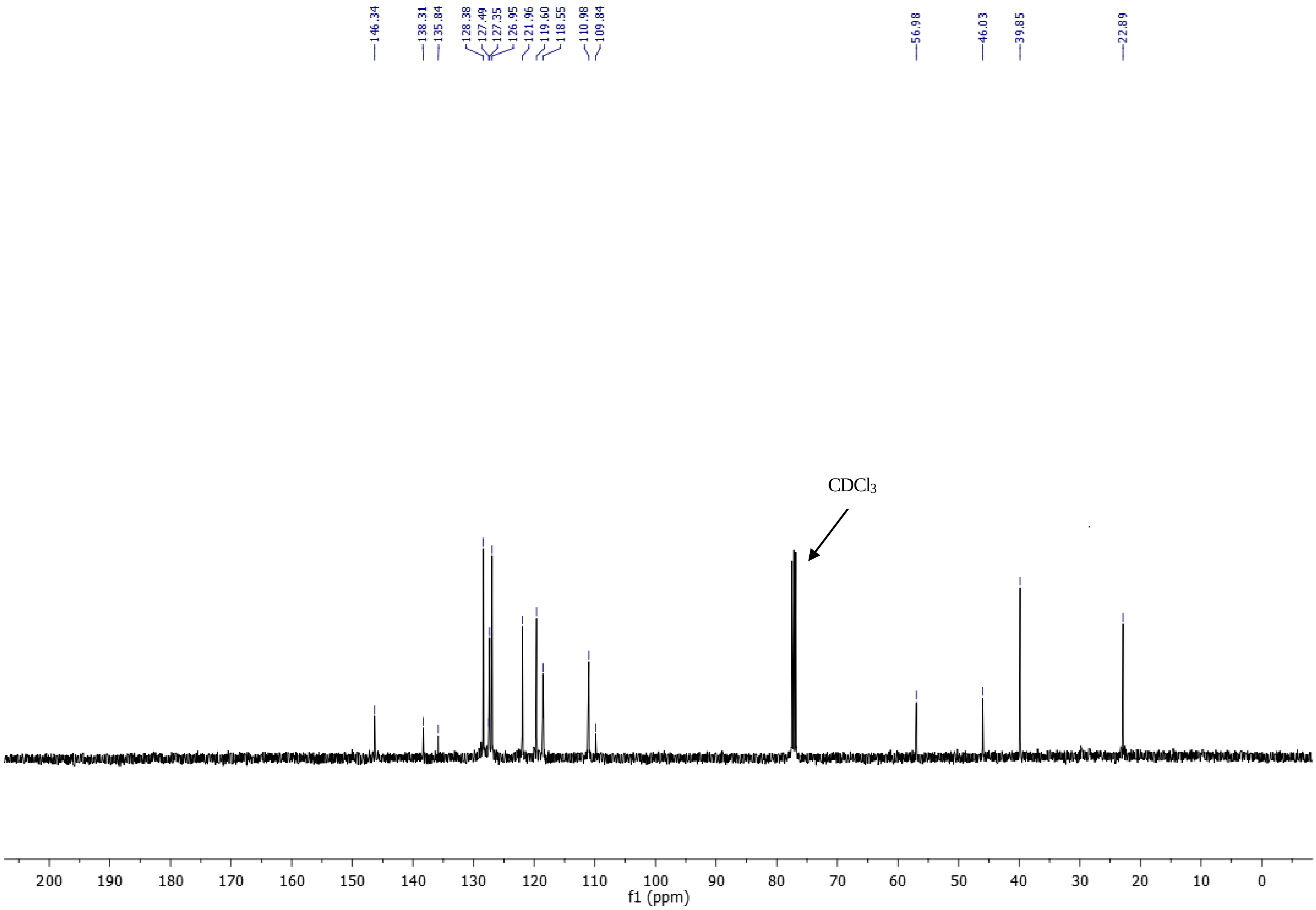

Figure S18. ^{13}C-NMR of compound **11i**

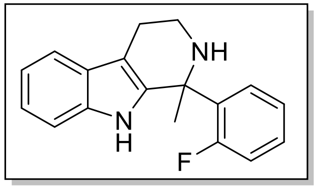

1-(2-fluorophenyl)-1-methyl-2,3,4,9-tetrahydro-1H-pyrido[3,4-b]indole 11j. Yield =35% (0.35 mmol, 98 mg, white solid). **M.p.** = 201-203°C. **Anal. Calcd.** For $C_{18}H_{17}FN_2$ (280.35) C, 77.12; H, 6.11; N 9.99. **Found**: C, 77.13; H, 6.07; N 9.96.

GC-MS (EI, 70 eV) = 280 ($M^{+\cdot}$), 150, 130 (100), 109, 77.

^{1}H-NMR δ (400 MHz, $CDCl_3$, ppm) =8.01 (s, 1H), 7.57 – 7.53 (m, 1H), 7.37 – 7.33 (m, 1H), 7.25 – 7.04 (m, 4H), 6.98 – 6.93 (m, 1H), 6.90 – 6.83 (m, 1H), 3.27 – 3.15 (m, 1H), 2.94 – 2.83 (m, 2H), 2.78 – 2.67 (m, 1H), 2.48 (s, 1H), 1.95 (d, *J* = 1.7 Hz, 3H).

^{13}C-NMR δ (101 MHz, $CDCl_3$, ppm) = 162.07, 159.63, 137.06, 135.80, 132.86, 132.75, 130.62, 130.57, 129.34, 129.25, 127.35, 123.79, 123.76, 122.11, 119.64, 118.70, 116.83, 116.59, 111.03, 109.87, 56.90, 56.88, 40.19, 27.45, 27.40, 22.48.

^{19}F – NMR (471 MHz, $CDCl_3$) = -111.41

FT-IR (cm^{-1}) = 3406, 3064, 2924, 2844, 1579, 1442, 1219, 1123, 742.

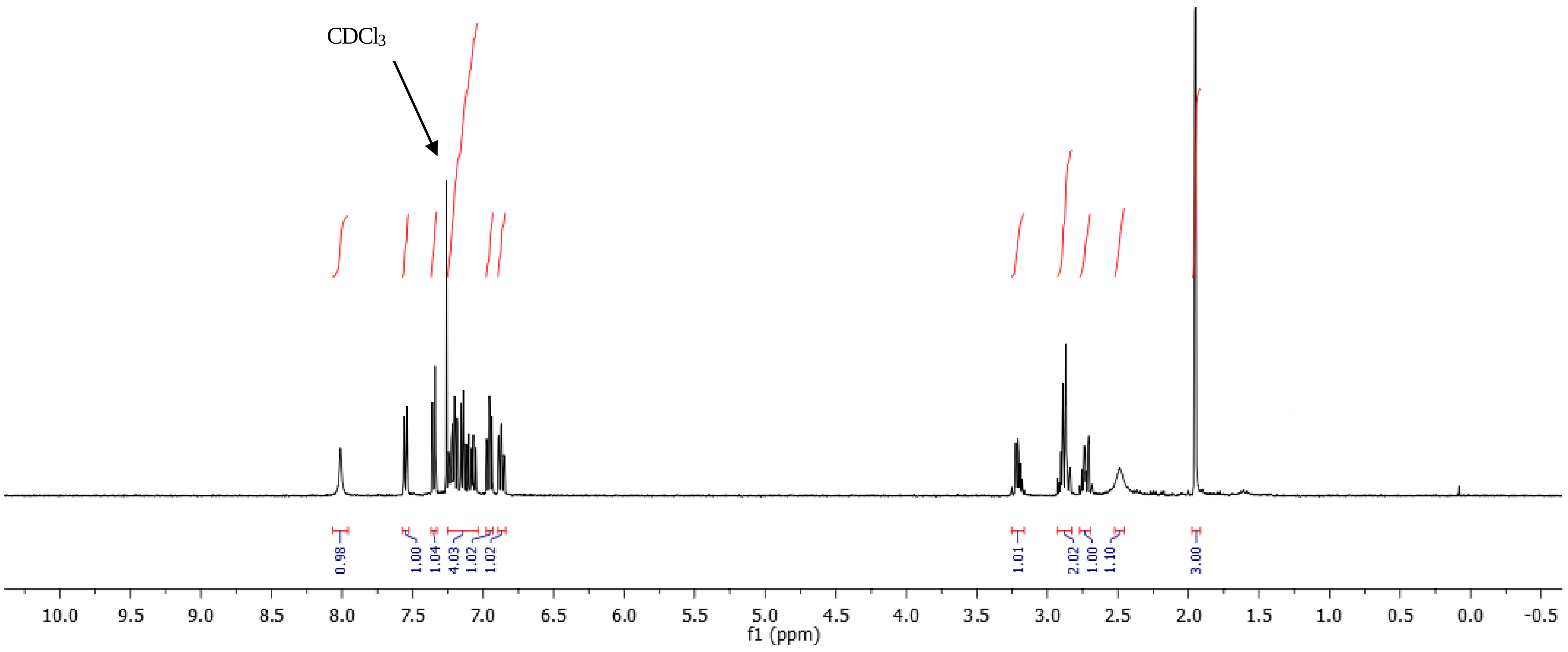

Figure S19. ^{1}H-NMR of compound **11j**

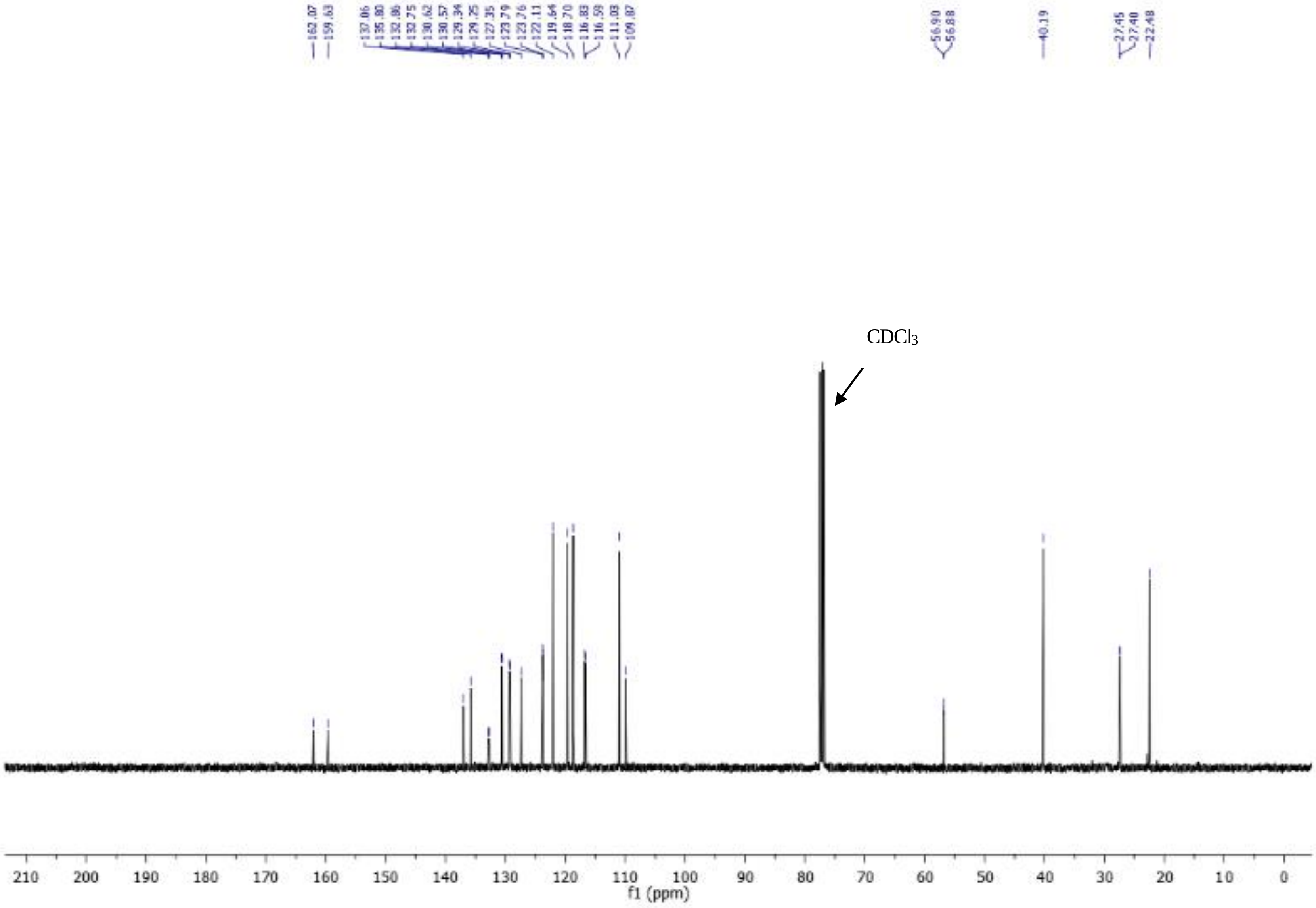

Figure S20. ^{13}C-NMR of compound **11j**

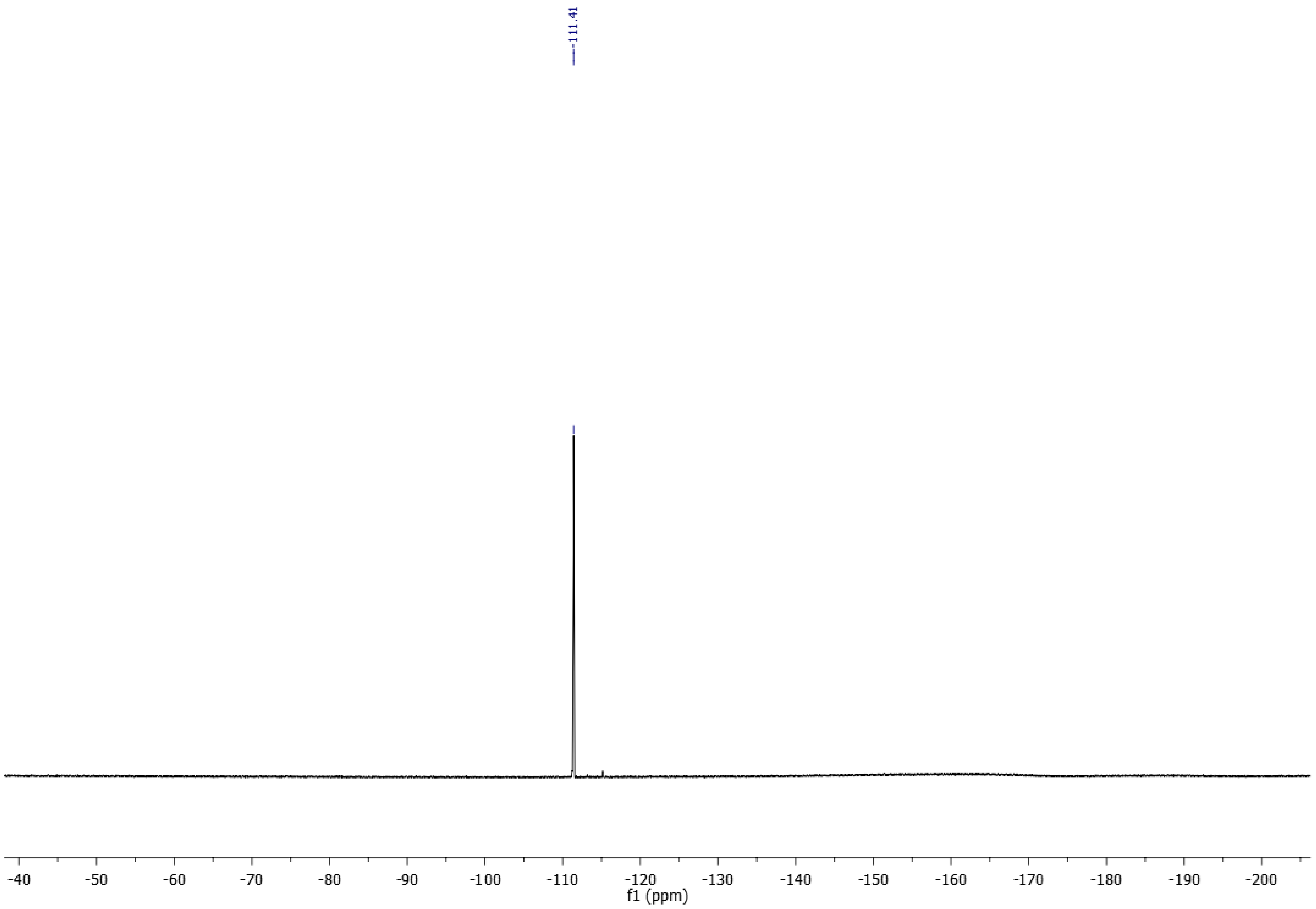

Figure S21. ^{19}F-NMR of compound **11j**

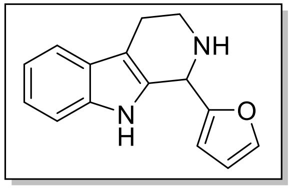

1-(furan-2-yl)-2,3,4,9-tetrahydro-1H-pyrido[3,4-b]indole 11k. Yield = 88% (0.88 mmol, 209 mg, dark brown solid). **M.p. =** 138-142°C. **Anal. Calcd.** For $C_{15}H_{14}N_2O$ (238.29) C, 75.61; H, 5.92; N, 11.76; **Found**: C, 75.64; H, 5.93; N, 11.78.

GC-MS (EI, 70 eV) = 238 ($M^{+\cdot}$), 209, 180, 167, 152, 130, 115, 90, 77.

^{1}H-NMR δ (500 MHz, $CDCl_3$, ppm) = 8.12 (s, 1H), 7.56 (d, *J* = 7.7 Hz, 1H), 7.48 – 7.42 (m, 1H), 7.32 – 7.28 (m, 1H), 7.21 – 7.13 (m, 2H), 6.34 (dd, J = 3.2, 1.9 Hz, 1H), 6.18 (dd, J = 3.2, 0.7 Hz, 1H)., 5.30 (s, 1H), 3.33 (dt, *J* = 12.5, 5.6 Hz, 1H), 3.16 (dt, *J* = 6.1, 5.5 Hz, 1H), 2.90 – 2.79 (m, 2H), 2.17 (s, 1H).

^{13}C-NMR δ (126 MHz, $CDCl_3$, ppm) = 154.66, 142.55, 135.90, 132.01, 127.37, 122.00, 119.47, 118.40, 111.01, 110.36, 110.10, 107.58, 50.96, 41.76, 22.47. Spectroscopic data are consistent with those reported in literature.[4]

FT-IR (cm^{-1}) = 3403.4, 3058.4, 2928.5, 2843.3, 1455.2, 1297, 1142.7, 1012.9, 907.33, 598.87, 436.53.

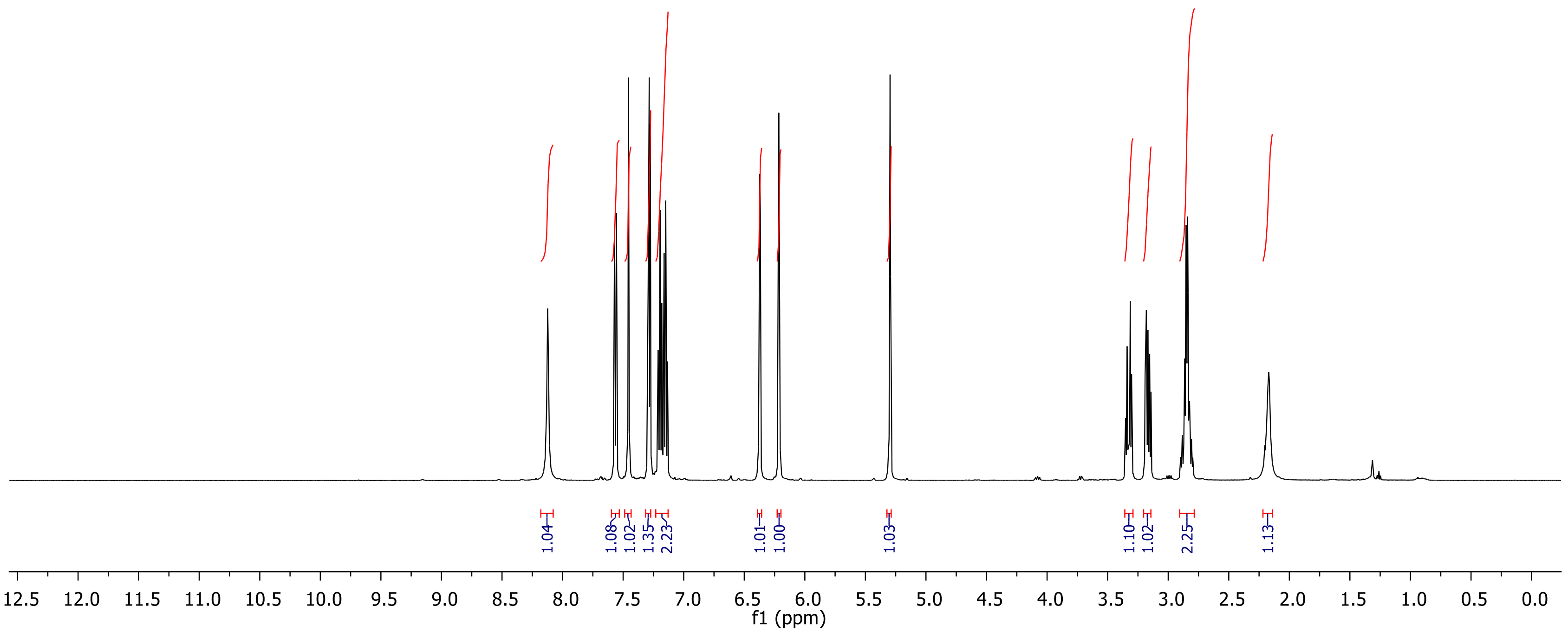

Figure S22. ^{1}H-NMR of compound **11k**

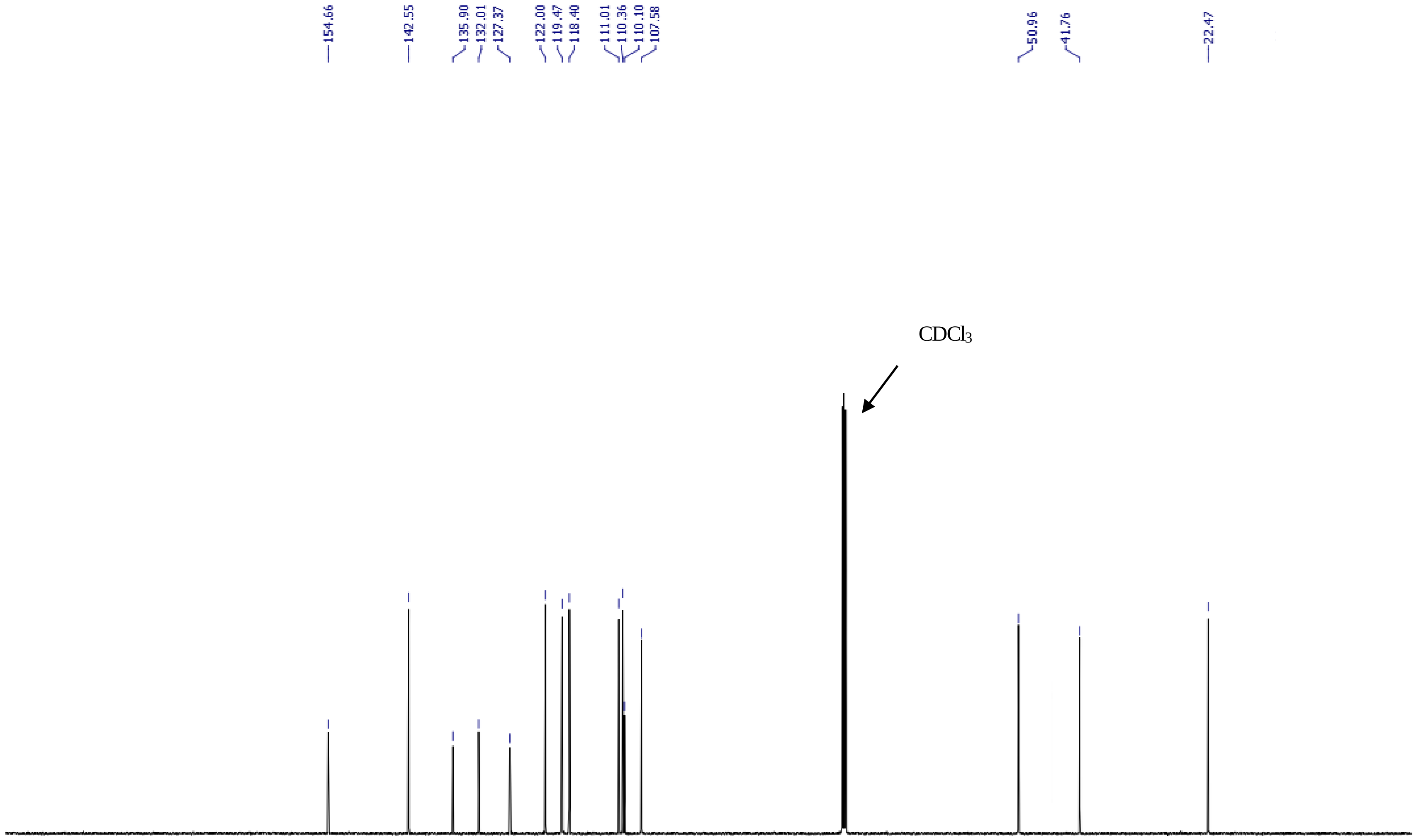

Figure S23. ^{13}C-NMR of compound **11k**

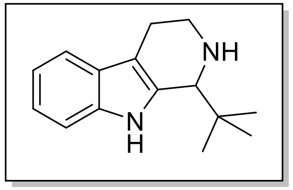

1-(tert-butyl)-2,3,4,9-tetrahydro-1H-pyrido[3,4-b]indole 11l. Yield = 75% (0.75 mmol, 171 mg, white solid). **M.p. =** 175-178°C. **Anal. Calcd.** For $C_{15}H_{20}N_2$ (228.34) C, 78.90; H, 8.83; N, 12.27; **Found**: C, 78.93; H, 8.84; N, 12.29.

GC-MS (EI, 70 eV) = 228 ($M^{+\cdot}$), 171 (100), 156, 144, 115.

^{1}H-NMR δ (400 MHz, $CDCl_3$, ppm) = 7.87 (s, 1H), 7.52 (d, *J* = 7.7 Hz, 1H), 7.34 (d, *J* = 7.7 Hz, 1H), 7.14 (m, 2H), 3.88 (d, *J* = 1.7 Hz, 1H), 3.40 (m, 1H), 2.97 – 2.87 (m, 1H), 2.75 (m, 2H), 2.27 (s, 1H), 1.13 (s, 9H).

^{13}C-NMR δ (101 MHz, $CDCl_3$, ppm) = 135.68, 134.44, 127.21, 121.68, 119.37, 118.04, 111.89, 110.70, 62.52, 43.58, 35.89, 27.53, 23.09. Spectroscopic data are consistent with those reported in literature.[1]

FT-IR (cm^{-1}) = 3445, 2963, 2838, 2875, 1454, 1302, 1229, 1115, 731.

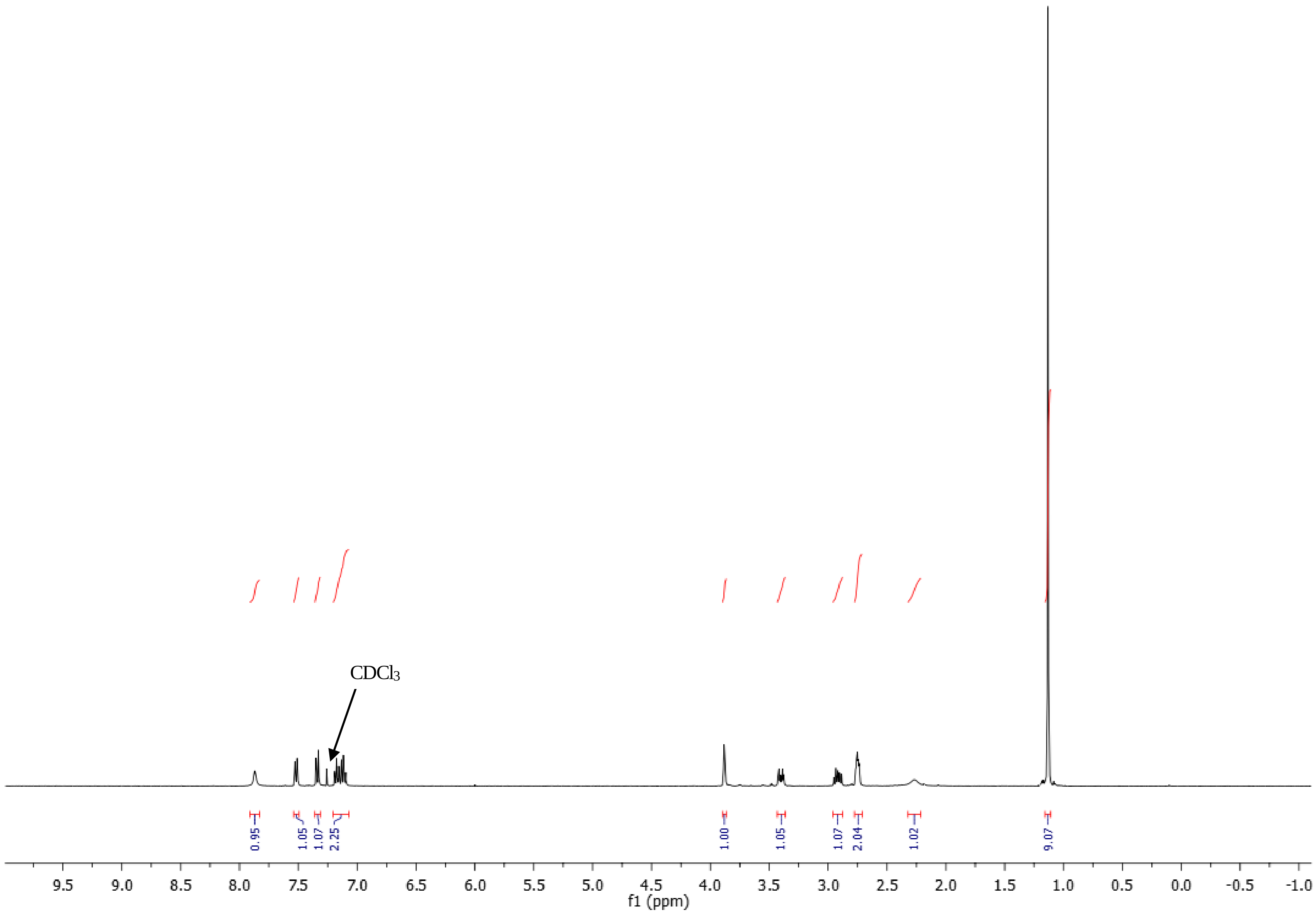

Figure S24. ^{1}H-NMR of compound **11l**

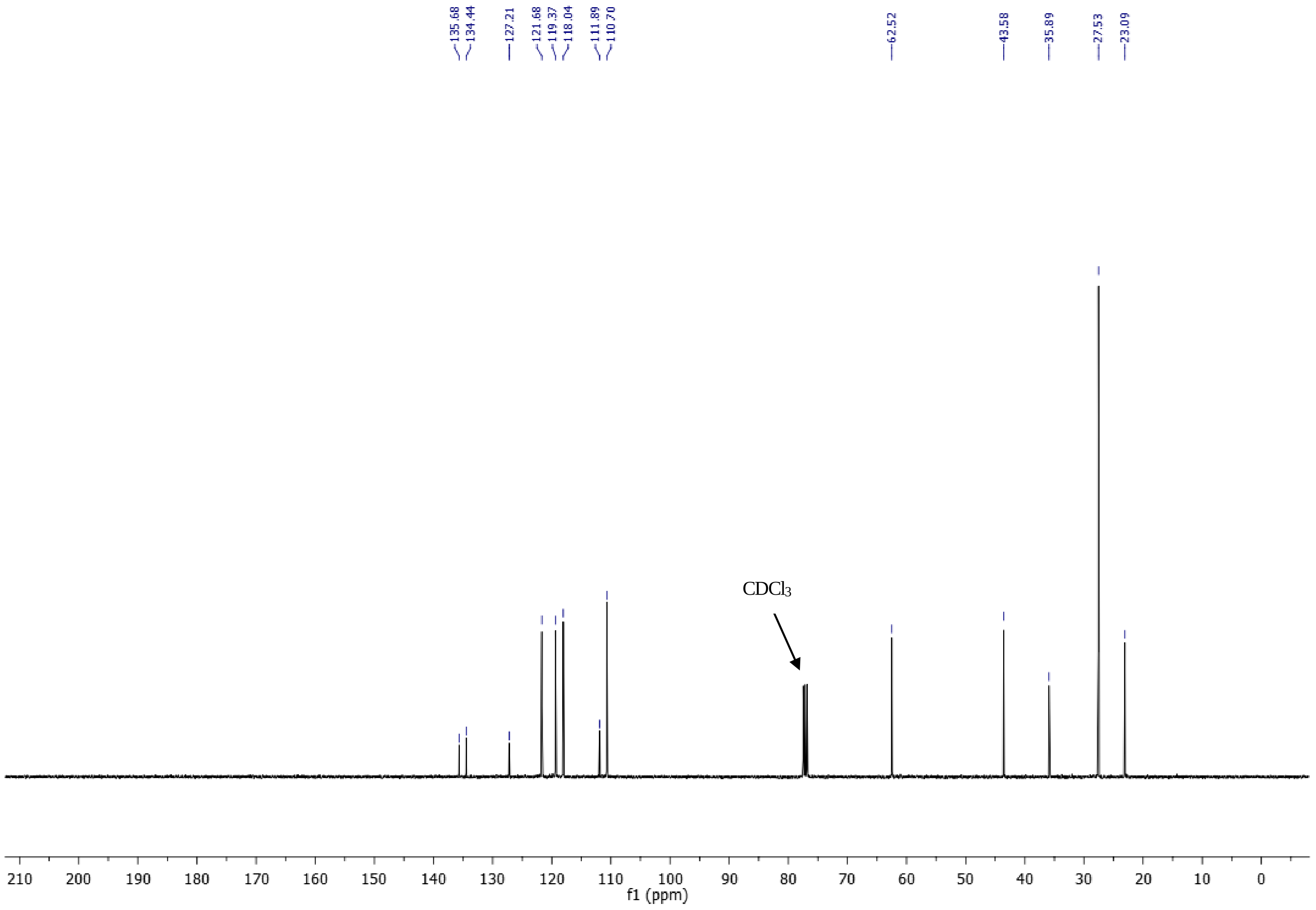

Figure S25. ^{13}C-NMR of compound **11l**

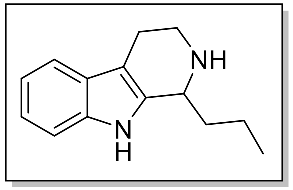

1-propyl-2,3,4,9-tetrahydro-1H-pyrido[3,4-b]indole 11m. Yield = 48% (0.48 mmol, 102 mg, pale-yellow solid). **M.p. =** 186-188°C. **Anal. Calcd.** For $C_{14}H_{18}N_2$ (214.31) C, 78.46; H, 8.47; N, 13.07. **Found**: C, 78.42; H, 8.43; N 13.04.

GC-MS (EI, 70 eV) = 214 ($M^{+\cdot}$), 184, 171 (100), 154, 143, 130, 115, 85.

^{1}H-NMR δ (400 MHz, DMSO-d_6, ppm) = 10.70 (s, 1H), 7.35 (d, *J* = 7.7 Hz, 1H), 7.27 (dt, *J* = 8.0, 0.8 Hz, 1H), 7.03 – 6.97 (m, 1H), 6.93 (ddd, *J* = 8.0, 7.1, 1.1 Hz, 1H), 4.00 (d, *J* = 6.0 Hz, 1H), 3.19 (dt, *J* = 12.4, 4.7 Hz, 1H), 2.92 – 2.84 (m, 1H), 2.69 – 2.56 (m, 2H), 1.89 (dtd, *J* = 13.3, 6.5, 3.4 Hz, 1H), 1.67 – 1.38 (m, 2H), 0.93 (t, *J* = 7.3 Hz, 3H).

^{13}C-NMR δ (101 MHz, DMSO-d_6, ppm) = 136.66, 135.68, 126.97, 120.40, 118.22, 117.40, 110.91, 106.95, 51.90, 41.82, 36.04, 21.91, 18.56, 14.16. Spectroscopic data are consistent with those reported in literature.[5]

FT-IR (cm^{-1}) = 2956, 2925, 2849, 1452, 1319, 1297, 1154, 1122, 1006, 734.

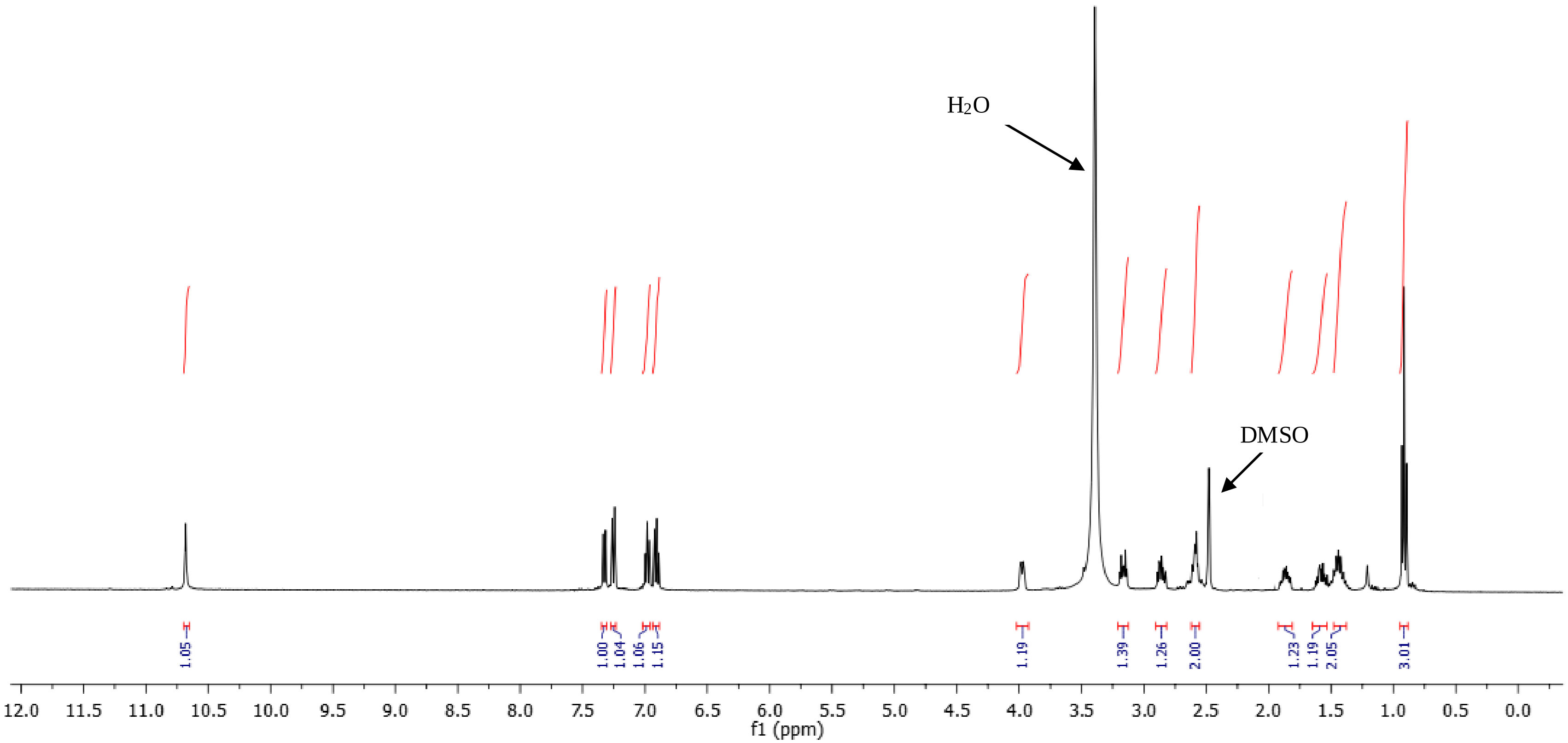

Figure S26. ^{1}H-NMR of compound **11m**

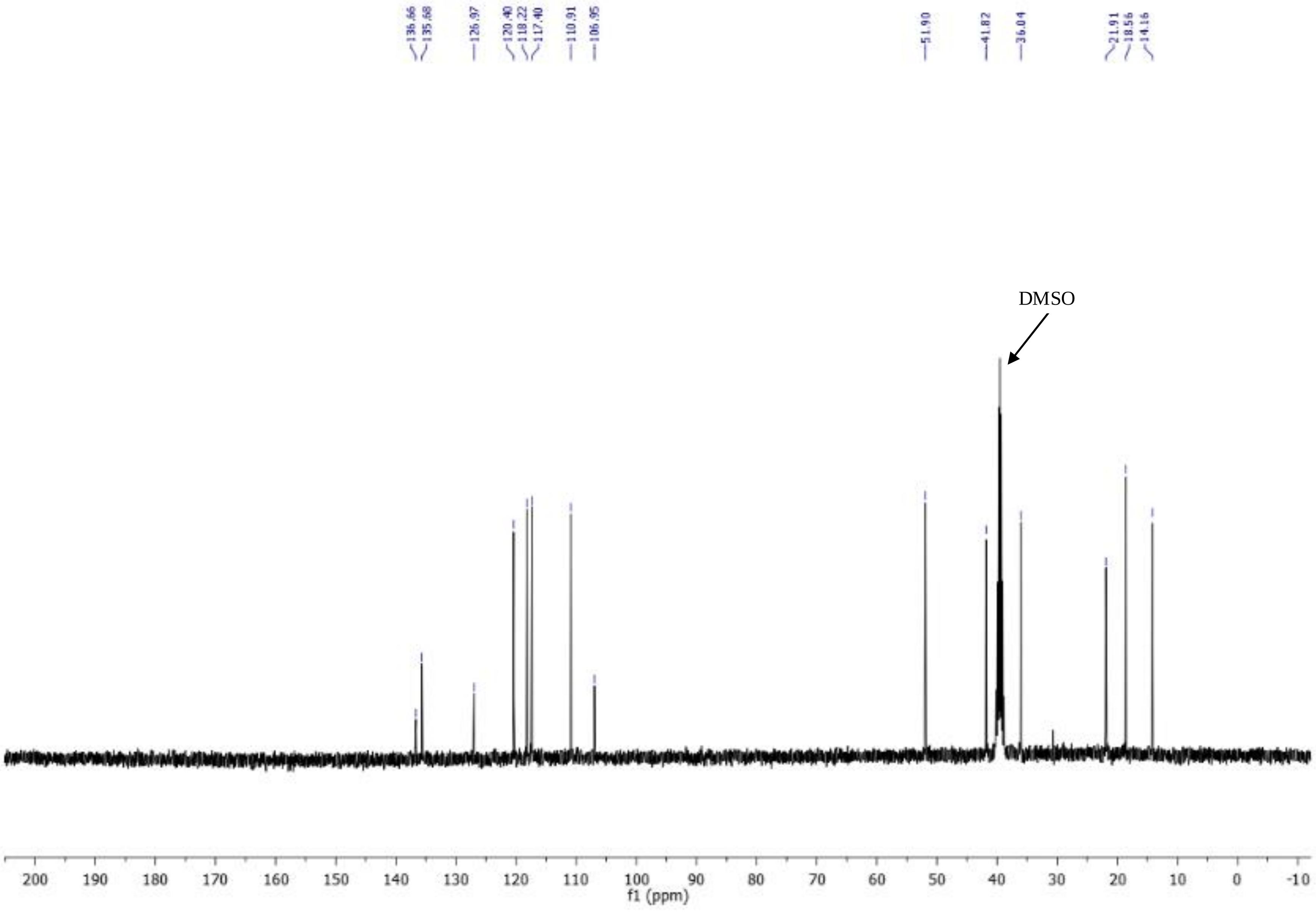

Figure S27. ^{13}C-NMR of compound **11m**

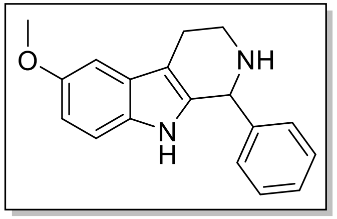

6-methoxy-1-phenyl-2,3,4,9-tetrahydro-1H-pyrido[3,4-b]indole 11n. Yield = 65% (0.65 mmol, 182 mg, yellow solid). **M.p. =** 208-212°C. **Anal. Calcd.** For $C_{18}H_{20}N_2O$ (280.37) C, 77.11; H, 7.11; N, 9.99. **Found**: C, 77.12; H, 7.13; N, 9.96.

GC-MS (EI, 70 eV) = 278 ($M^{+\cdot}$, 100), 248, 218, 201, 178, 158, 130, 102, 77.

^{1}H-NMR δ (400 MHz, $CDCl_3$, ppm) = 7.64 (s, 1H), 7.36 – 7.31 (m, 3H), 7.30 – 7.27 (m, 2H), 7.08 – 7.05 (m, 1H), 7.00 (d, *J* = 2.4 Hz, 1H), 6.79 (dd, *J* = 8.7, 2.5 Hz, 1H), 5.11 (s, 1H), 3.87 (s, 3H), 3.35 (ddd, *J* = 12.5, 5.2, 3.9 Hz, 1H), 3.16 – 3.07 (m, 1H), 2.89 (dddd, *J* = 14.2, 8.8, 5.3, 1.9 Hz, 1H), 2.82 – 2.74 (m, 1H), 2.01 (s, 1H).

^{13}C-NMR δ (101 MHz, $CDCl_3$, ppm) = 154.08, 141.88, 135.51, 131.04, 128.89, 128.59, 128.26, 127.82, 111.62, 111.56, 110.07, 100.54, 58.23, 56.05, 42.92, 22.67. Spectroscopic data are consistent with those reported in literature.[2]

FT-IR (cm^{-1}) = 3399.3, 2936.6, 2831.1, 1585.1, 1479.6, 1451.2, 1288.8, 1215.8, 1029, 728.75, 700.34.

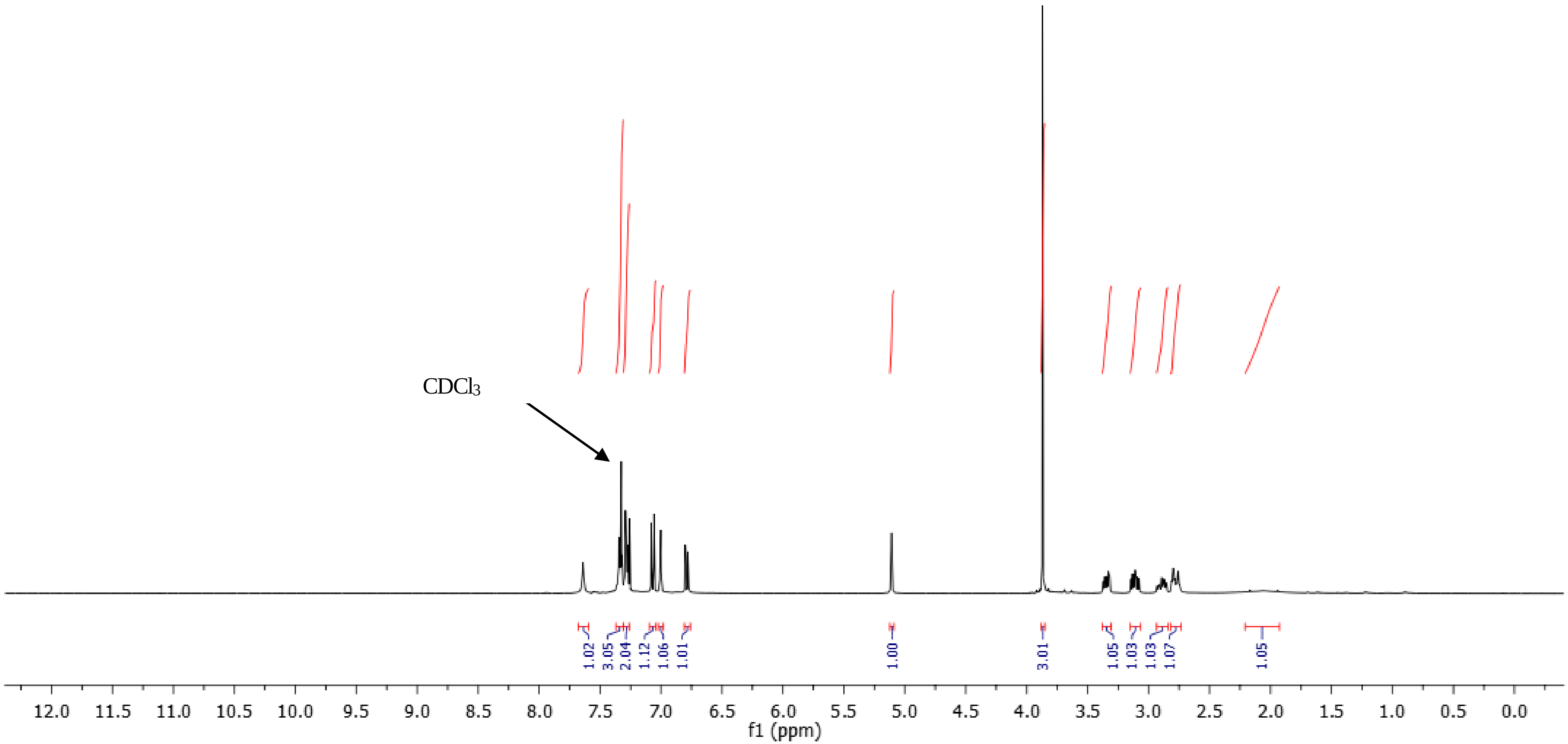

Figure S28. ^{1}H-NMR of compound **11n**

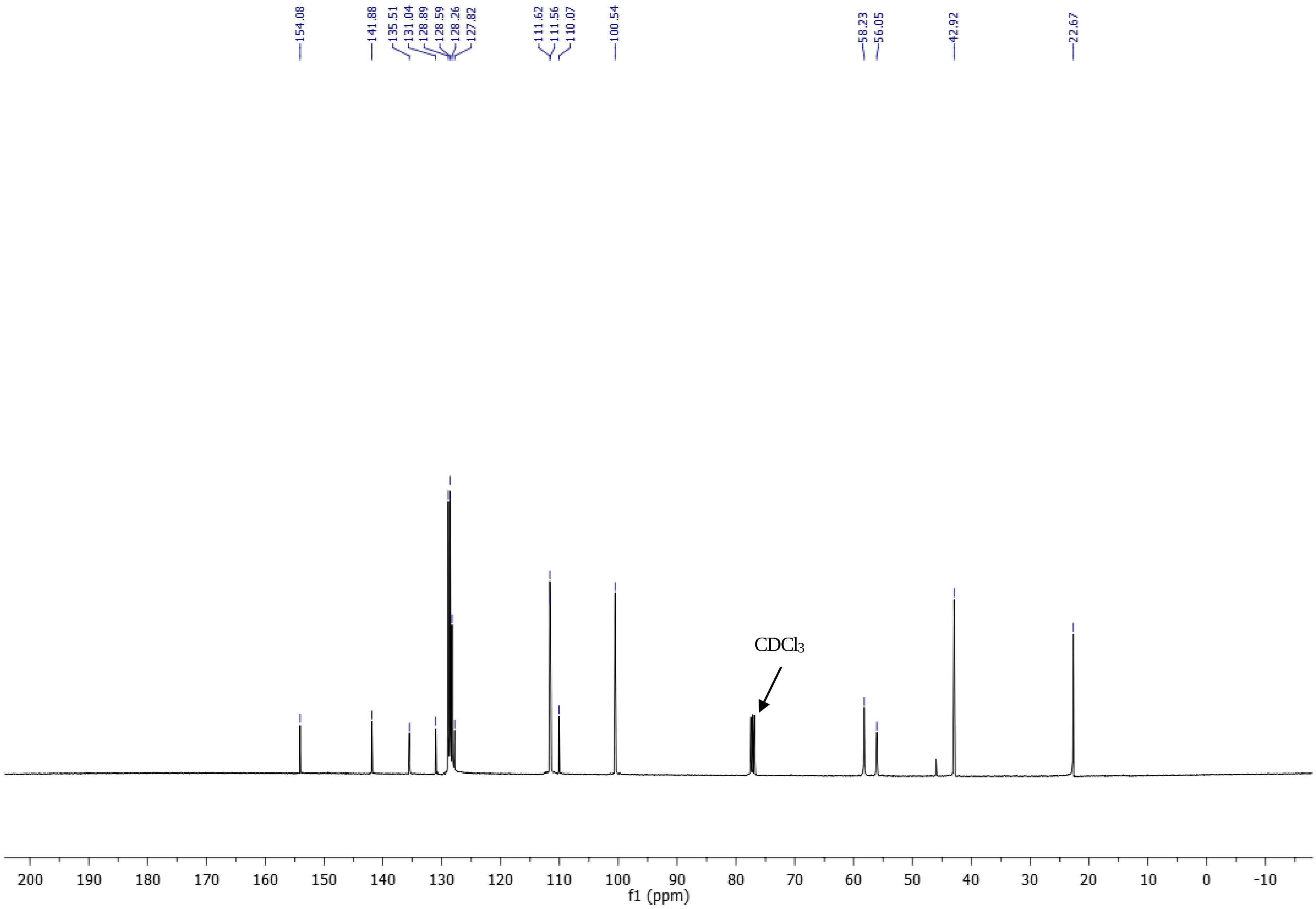

Figure S29. ^{13}C-NMR of compound **11n**

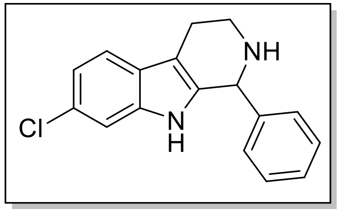

7-chloro-1-phenyl-2,3,4,9-tetrahydro-1H-pyrido[3,4-b]indole 11o. Yield = 51% (0.51 mmol, 143 mg, white solid). **M.p. =** 201-203°C. **Anal. Calcd.** For $C_{17}H_{15}ClN_2$ (282.77) C, 72.21; H, 5.35; N, 9.91. **Found**: C, 72.18; H, 5.33; N, 9.93.

GC-MS (EI, 70 eV) = 282 ($M^{+\cdot}$), 253, 217, 200, 178, 143, 108, 77, 51

^{1}H-NMR δ (400 MHz, $CDCl_3$, ppm) =7.71 (s, 1H), 7.43 (d, *J* = 8.4 Hz, 1H), 7.36 – 7.32 (m, 3H), 7.30 – 7.26 (m, 2H), 7.15 (t, *J* = 2.3 Hz, 1H), 7.09 – 7.06 (m, 1H), 5.11 (t, *J* = 1.9 Hz, 1H), 3.39 – 3.31 (m, 1H), 3.15 – 3.07 (m, 1H), 2.94 – 2.85 (m, 1H), 2.82 – 2.74 (m, 1H), 2.03 – 1.93 (s, 1H).

^{13}C-NMR δ (101 MHz, $CDCl_3$, ppm) = 141.56, 136.31, 135.31, 129.03, 128.57, 128.46, 127.53, 126.12, 120.14, 119.14, 110.95, 110.41, 58.11, 42.81, 22.50.

FT-IR (cm^{-1}) = 3147.5, 2921.2, 2844.4, 1620.2, 1454.5, 1301, 1058.6, 905.05, 800, 593.94.

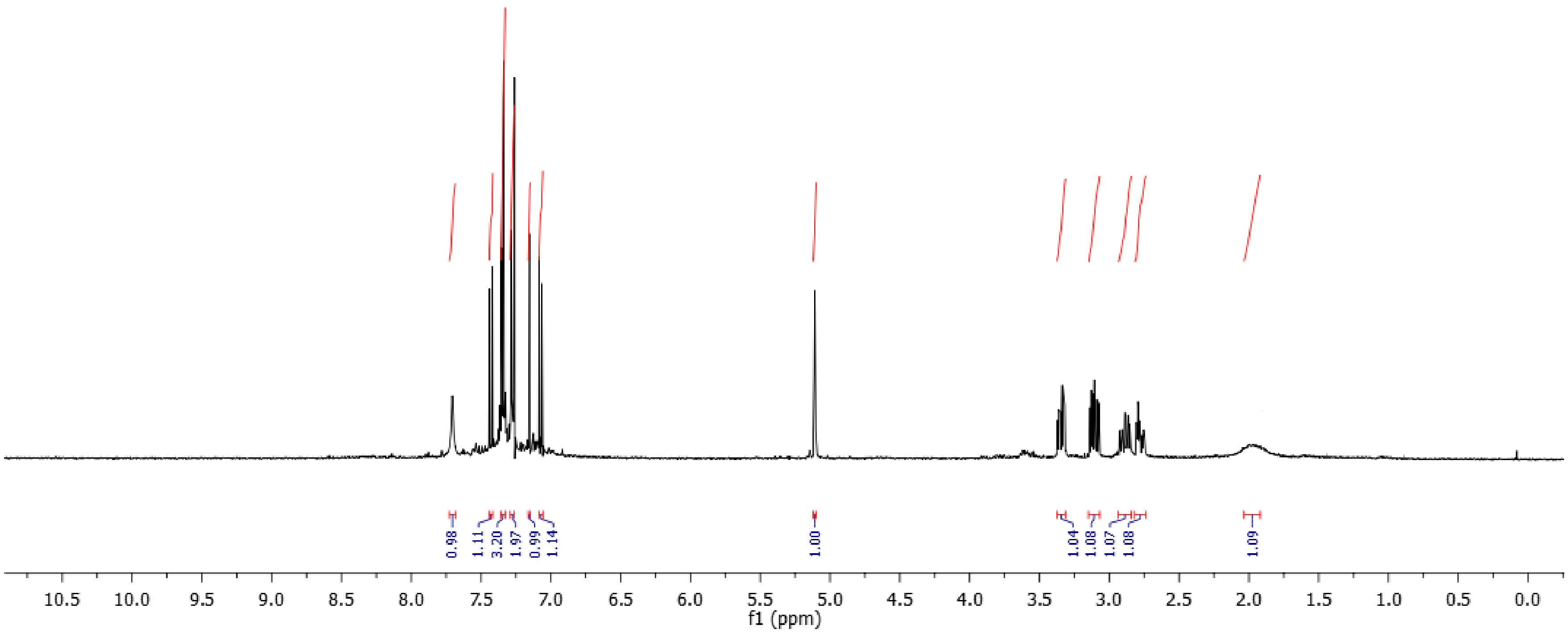

Figure S30. ^{1}H-NMR of compound **11o**

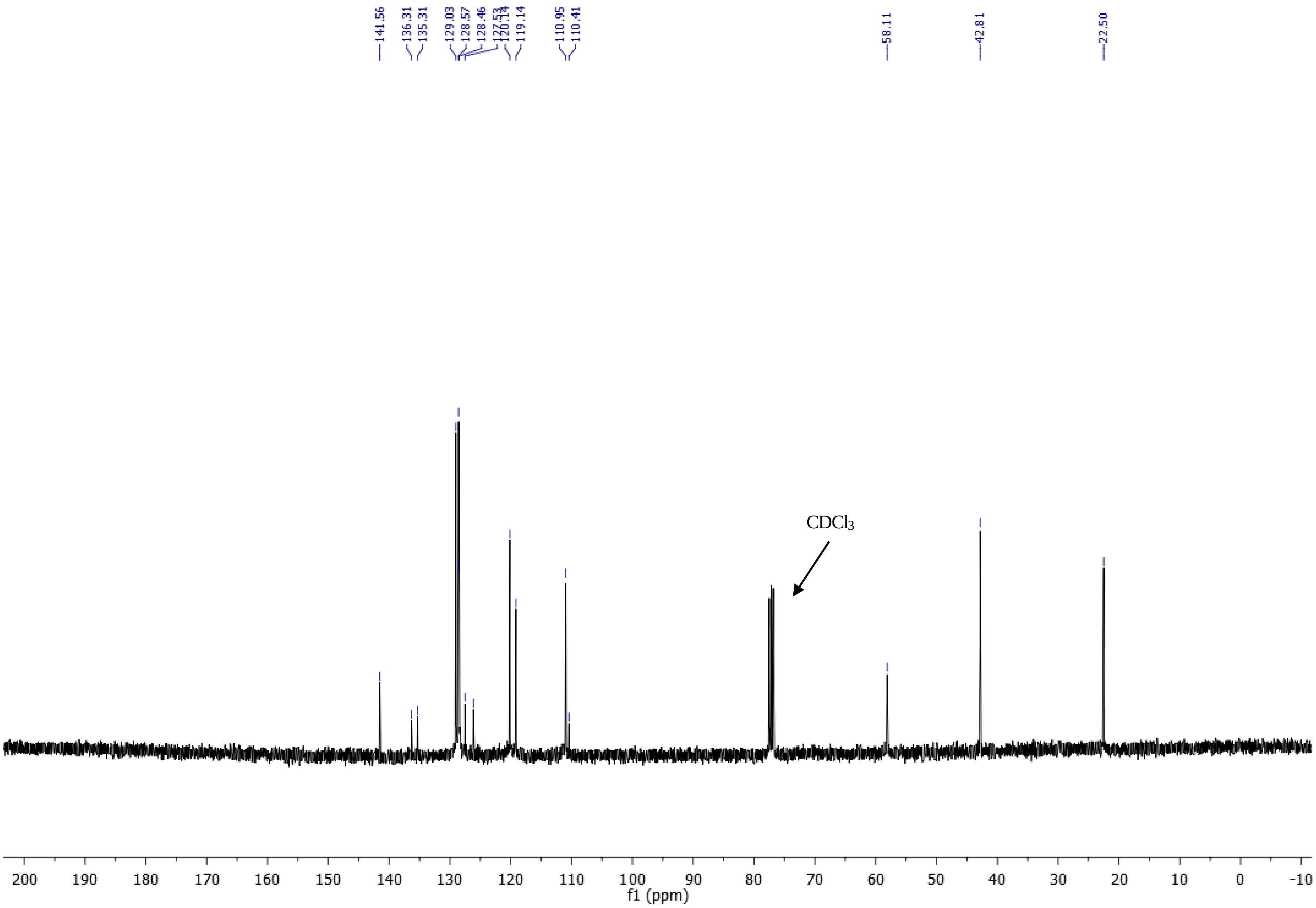

Figure S31. ^{13}C-NMR of compound **11o**

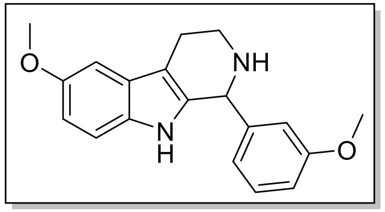

6-methoxy-1-(3-methoxyphenyl)-2,3,4,9-tetrahydro-1H-pyrido[3,4-b]indole 11p. Yield = 58% (0.58 mmol, 179 mg, light brown-yellow solid). **M.p. =** 218-220°C. **Anal. Calcd.** For $C_{19}H_{20}N_2O_2$ (308.38) C, 74.00; H, 6.54; N, 9.08. **Found**: C, 74.02; H, 6.51; N, 9.03.

GC-MS (70eV, EI) = 308 ($M^{+\cdot}$), 279, 262, 248, 221, 201, 179, 158, 130, 102, 77.

^{1}H NMR δ (400 MHz, $CDCl_3$, ppm) = 7.61 (s, 1H), 7.28 – 7.21 (m, 2H), 7.08 (d, *J* = 8.8 Hz, 1H), 6.99 (d, *J* = 2.4 Hz, 1H), 6.92 – 6.84 (m, 3H), 6.79 (m, 1H), 5.13 (s, 1H), 3.87 (s, 3H), 3.75 (s, 3H), 3.39 - 3.36 (m, 1H), 3.16 – 3.06 (m, 1H), 2.92 – 2.87 (m, 1H), 2.82 – 2.74 (m, 1H).

^{13}C NMR δ (101 MHz, $CDCl_3$, ppm) = 160.09, 154.13, 142.83, 134.87, 131.11, 129.93, 127.77, 120.91, 114.02, 111.68, 109.89, 100.58, 58.21, 56.07, 55.41, 42.86, 22.34. Spectroscopic data are consistent with those reported in literature.[6]

FT-IR (cm^{-1}) = 3398, 2837, 1603, 1859, 1491, 1485, 1450, 1261, 1041, 741.

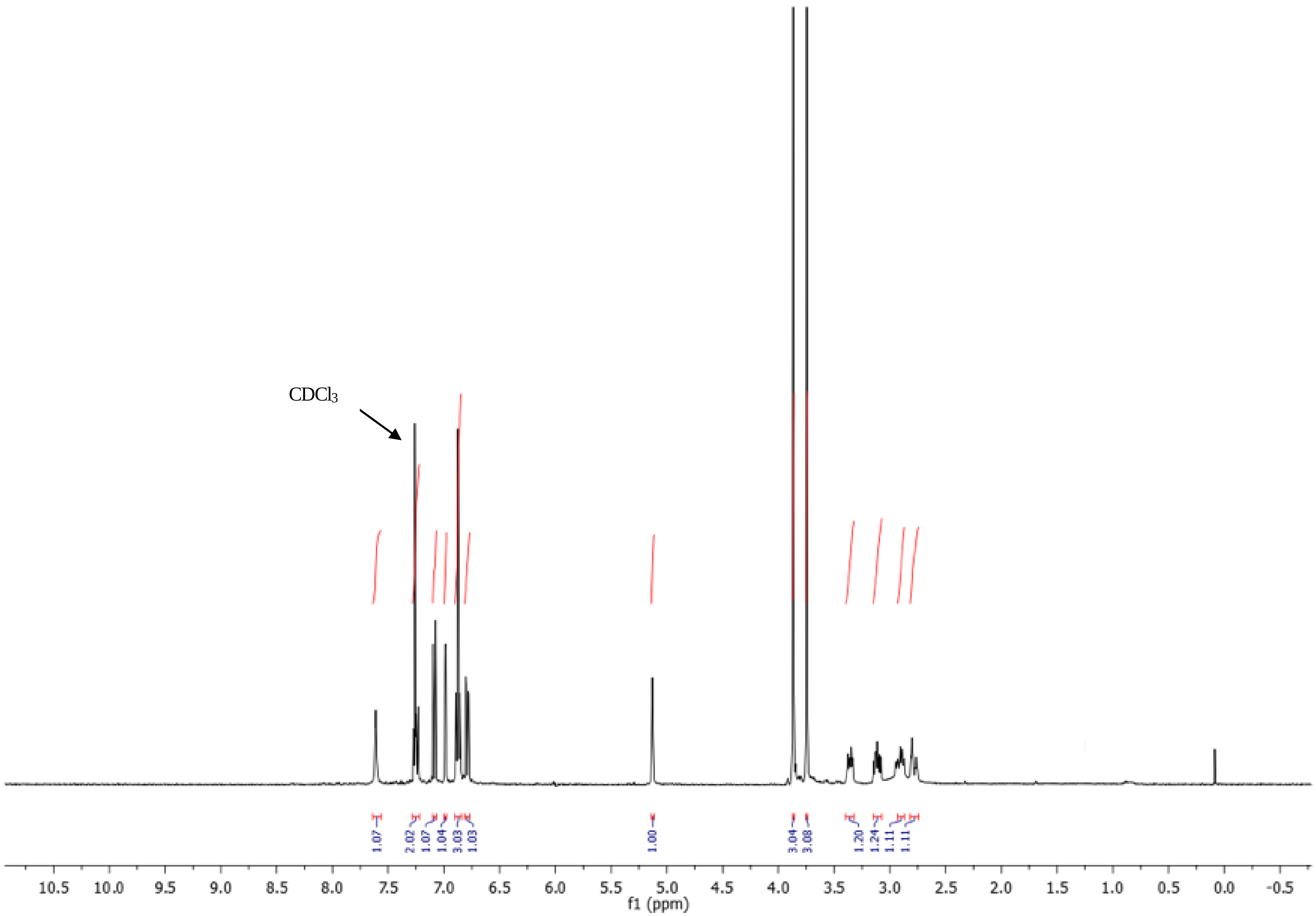

Figure S32. ^{1}H-NMR of compound **11p**

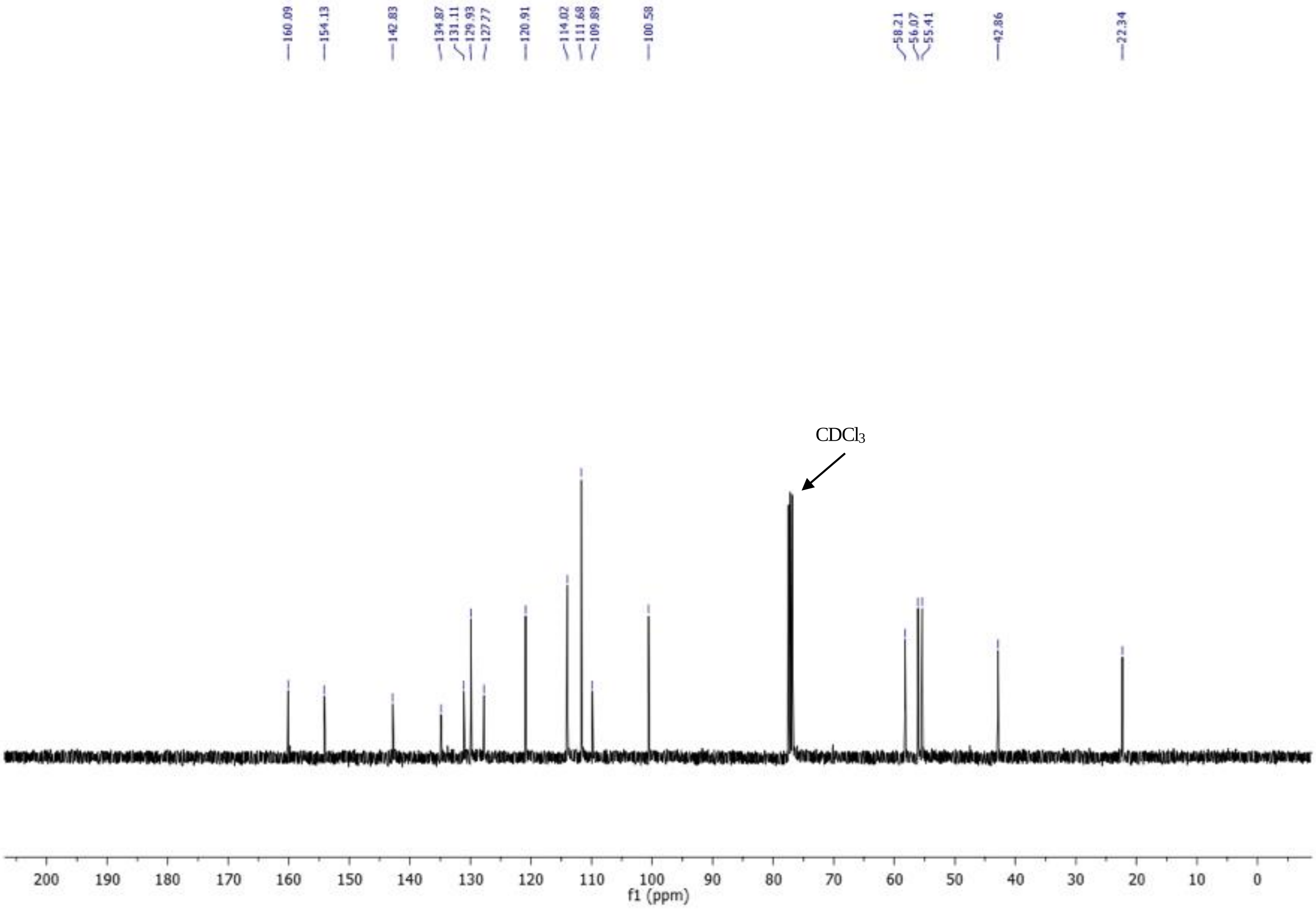

Figure S33. ^{13}C-NMR of compound **11p**

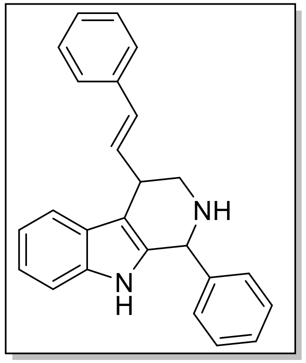

(E)-1-phenyl-4-styryl-2,3,4,4a,9,9a-hexahydro-1H-pyrido[3,4-b]indole 11q. Yield = 42% (0.42 mmol, 147 mg, yellow solid). **M.p.** = 208-211°C. **Anal. Calcd.** For $C_{25}H_{24}N_2$ (352.48) C, 85.19; H, 6.86; N, 7.95. **Found**: C, 85.21; H, 6.85; N 7.94.

GC-MS (70eV, EI) = 350 ($M^{+\cdot}$), 321 (100), 244, 217, 152, 121.

^{1}H NMR δ (400 MHz, $CDCl_3$, ppm) = 7.61 (d, *J* = 7.9 Hz, 2H), 7.36 (ddd, *J* = 20.6, 15.9, 7.3 Hz, 10H), 7.24 (d, *J* = 7.9 Hz, 2H), 7.13 (t, *J* = 7.6 Hz, 1H), 7.02 (d, *J* = 7.1 Hz, 1H), 6.70 (d, *J* = 15.8 Hz, 1H), 6.36 (dd, *J* = 15.7, 8.3 Hz, 1H), 5.21 (s, 1H), 3.99 (d, J = 6.1 Hz, 1H), 3.42 (dd, *J* = 12.5, 5.6 Hz, 1H), 3.01 (dd, *J* = 12.8, 8.3 Hz, 1H).

^{13}C NMR δ (101 MHz, $CDCl_3$, ppm) = 141.56, 137.55, 136.01, 134.93, 132.05, 131.52, 129.04, 128.69, 128.67, 128.45, 127.35, 126.40, 121.84, 119.79, 119.69, 112.13, 110.97, 58.13, 49.56, 39.22.

FT-IR (cm^{-1}) = 3410, 3057, 3026, 2924, 2853, 1494, 1451, 966, 909, 741, 698.

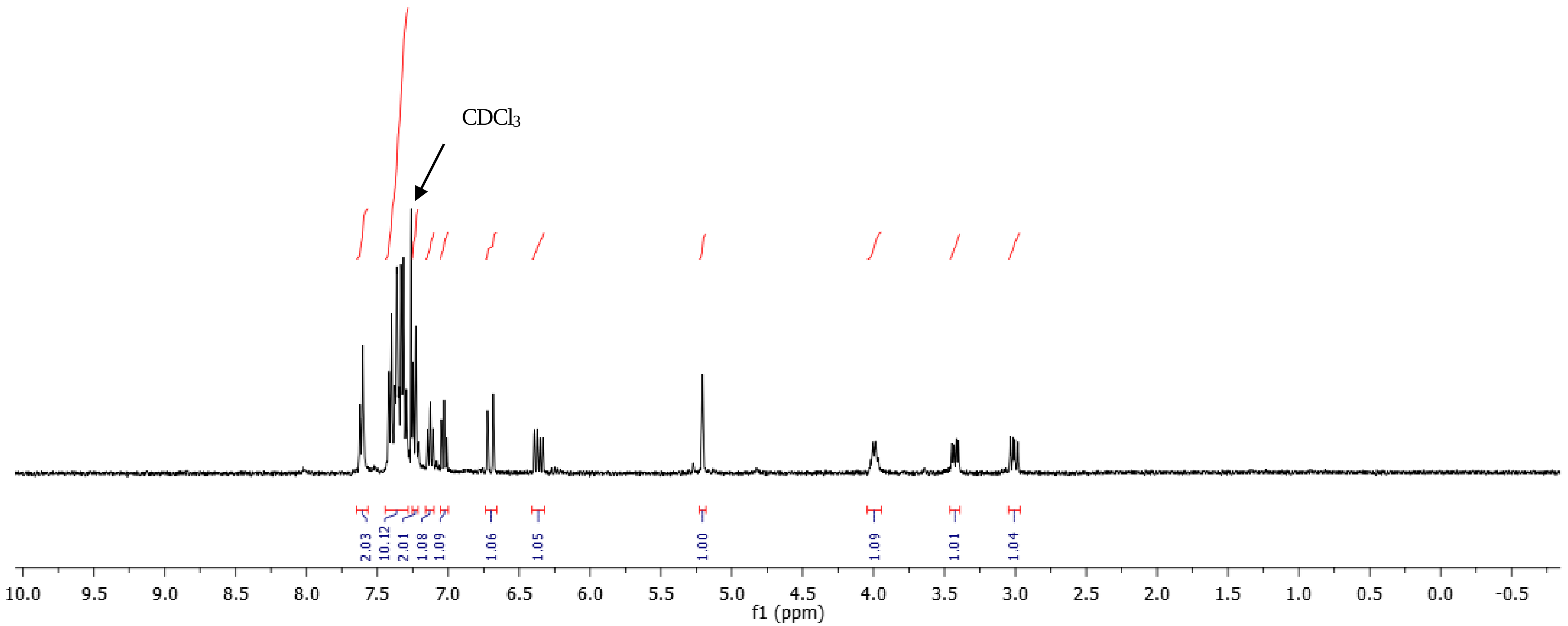

Figure S34. ¹H-NMR of compound **11q**

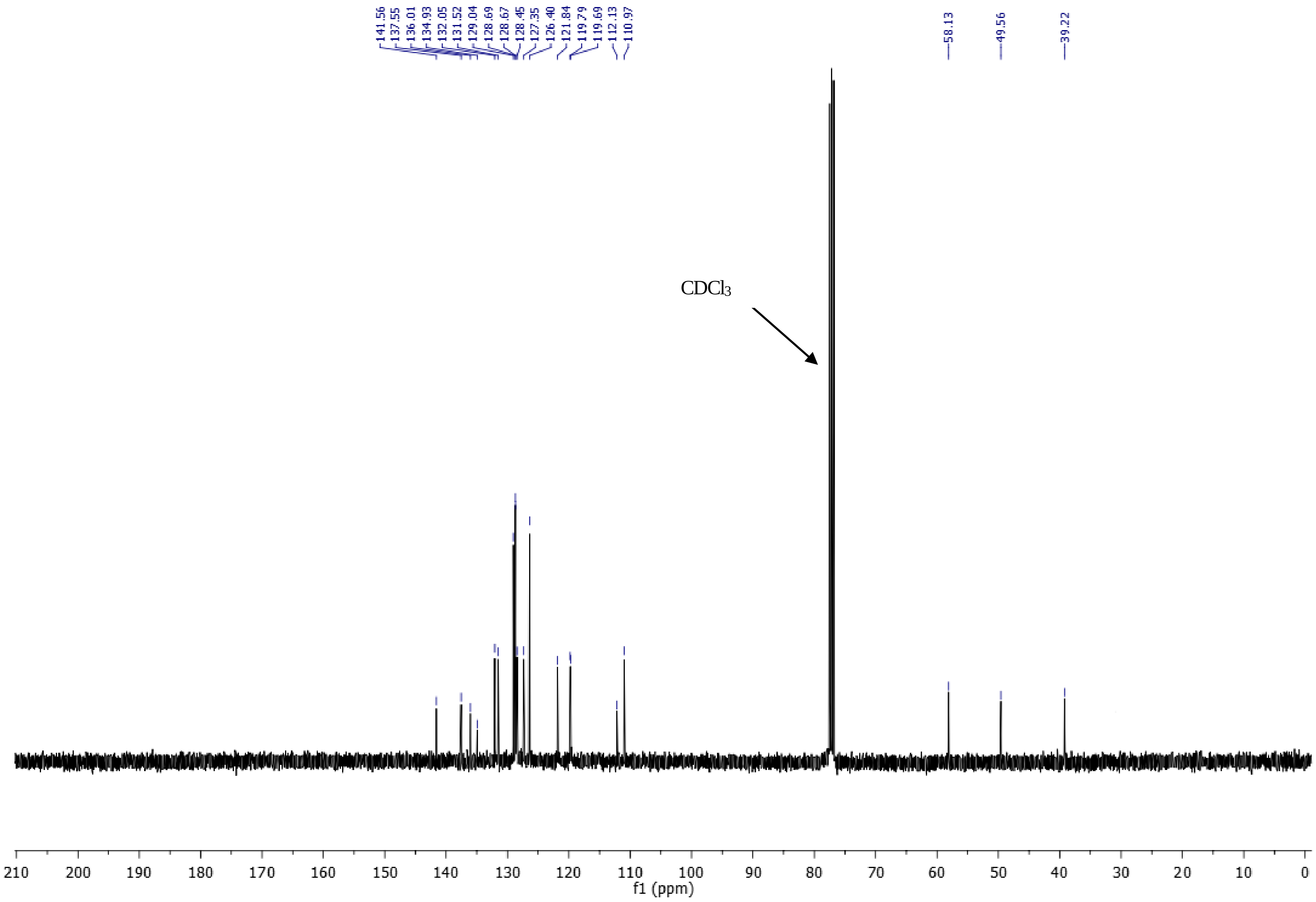

Figure S35. ^{13}C-NMR of compound **11q**

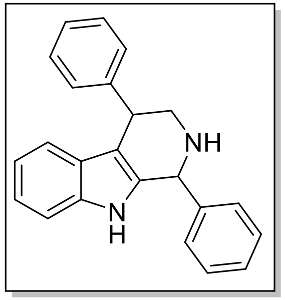

1,4-diphenyl-2,3,4,9-tetrahydro-1H-pyrido[3,4-b]indole 11r. Yield = 48% (0.48 mmol, 155 mg, yellow solid). **M.p. =** 148-150°C. **Anal. Calcd.** For $C_{23}H_{20}N_2$ (324.43) C, 85.15; H, 6.21; N, 8.63. **Found**: C, 85.12; H, 6.19; N, 8.61.

GC-MS (70eV, EI) = 324($M^{+\cdot}$), 295 (100), 218, 189, 146, 108, 77.

^{1}H NMR δ (400 MHz, $CDCl_3$, ppm) = 7.63 (s, 1H), 7.44 – 7.26 (m, 10H), 7.21 (d, *J* = 8.1 Hz, 1H), 7.10 – 7.05 (m, 1H), 6.92 – 6.86 (m, 1H), 6.83 (d, *J* = 8.0 Hz, 1H), 5.29 (d, *J* = 2.0 Hz, 1H), 4.42 – 4.37 (m, 1H), 3.56 (dd, *J* = 12.7, 5.3 Hz, 1H), 3.05 (dd, *J* = 12.7, 8.8 Hz, 1H), 1.93 (s, 1H).

^{13}C NMR δ (101 MHz, $CDCl_3$, ppm) = 143.13, 141.59, 136.10, 136.00, 129.08, 128.69, 128.54, 128.53, 128.49, 126.85, 126.67, 121.71, 120.00, 119.43, 112.68, 110.90, 58.39, 53.09, 41.86.

FT-IR (cm^{-1}) = 3394, 3062, 3045, 2920, 2853, 1454, 1089, 1014, 799, 737, 700.

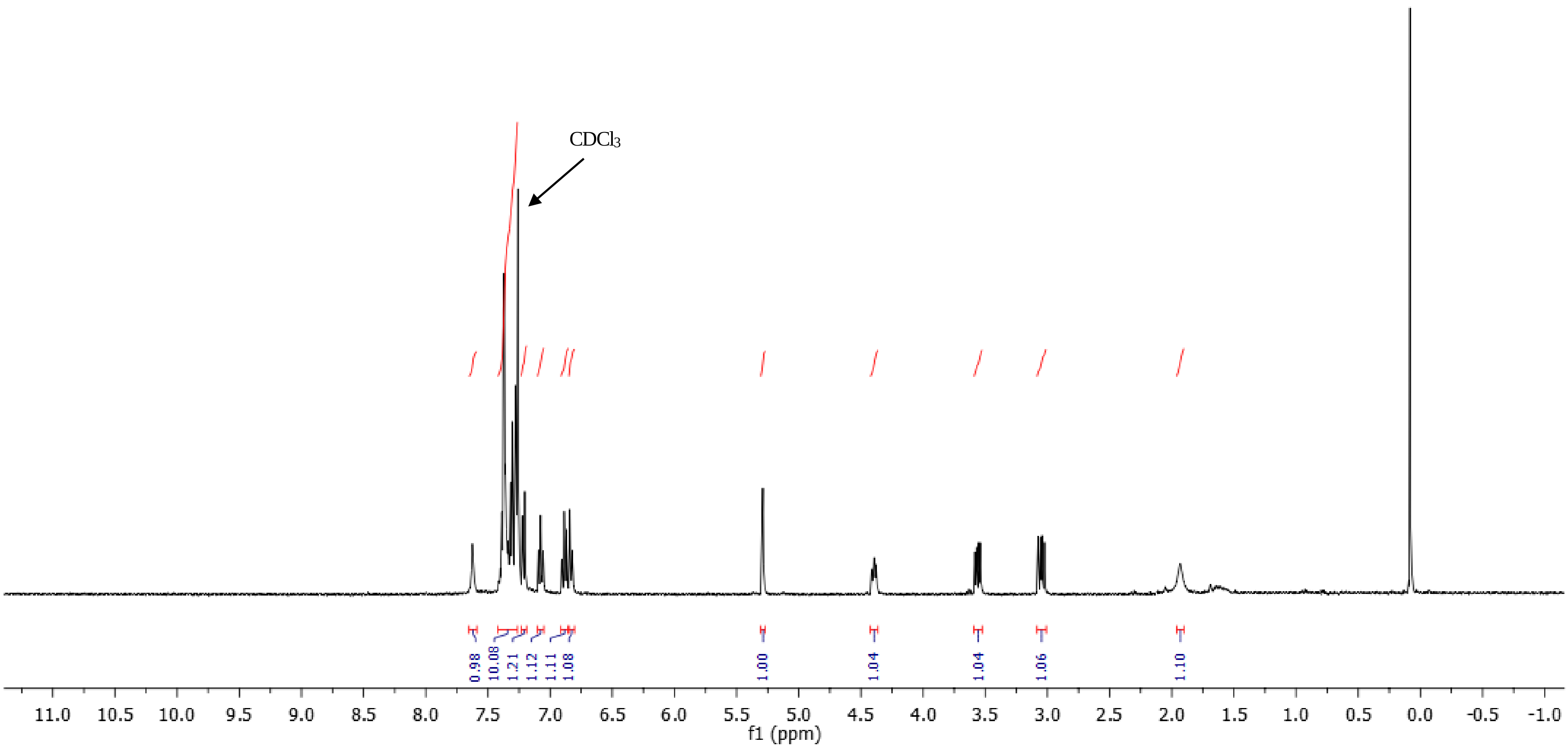

Figure S36. ^{1}H-NMR of compound **11r**

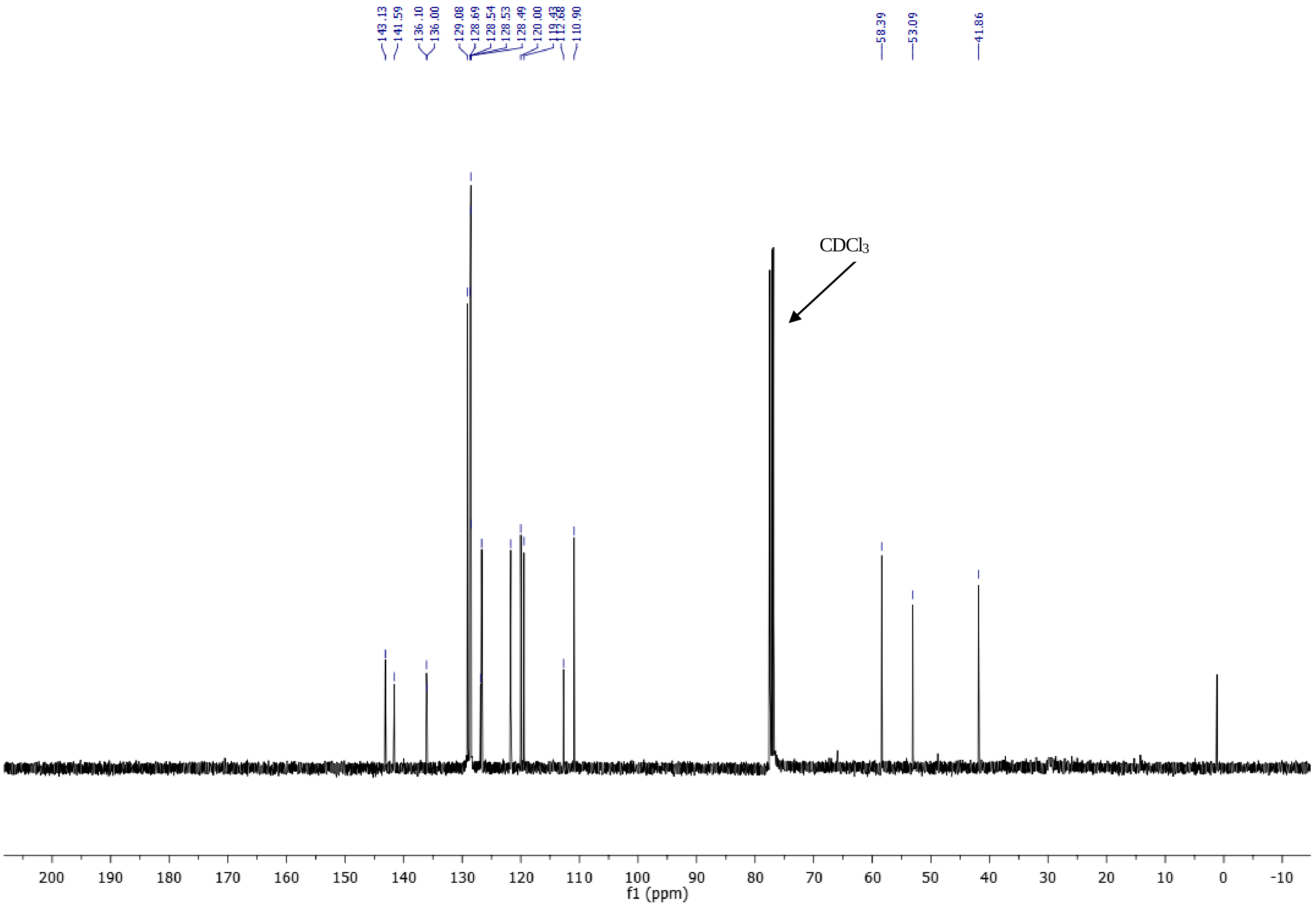

Figure S37. ^{13}C-NMR of compound **11r**

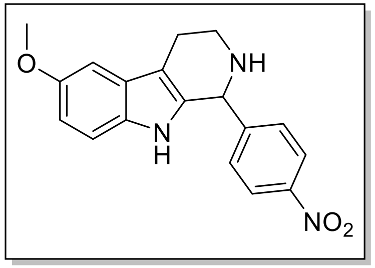

6-methoxy-1-(4-nitrophenyl)-2,3,4,9-tetrahydro-1H-pyrido[3,4-b]indole 11s. Yield = 48% (0.48 mmol, 150 mg, orange-yellow solid). **M.p. =** 190-192°C. **Anal. Calcd.** For $C_{18}H_{17}N_3O_2$, (312.80) C, 66.86; H, 5.30; N, 13.00. **Found**: C, 66.83; H, 5.31; N 12.98.

ESI-MS (+) = 324 $[M+H]^+$, 346 $[M+Na]^+$

^{1}H NMR δ (400 MHz, CD_3OD, ppm) = 8.27 (d, *J* = 8.8 Hz, 2H), 7.57 (d, *J* = 8.8 Hz, 2H), 7.13 (d, *J* = 8.3 Hz, 1H), 7.00 (d, *J* = 2.3 Hz, 1H), 6.76 (m, 1H), 5.56 (s, 1H), 3.82 (s, 3H), 3.41 - 3.37 (m, 1H), 3.28 – 3.22 (m, 1H), 3.03 - 2.91 (m, 2H).

^{13}C NMR δ (101 MHz, CD_3OD, ppm) = 155.43, 149.68, 133.42, 131.59, 130.48, 130.37, 128.12, 124.88, 113.23, 112.91, 109.91, 101.18, 57.64, 56.25, 42.46, 30.74.

FT-IR (cm^{-1}) = 3409, 3230, 2847, 1597, 1519, 1485, 1450, 1347, 1298, 1091, 1010, 855, 738.

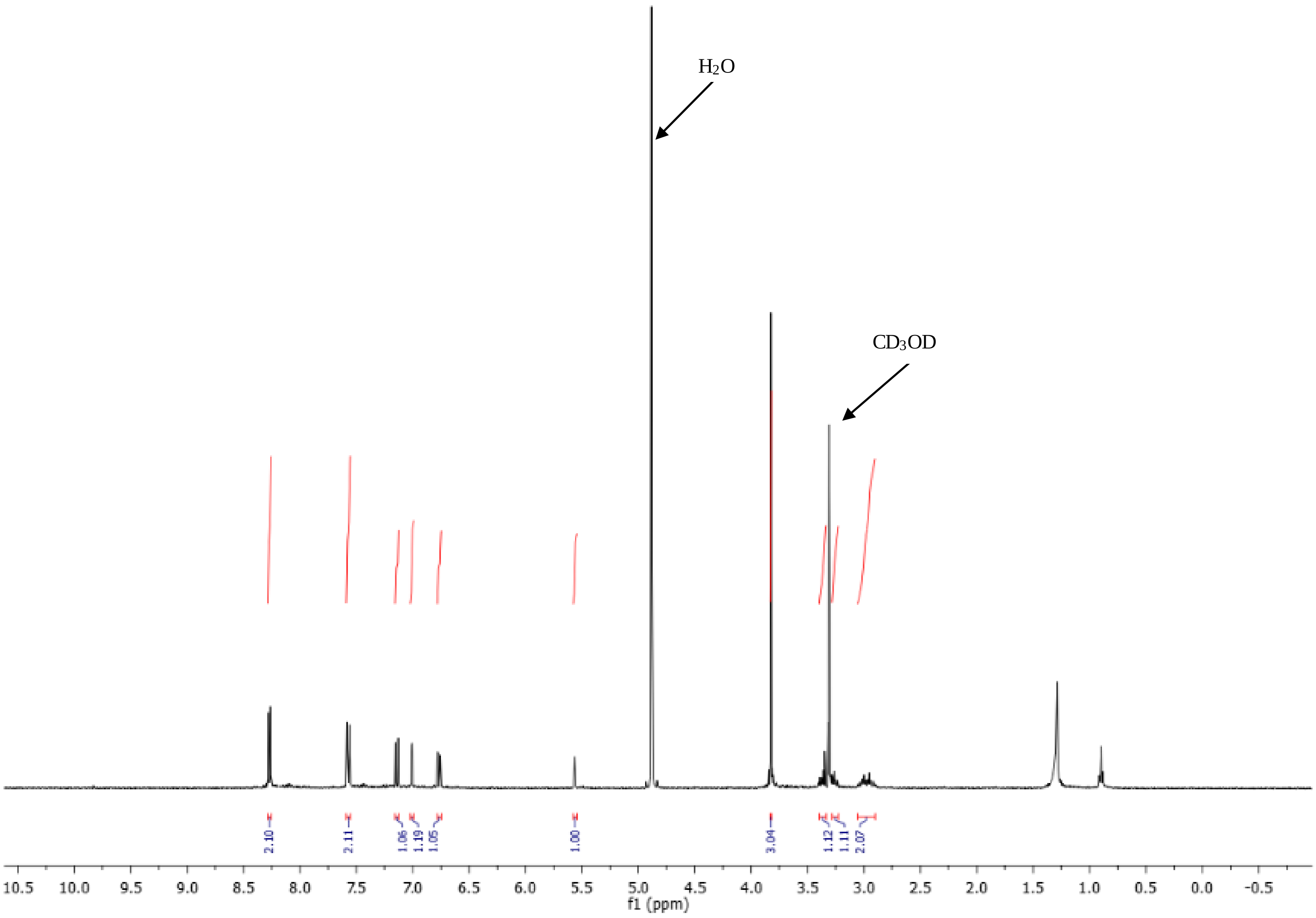

Figure S38. ^{1}H-NMR of compound **11s**

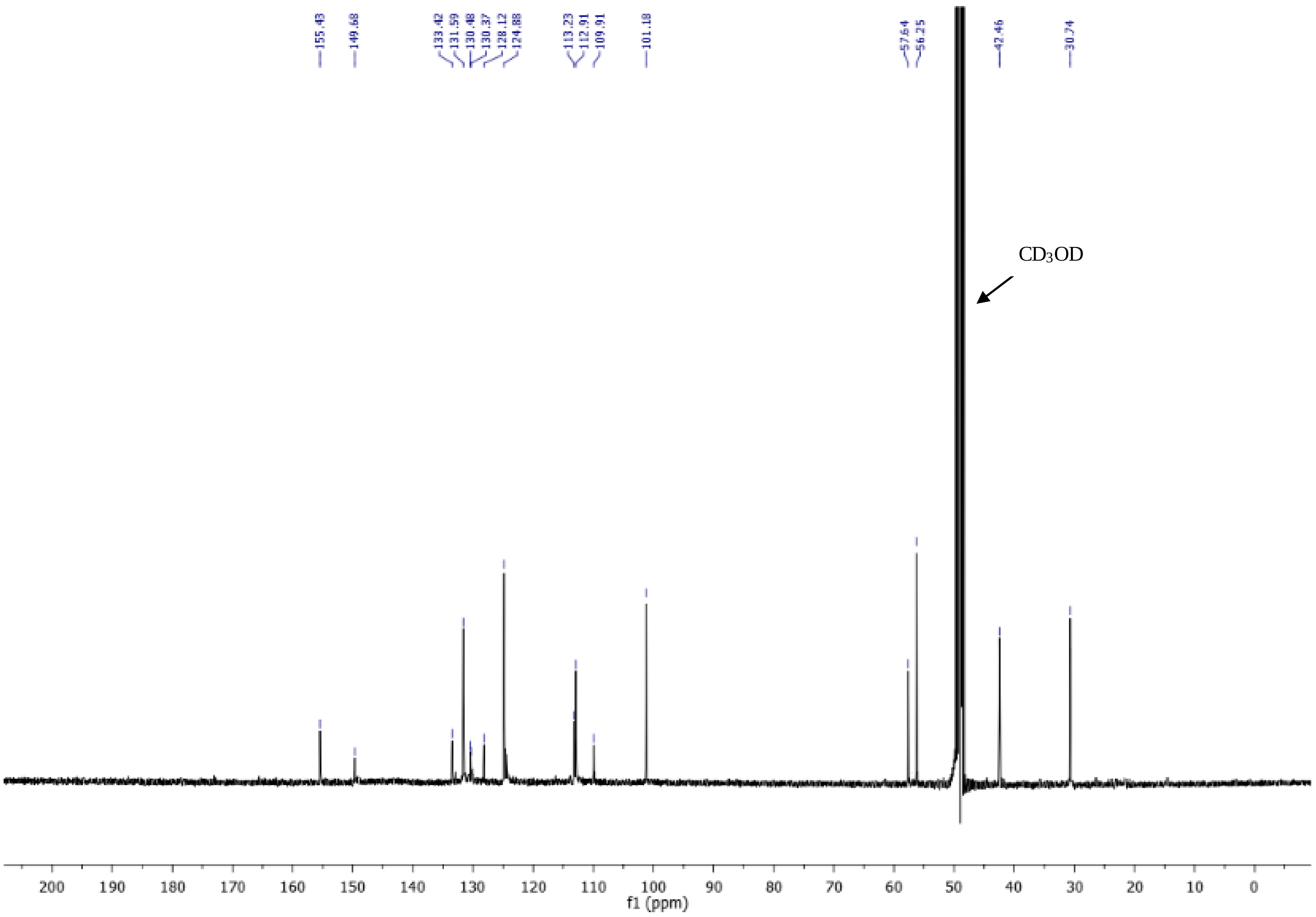

Figure S39. ^{13}C-NMR of compound **11s**

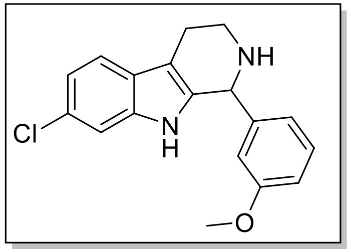

7-chloro-1-(3-methoxyphenyl)-2,3,4,9-tetrahydro-1H-pyrido[3,4-b]indole 11t. Yield = 42% (0.42 mmol, 136 mg, bright orange solid). **M.p. =** 175-178°C. **Anal. Calcd.** For $C_{18}H_{17}ClN_2O_2$ (323.35) C, 69.12; H, 5.48; N, 8.96. **Found**: C, 69.10; H, 5.45; N, 8.95.

GC-MS (70eV, EI) = 312 ($M^{+\cdot}$, 100), 283, 252, 233, 218, 205, 178, 143, 124.

^{1}H NMR δ (400 MHz, $CDCl_3$, ppm) = 7.79 (s, 1H, -NH indole), 7.41 (m, 1H), 7.27 – 7.22 (m, 1H), 7.15 (m, 1H), 7.07 (m, 1H), 6.86 (m, 3H), 5.16 (s, 1H), 3.75 – 3.70 (s, 3H), 3.41 (s, 1H, -NH pyrido ring), 3.38 – 3.32 (m ,1H), 3.13 – 3.05 (m, 1H), 2.92 – 2.86 (m, 1H), 2.79 (d, *J* = 15.3 Hz, 1H).

^{13}C NMR δ (101 MHz, $CDCl_3$, ppm) = 160.17, 141.77, 136.42, 134.04, 130.10, 127.78, 125.91, 120.98, 120.28, 119.22, 114.25, 111.06, 110.12, 57.78, 55.45, 42.32, 21.80.

FT-IR (cm^{-1}) = 3145, 2918, 2848, 1624, 1455, 1302, 1054, 905, 800, 592.

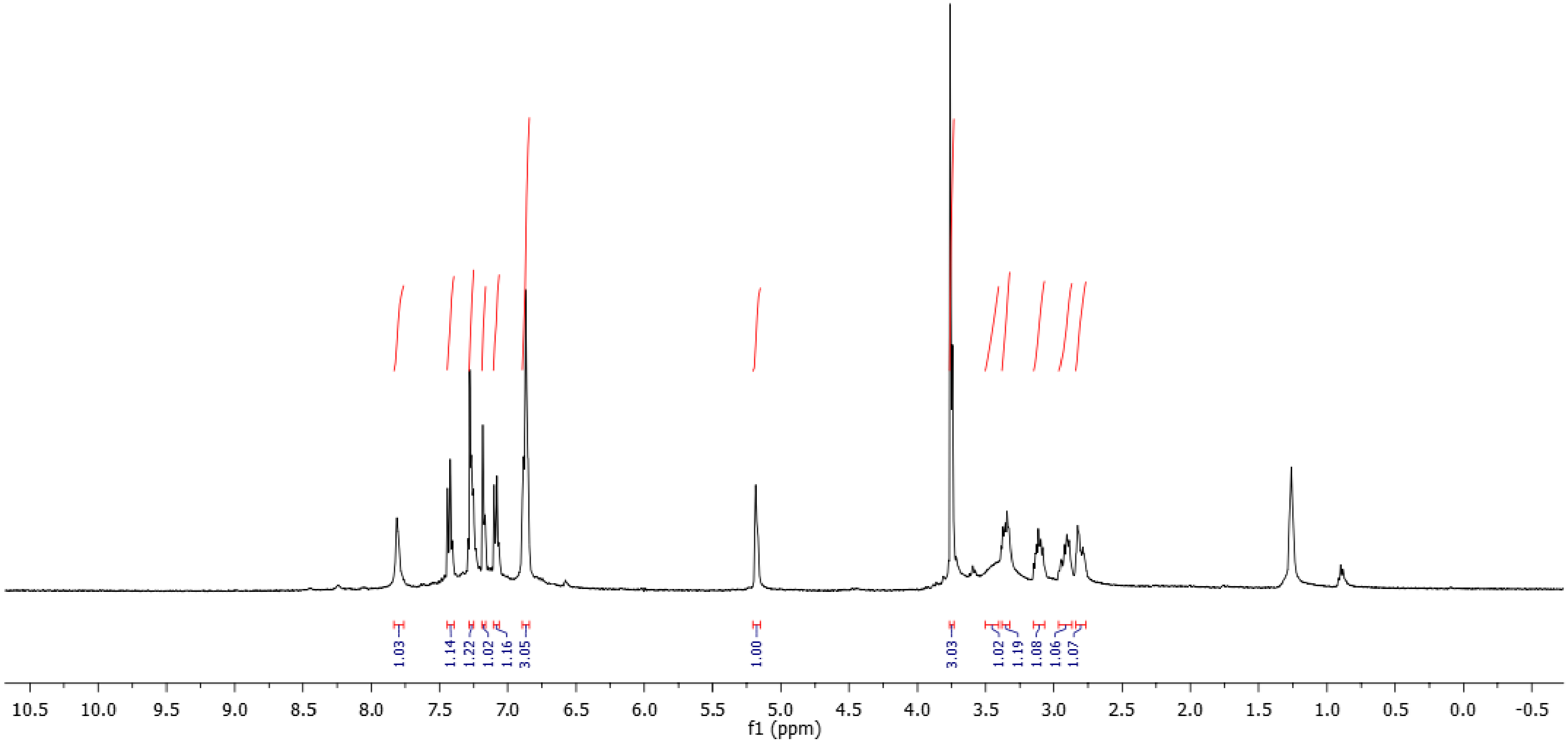

Figure S40. ^{1}H-NMR of compound **11t**

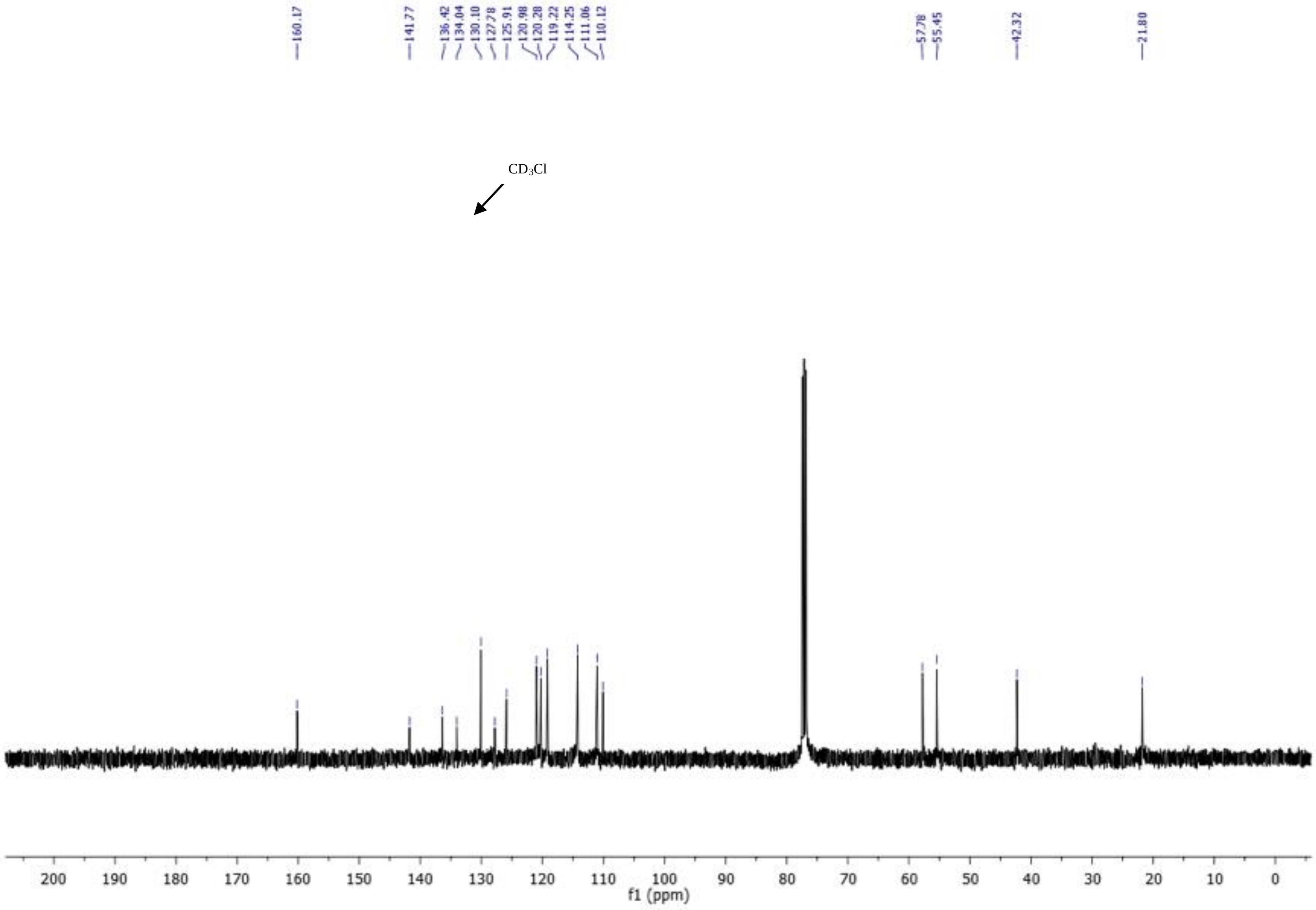

Figure S41. ^{13}C-NMR of compound **11t**

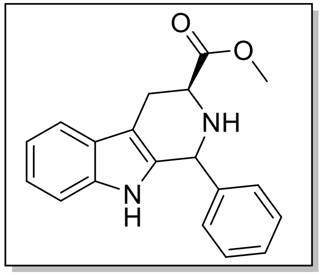

55 syn : 45 trans

methyl (*3S*)-1-phenyl-2,3,4,9-tetrahydro-1H-pyrido[3,4-b]indole-3-carboxylate 11ua/11ub. Yield = 55% (0.55 mmol, 169 mg, yellow solid). **M.p. =** 190-192°C. **Anal. Calcd.** For $C_{19}H_{20}N_2O_2$ (308.38) C, 74.00; H, 6.54; N, 9.08. **Found**: C, 74.02; H, 6.55; N, 9.05.

GC-MS (EI, 70 eV) = 306 ($M^{+\cdot}$), 275, 247, 218 (100), 189, 169, 144, 115, 77.

^{1}H-NMR δ (400 MHz, $CDCl_3$ as mixture of diastereoisomers, ppm) = 7.63 (s, 1H), 7.58 – 7.53 (m, 2H), 7.47 (s, 1H), 7.40 – 7.27 (m, 11H), 7.24 – 7.10 (m, 7H), 5.44 (s, 1H), 5.25 (s, 1H), 3.99 (dd, *J* = 11.0, 4.1 Hz, 2H), 3.81 (s, 3H), 3.72 (s, 2H), 3.34 – 3.11 (m, 3H), 3.08 – 2.98 (m, 1H), 2.39 (s, 2H).

^{13}C-NMR δ (101 MHz, $CDCl_3$ as mixture of diastereoisomer, ppm) = 174.01, 173.29, 143.59, 141.66, 140.73, 136.31, 136.26, 134.70, 133.02, 129.86, 129.10, 128.90, 128.77, 128.61, 128.35, 127.20, 127.03, 122.13, 122.09, 119.76, 119.66, 118.38, 118.33, 111.07, 109.01, 108.50, 58.80, 57.00, 55.08, 52.62, 52.42, 52.33, 25.80, 24.64. Spectroscopic data are consistent with those reported in literature.[7]

FT-IR (cm^{-1}) = 3393, 2925, 2853, 1733, 1457, 1270, 1216, 1176, 734, 703**.**

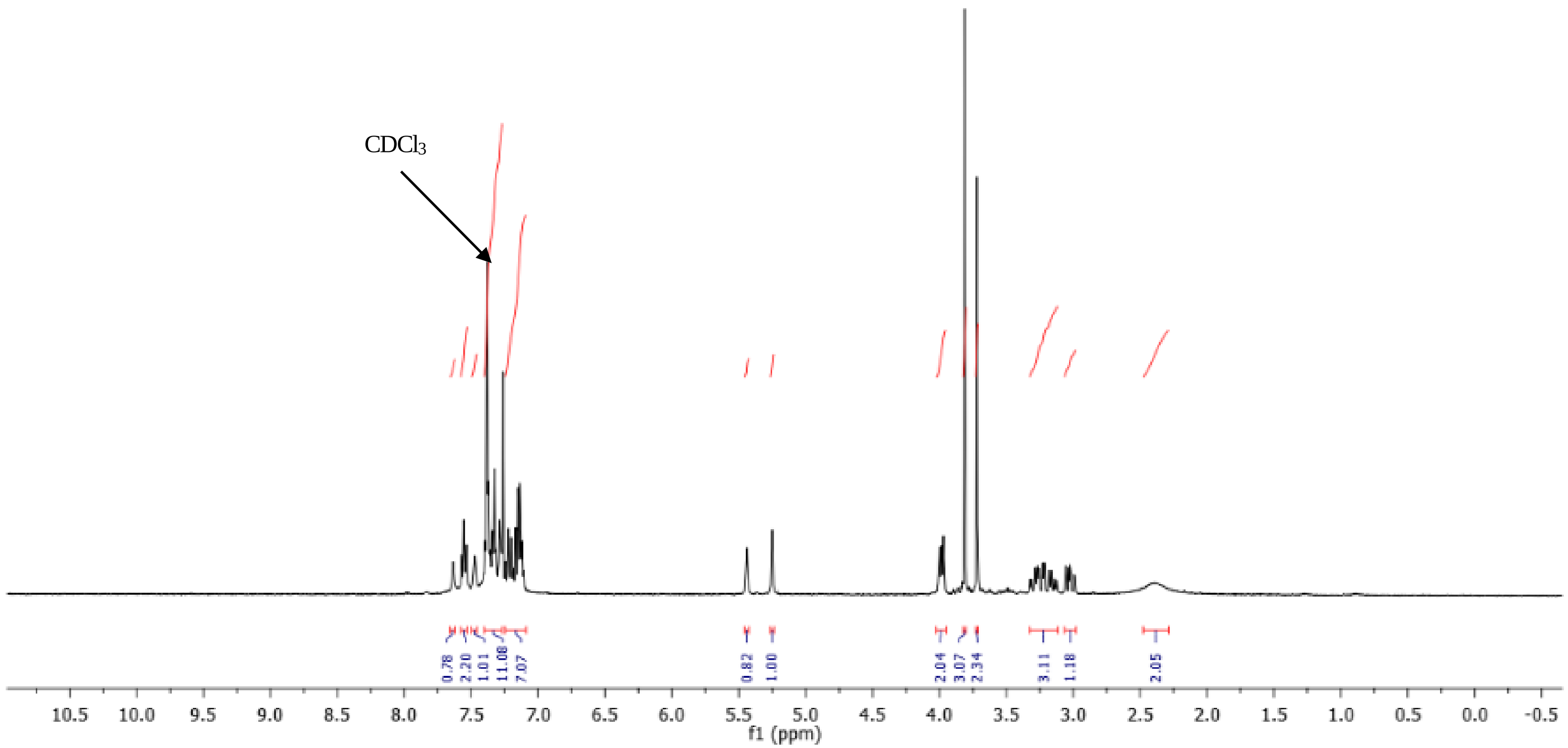

Figure S42. ^{1}H-NMR of diastereoisomer of compounds **11ua/11ub**

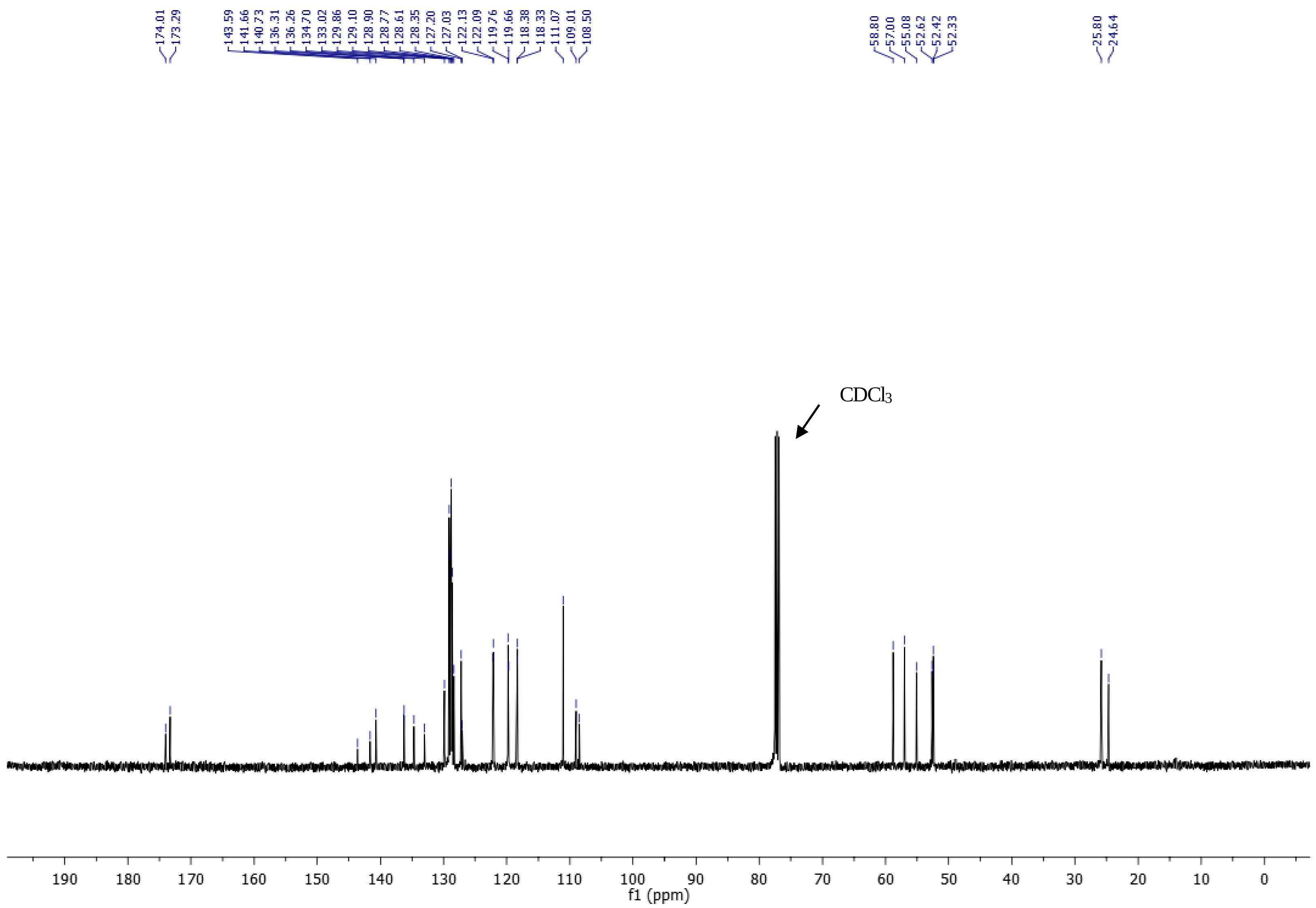

Figure S43. ^{13}C-NMR of mixture/diastereoisomer of compounds **11ua/11ub**

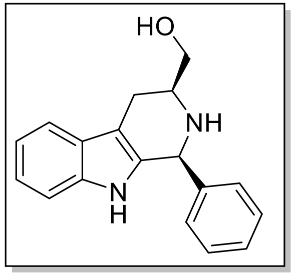

((*1S,3S*)-1-phenyl-2,3,4,9-tetrahydro-1H-pyrido[3,4-b]indol-3-yl)methanol 11wa. Yield = 31% (0.31 mmol, 86 mg, bright yellow solid). **M.p. =** 207-212°C. **Anal. Calcd.** For $C_{18}H_{18}N_2O$ (278.36) C, 77.67; H, 6.52; N, 10.06. **Found**: C, 77.69; H, 6.55; N, 10.08.

GC-MS (EI, 70 eV) = 278.0 ($M^{+\cdot}$), 245 (100), 218, 201, 169, 144, 115, 77.

^{1}H-NMR δ (400 MHz, $CDCl_3$, major diastereoisomer, ppm) = 7.58 (s, 1H, -NH indole), 7.52 (d, *J* = 6.8 Hz, 1H), 7.39 – 7.29 (m, 5H), 7.20 (d, *J* = 6.9 Hz, 1H), 7.16 – 7.10 (m, 2H), 5.15 (s, 1H), 3.83 (dd, *J* = 10.9, 3.5 Hz, 1H), 3.58 (dd, *J* = 10.7, 8.3 Hz, 1H), 3.33 – 3.24 (m, 1H), 2.79 (d, *J* = 13.0 Hz, 1H), 2.61 (dd, *J* = 18.6, 7.4 Hz, 1H), 2.40 (s, 2H, -OH and -NH).

^{13}C-NMR δ (101 MHz, $CDCl_3$, major diastereoisomer, ppm) = 141.15, 136.18, 135.06, 129.09, 128.68, 128.61, 127.32, 121.93, 119.59, 118.28, 111.02, 109.74, 66.15, 58.84, 56.52, 24.69. Spectroscopic data are consistent with those reported in literature.[8]

FT-IR (cm-1) = 3566, 3403, 3282, 3059, 2917, 2841, 1458, 1302, 1264, 1015, 911, 733, 698.

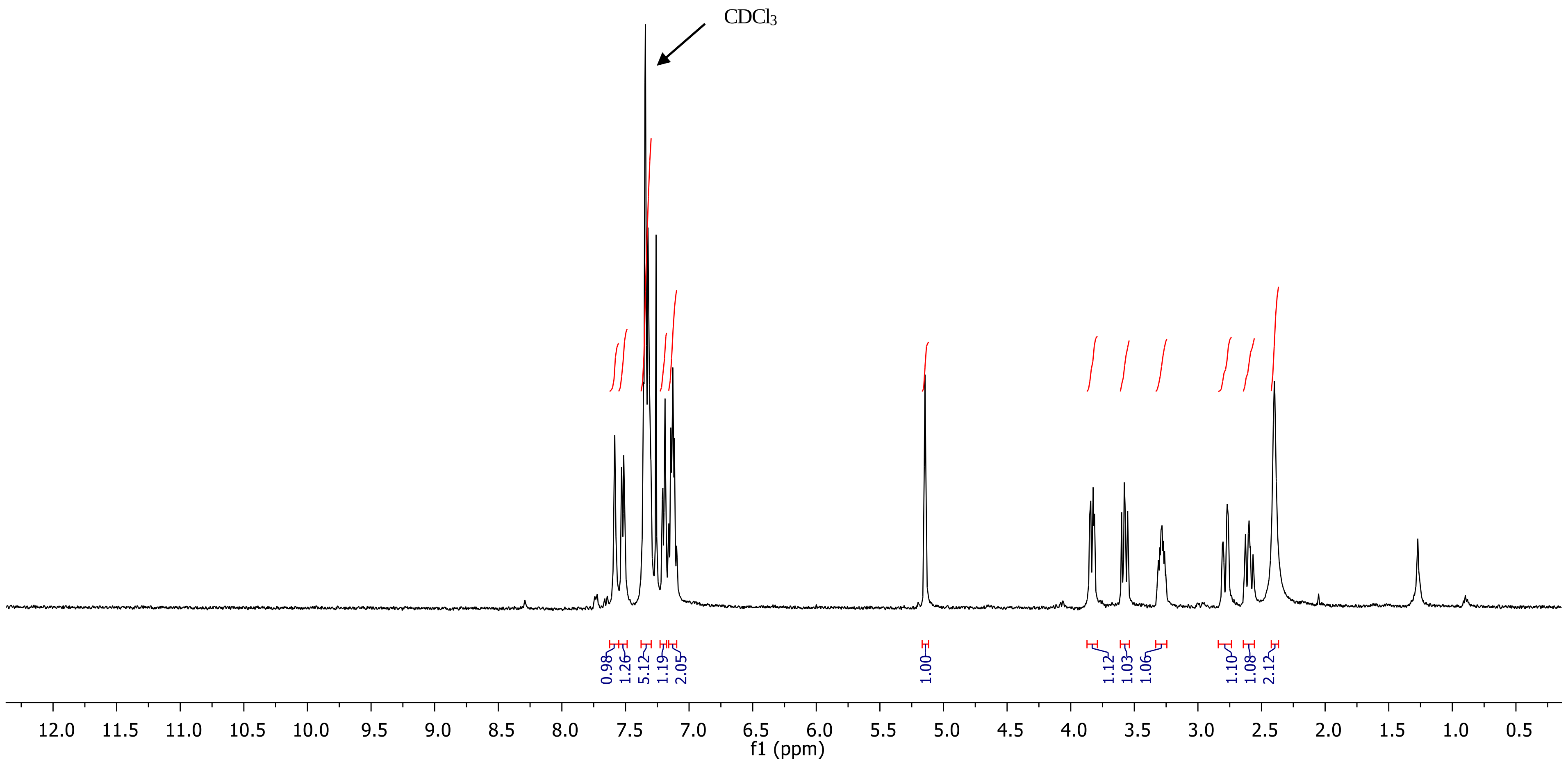

Figure S44. ^{1}H-NMR of compounds **11wa**

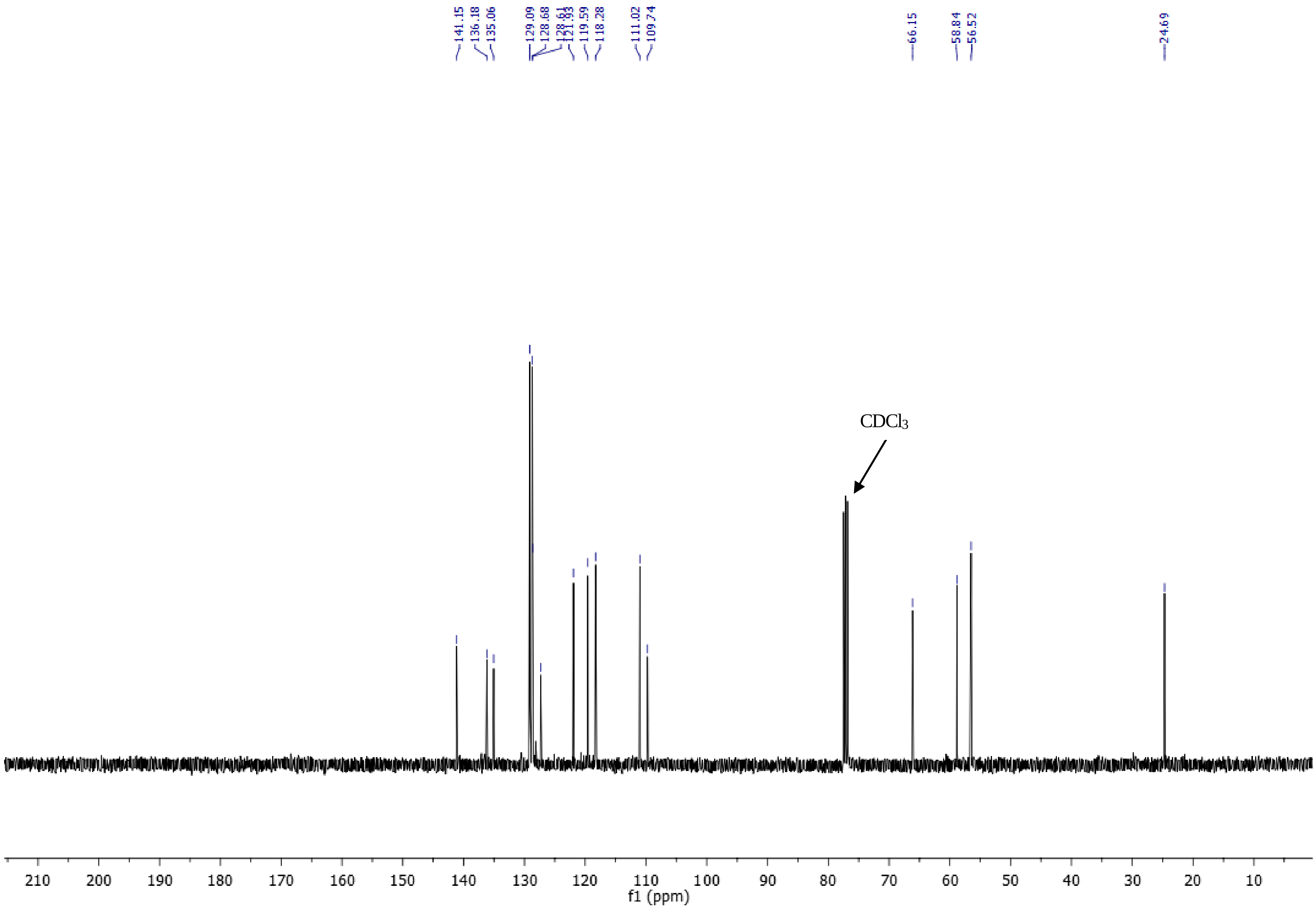

Figure S45. ^{13}C-NMR of compounds **11wa**

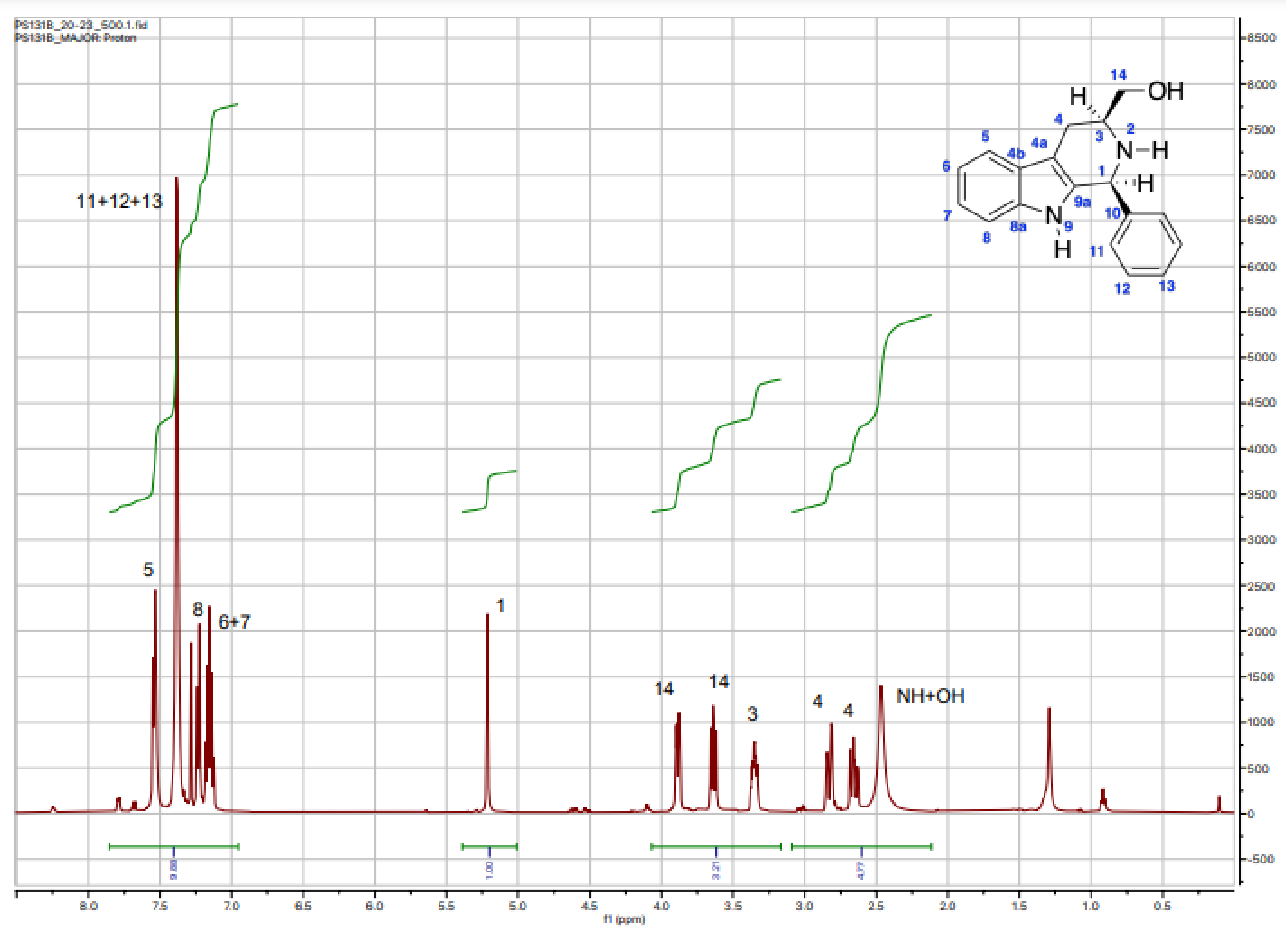

PS131B_20-23_500.1.fid
PS131B_MAJOR: Proton
11+12+13
5
8
6+7
1
14
14
3
4
4
NH+OH
OH
N–H
H
f1 (ppm)

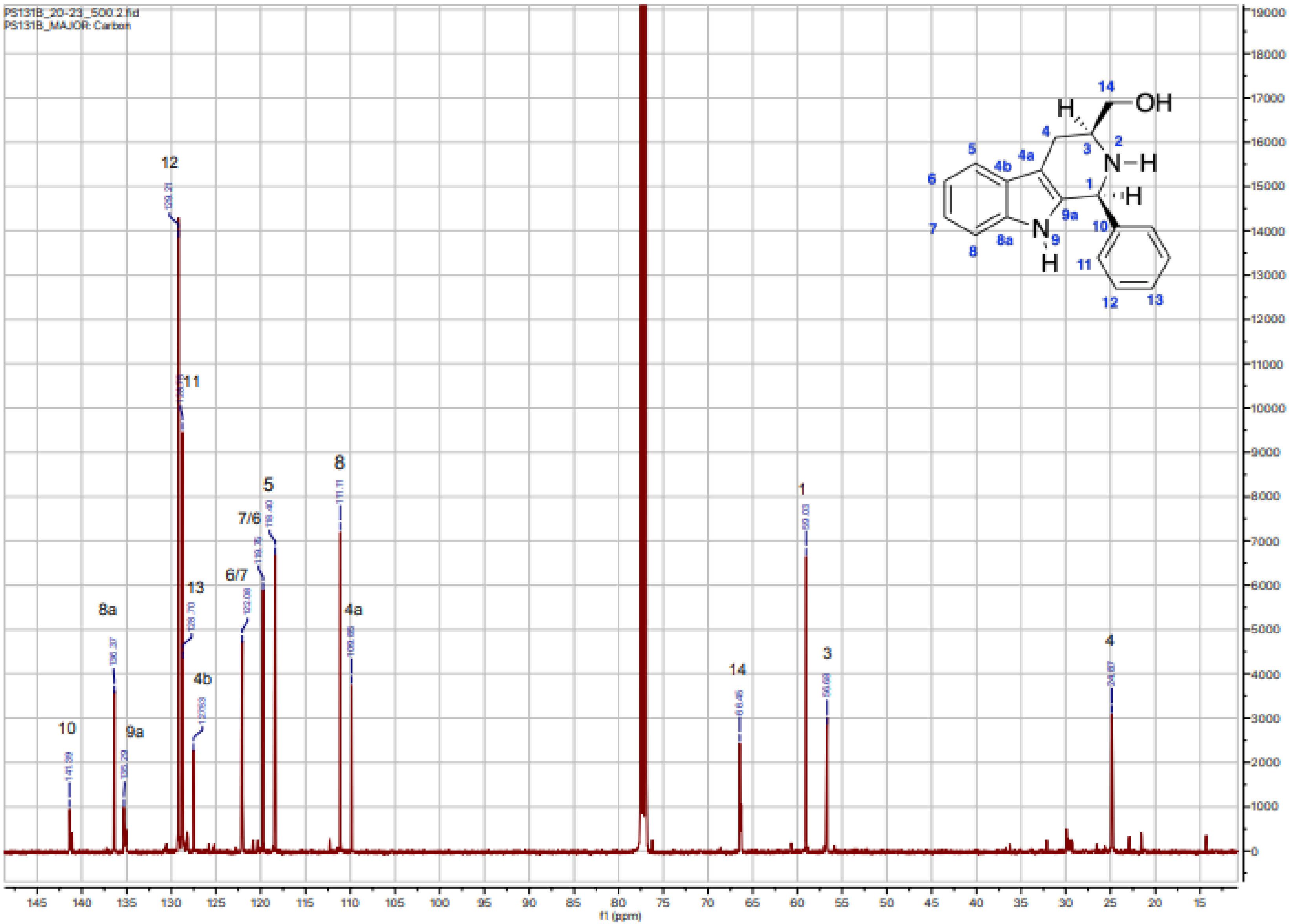
PS131B_20-23_500.2.fid
PS131B_MAJOR: Carbon
12
11
13
8a
10
9a
4b
6/7
7/6
5
8
4a
14
1
3
4
f1 (ppm)
OH
N−H
H

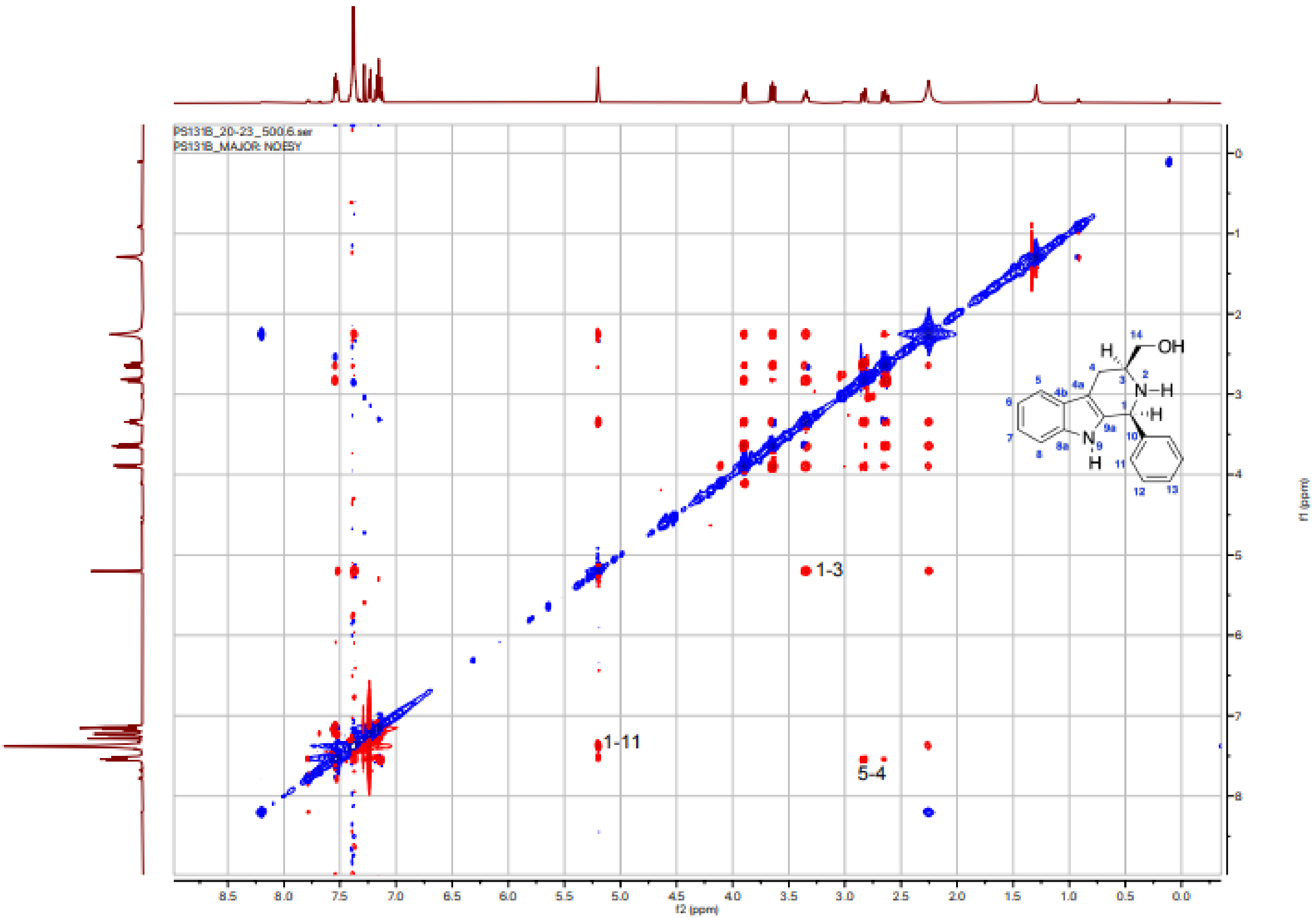

Figure S46. From the top to the bottom: assignment of protons and carbons in ^{1}H-NMR and ^{13}C-NMR, NOESY-NMR of compounds **11wa**

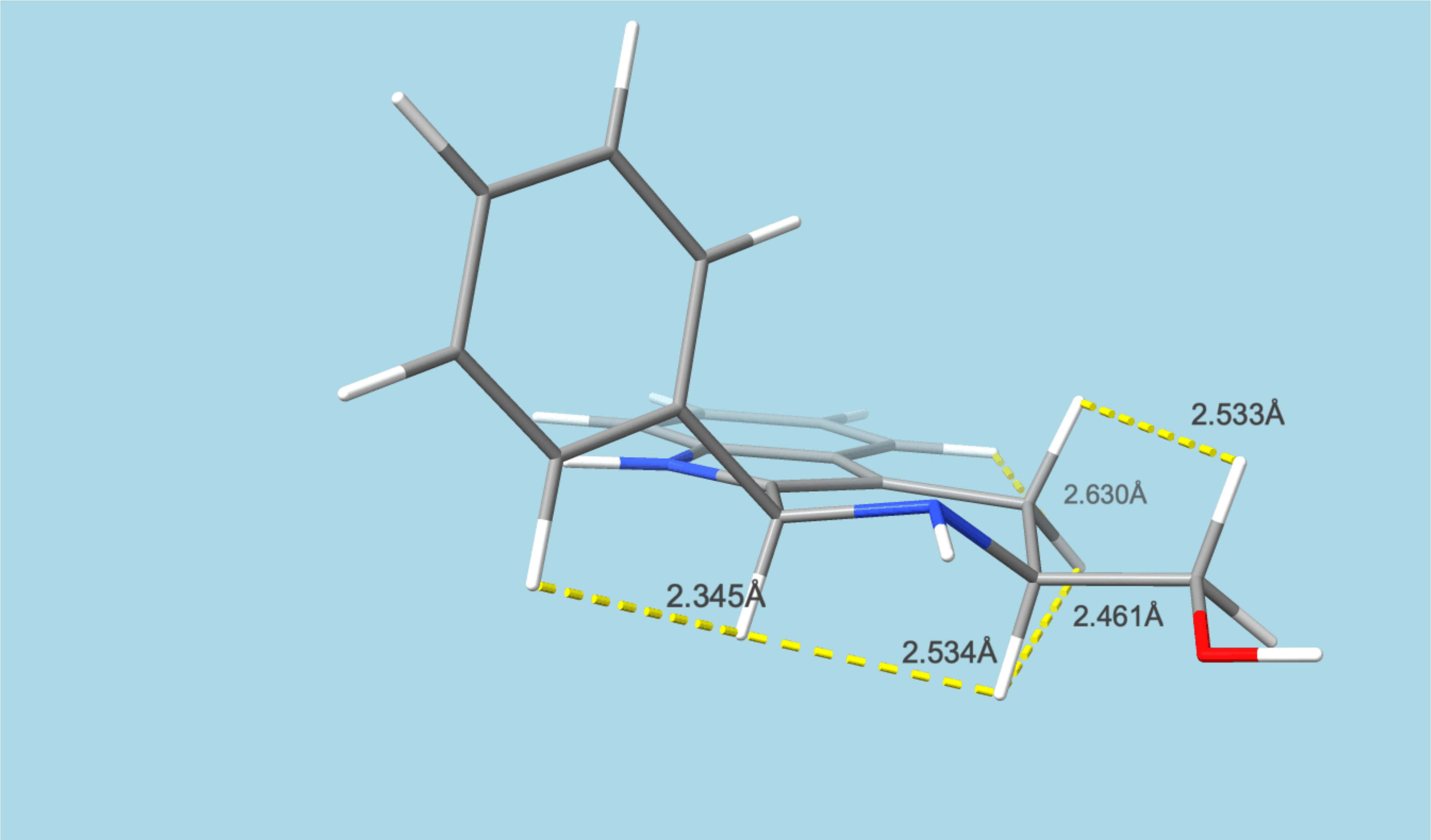

Figure S47. Stick 3D structure of compounds **11wa**

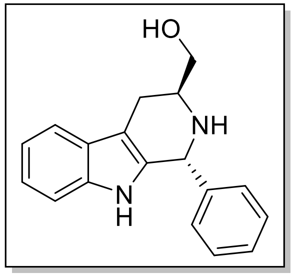

((*1R,3S*)-1-phenyl-2,3,4,9-tetrahydro-1H-pyrido[3,4-b]indol-3-yl)methanol 11wb. Yield = 20% (0.20 mmol, 56 mg, bright yellow solid). **M.p. =** 203-205°C. **Anal. Calcd.** For $C_{18}H_{18}N_2O$ (278.36) C, 77.67; H, 6.52; N, 10.06. **Found**: C, 77.69; H, 6.55; N, 10.08.

GC-MS (EI, 70 eV) = 278.0 ($M^{+\cdot}$), 247, 218 (100), 201, 169, 144, 115, 77.

^{1}H-NMR δ (400 MHz, $CDCl_3$, minor diastereoisomer, ppm) =7.68 (s, 1H), 7.54 (d, *J* = 7.9 Hz, 1H), 7.31 (t, *J* = 7.6 Hz, 4H), 7.18 (ddd, *J* = 20.9, 15.8, 7.4 Hz, 4H), 5.26 (s, 1H), 3.73 (dd, *J* = 10.8, 4.2 Hz, 1H), 3.61 (d, *J* = 8.7 Hz, 1H), 3.27 (m, 1H), 2.90 (dd, *J* = 15.6, 4.9 Hz, 1H), 2.59 (dd, *J* = 15.4, 10.1 Hz, 1H), 1.90 (s, 2H, -OH and -NH).

^{13}C-NMR δ (100 MHz, $CDCl_3$, minor diastereoisomers, ppm) = 136.56, 129.09, 128.96, 128.79, 128.47, 126.98, 122.49, 119.86, 118.52, 111.31, 109.42, 64.06, 54.93, 51.20, 23.39. Spectroscopic data are consistent with those reported in literature.[8]

FT-IR (cm-1) = 3566, 3403, 3055, 2917, 2851, 1456, 1304, 1264, 1027, 736, 700.

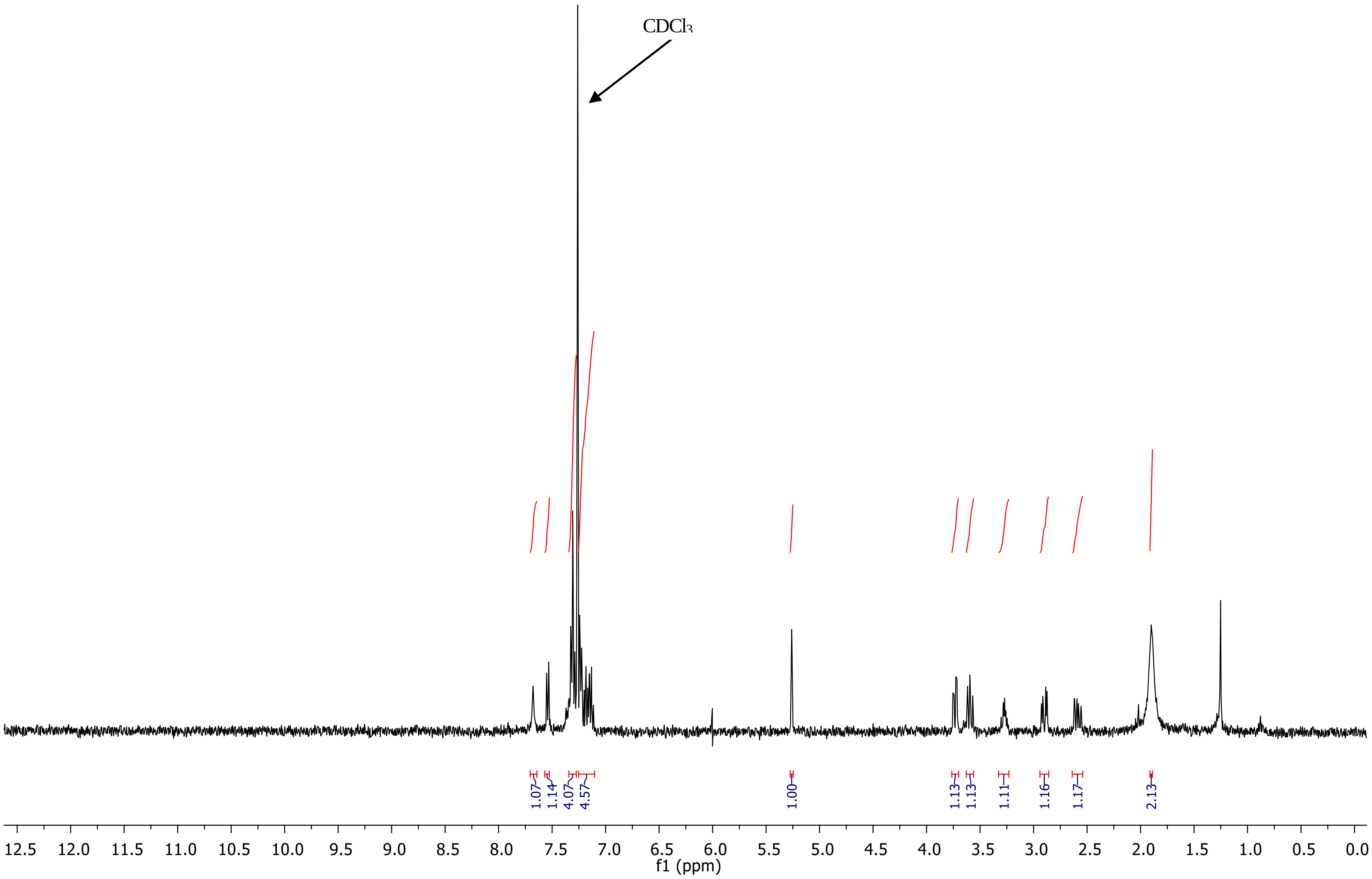

Figure S48. ^{1}H-NMR of compounds **11wb**

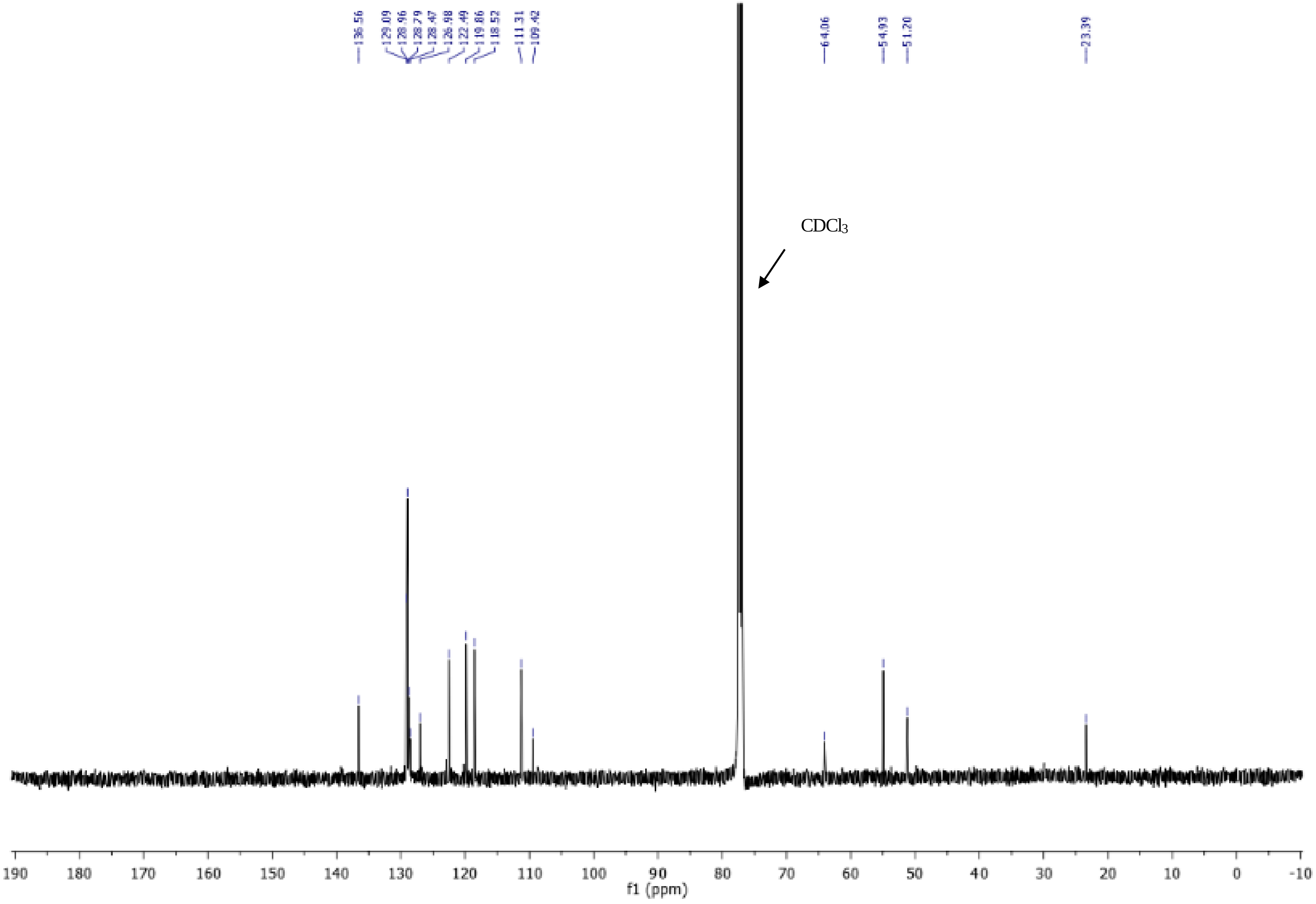

Figure S49. ^{13}C-NMR of compounds **11wb**

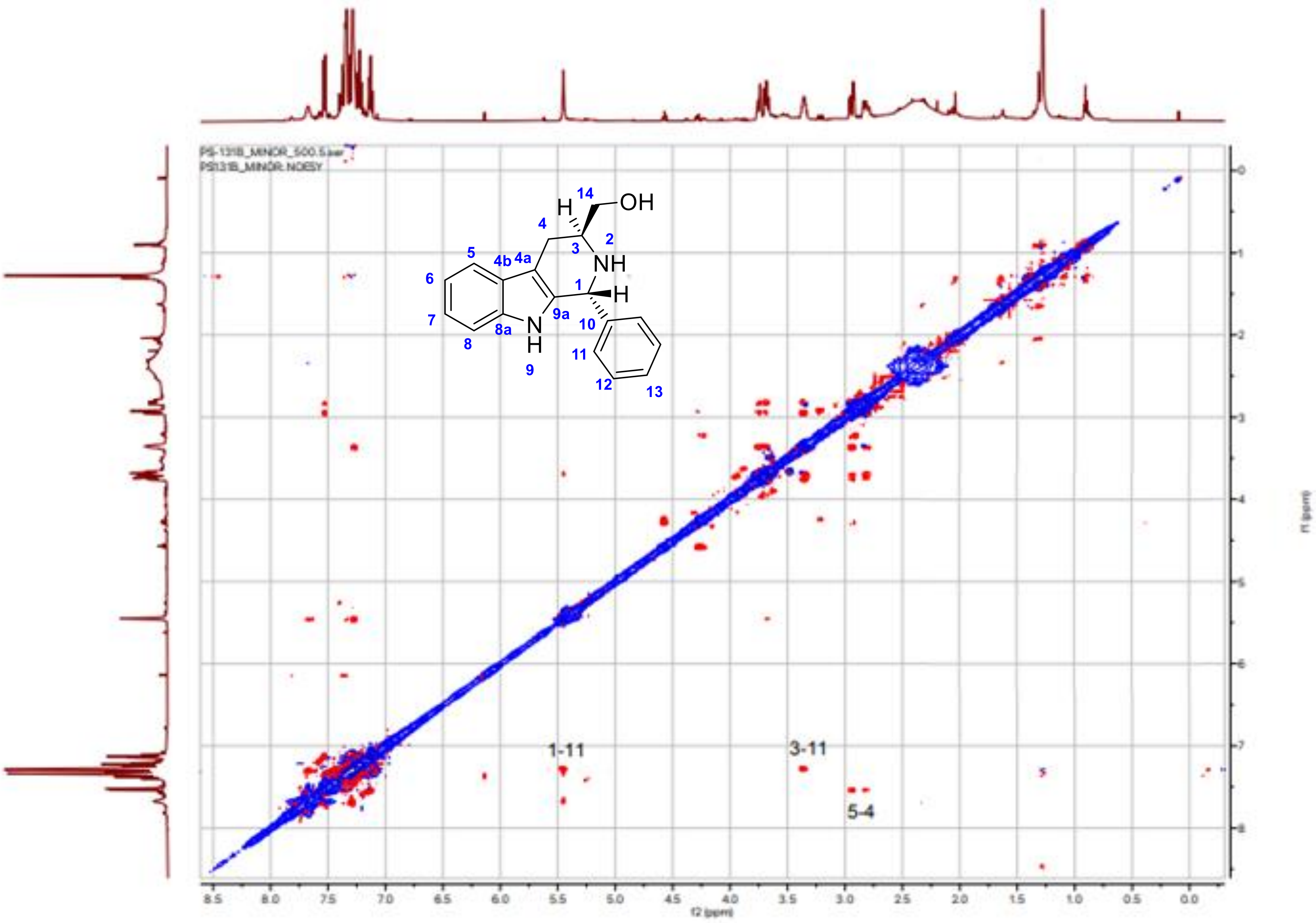

Figure S50. NOESY-NMR of compounds **11wb**

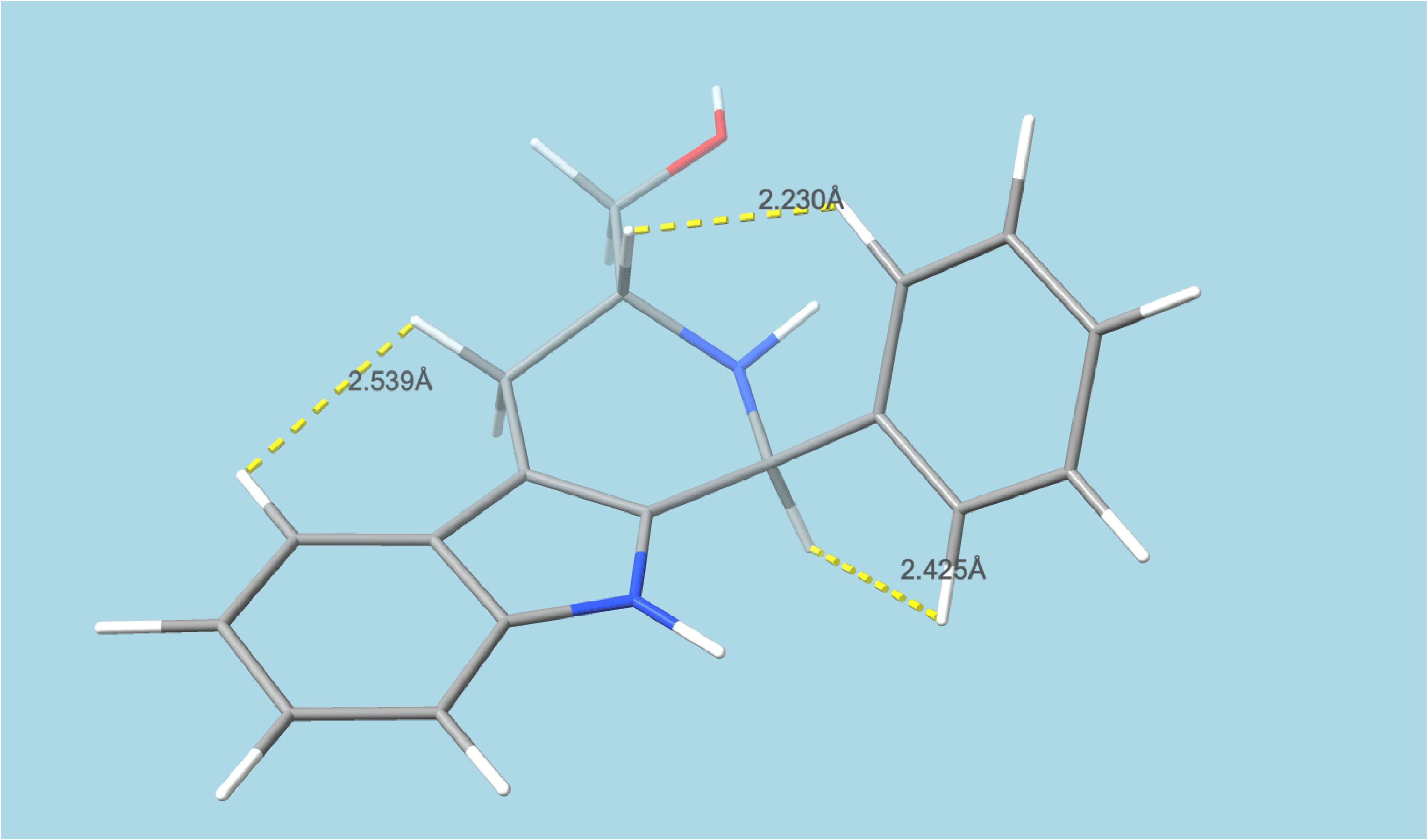

Figure S51. Stick 3D-structure of compounds **11wb**

References:

1. Mahato, R.; Hazra, C. K. Pentafluorophenol (C_6F_5OH) Catalyzed Pictet-Spengler Reaction: A Facile and Metal-Free Approach Towards Tetrahydro-β-Carbolines. *Chem. Eur. J.* **2023**, *29*, e202203924.
2. Liu, H.; Han, F.; Li, H.; Liu, J.; Xu, Q. Selective Construction of Alkaloid Scaffolds by Alcohol-Based Direct and Mild Aerobic Oxidative Pictet–Spengler Reactions. *Org. Biomol. Chem.* **2020**, *18*, 7079-7085.
3. Horiguchi, Y.; Nakamura, M.; Kida, A.; Kodama, H.; Saitoh, T.; Sano, T. A Facile Synthesis of 1,1-Disubstituted 1,2,3,4-Tetrahydro-β-Carbolines Via Trifluoroacetic Acid Catalysed Pictet-Spengler Reaction Using Titatiun(IV) Isopropoxide as an Imination Reagent. *Heterocycles* **2003**, *59*, 691 – 705.
4. Zheng, B.; Trieu, T. H.; Meng, T. Z.; Lu, X.; Dong, J.; Zhang, Q.; Shi, X. X. Cu-catalyzed mild and efficient oxidation of THβCs using air: application in practical total syntheses of perlolyrine and flazin. *RSC Adv.* **2018**, *8*, 6834 – 6839.
5. Kovacs, B.; Forr, E.; Fülop, F. Candida antarctica lipase B catalysed kinetic resolution of 1,2,3,4-tetrahydro-ß-carbolines: Substrate specificity. *Tetrahedron* **2018**, *74*, 6873 – 6877.
6. Zhao, Z.; Sun, Y.; Wang, L.; Chen, X.; Sun, Y.; Lin, L.; Tang, Y.; Li, F.; Chen, D. Organic base-promoted efficient dehydrogenative/decarboxylative aromatization of tetrahydro-β-carbolines into β-carbolines under air. *Tetrahedron Lett.* **2019**, *60*, 800–804.
7. Pawar, G. P.; Chen, H. R.; Barve, I. J.; Shen, L. C.; Sun, M. C. A Direct Synthesis of Substituted Exocyclic 1H-pyrrol-3(2H)-ones by Base-Mediated Multicomponent [3+2] Cycloaddition. *Adv. Synth. Catal.* **2024**, *366*, 473 – 479.
8. Ungemach, F.; Soerens, D.; Weber, R.; Di Pierro, M.; Campos, O.; Mokry, P.; Cook, J. M.; Silverton, J. V. General Method for the Assignment of Stereochemistry of 1,3-Disubstituted 1,2,3,4-Tetrahydro-β-carbolines by Carbon-13 Spectroscopy. *J. Am. Chem. Soc.* **1980**, *102*, 6976 - 6984.
